# Understanding the cost of caring in medicine through personhood: A systematic scoping review of compassion fatigue, vicarious trauma and secondary traumatic stress

**DOI:** 10.1371/journal.pone.0357726

**Published:** 2026-09-10

**Authors:** Nila Ravindran, Nur Amira Binte Abdul Hamid, Priyadarshini A, Afiqah Bte Abdul Rahman, Giovanna Wong, Varsha Rajalingam, Darius Wei Jun Wan, Kaaviya Ramesh, Lalit Kumar Radha Krishna

**Affiliations:** 1 Yong Loo Lin School of Medicine, National University of Singapore, Singapore, Singapore; 2 Division of Cancer Education, National Cancer Centre Singapore, 30 Hospital Boulevard, Singapore, Singapore; 3 Division of Internal Medicine, Singapore General Hospital, Singapore, Singapore; 4 Division of Supportive and Palliative Care, National Cancer Centre Singapore, Singapore, Singapore; 5 SingHealth Duke-NUS Medical Humanities Institute, Singapore, Singapore; 6 School of Health Sciences, University of Dundee, Dundee, United Kingdom; 7 Medical Affairs, National Cancer Centre Singapore, 30 Hospital Boulevard, Singapore, Singapore; 8 Duke-NUS Medical School, National University of Singapore, 8 College Road, Singapore, Singapore; 9 PalC, The Palliative Care Centre for Excellence in Research and Education, Singapore, Singapore; 10 Palliative Care Institute Liverpool, Academic Palliative & End of Life Care Centre, Cancer Research Centre, University of Liverpool, Liverpool, United Kingdom; 11 Health Data Science, University of Liverpool, Liverpool, United Kingdom; 12 Cynthia Goh Palliative Care Institute, National Cancer Centre Singapore, 30 Hospital Boulevard, Singapore, Singapore; Shahid Sadoughi University of Medical Sciences and Health Services Yazd Diabetic Research Centre, IRAN, ISLAMIC REPUBLIC OF

## Abstract

**Background:**

Supporting seriously ill patients and their families through the illness trajectory renders physicians susceptible to compassion fatigue (CF). Traditionally characterized as emotional and physical exhaustion following sustained empathic engagement, emerging research suggests that CF involves deeper disruptions to belief systems and professional identity formation (PIF), alongside an interplay with other trauma-related strain including secondary traumatic stress (STS) and vicarious trauma (VT) within the broader ‘cost of caring’ framework. However, such conceptual understanding remains fragmented and poorly synthesized in physician populations.

**Objectives:**

This systematic scoping review (SSR) maps existing literature on CF, STS and VT among physicians in adult medical specialties, using the Ring Theory of Personhood to examine their defining features, exacerbating and mitigating factors, and existing interventions.

**Methods:**

This SSR employed the modified Systematic Evidence-Based Approach that integrates human and AI-based resources. The research team systematically searched PubMed, Embase, PsycINFO, CINAHL and Google Scholar for articles published from 1^st^ January 2000−30^th^ June 2025, supplemented by searches on Elicit AI Pro. Eligible articles underwent content and thematic analyses, augmented by ChatGPT o3 Pro.

**Results:**

Of 18,396 database abstracts and 497 Elicit AI Pro abstracts identified, 986 non-duplicate articles underwent full-text review, with 122 meeting the inclusion criteria. CF, STS and VT encompass moral, cognitive, relational and identity-related dimensions that shape belief systems and PIF. Sharing overlapping features, these constructs sit along a continuum, where early features of CF—including exhaustion, depersonalization and boundary erosion—may progress toward STS’ characteristic post-traumatic stress-type intrusions. Unaddressed, these acute reactions can evolve into enduring cognitive and existential alterations denoting VT. This trajectory can be interrupted by individual and environmental protective factors.

**Conclusion:**

Reframing CF, STS and VT as a continuum of escalating strain disrupting physicians’ PIF highlights the need for stage-specific interventions and effective assessment tools that better capture this expanded notion.

## Introduction

Caring for patients with serious illnesses and supporting their families through their illness trajectory predispose physicians to a host of emotional and psychological sequelae, including emotional and physical fatigue, transference, boundary challenges, low mood, diminished confidence and empathy, poor coping, anxiety, cynicism and detachment [1–6]. These experiences are frequently positioned in earlier literature as a downward spiral with implications for physician well-being, patient safety and satisfaction, clinical outcomes and quality of family support [7]—traditionally explained through constructs such as moral distress (MD) [8], compassion fatigue (CF) [9,10], secondary traumatic stress (STS) [11] and vicarious trauma (VT) [12] that form the ‘cost of caring’ [13–16].

However, the adequacy of these constructs is increasingly challenged in emerging research [17–20]. New evidence suggests a need for conceptual updating of each component to more accurately reflect current clinical realities [21]. Our recent review of MD [22], for example, underscores prevailing inaccuracies and limitations when this concept—originally developed within nursing practice [8] arising from the obstruction of enacting perceived morally correct actions, often rooted in hierarchical powerlessness—is extrapolated to medical practices. Similarly, Tabor et al. [17] call attention to the interchangeable and often incorrect use of terminology surrounding VT. Recognizing these conceptual limitations is imperative, given the persistent distress reported by physicians caring for patients with serious or terminal illness. Accordingly, there is pressing need to re-examine the next major component within this broader *cost of caring* framework: CF.

Attention upon the cost of compassion is needful, in light of its integral role in building trusting doctor-patient relationships and promoting holistic patient-centered care [23,24]. Indeed, recent studies have found that initiating a “virtuous and intentional response to know a person, to discern their needs, and ameliorate their suffering, through relational understanding and action” [25] can, over time, incite emotional and empathetic strain, as well as physical, ethical, moral and existential distress that may result in CF among physicians [1,16,26–28]. First coined by Joinson [9] in her observation of emergency nurses in trauma care and later expanded by Figley [29] as a “state of exhaustion and dysfunction, biologically, physiologically and emotionally, as a result of prolonged exposure to compassion stress”, CF can trigger an erosion of compassion and empathy, desensitization to patient needs and care, loss of professional fulfilment and, in severe cases, attrition or early retirement from the healthcare field [13,30–33].

CF is also unlikely to occur in silo [34]. The very notion of compassion as “feeling and acting with deep empathy and sorry for those who suffer” [35] implies an intrinsic link to STS and VT, particularly when profound empathy and overidentification [26] can blur personal and professional boundaries and lead to a conflation of patients’ or families’ distress with one’s own. VT was first described by McCann and Pearlman [12] in understanding the psychological aftermath in therapists, arising from unregulated cognitive, affective and behavioral responses to their clients’ trauma. STS was later introduced by Figley [11] as the stress emerging from knowledge of another’s trauma, coupled with the desire or effort to help. This intrinsic connection to CF is embodied in Stamm’s [36] seminal Professional Quality of Life (ProQOL) framework that positions STS as one of two core components of CF.

Pertinently, CF, STS, and VT have been shown to reshape how physicians view the world and interact with others [16,37–39]—signaling deeper effects beyond immediate distress. We posit that this reconfiguration of outlook is brought about by changing belief systems—comprising moral values, ethical principles, familial mores, cultural norms, attitudes, decisional preferences and responsibilities—that collectively form one’s personhood (what makes you, you) [40–42]. Because these belief systems form the foundation of personhood, shifts in them may alter how physicians understand themselves and their place within the world [43,44]. In turn, such shifts may influence professional identity formation (PIF), which encompasses how physicians think, feel, and act as professionals; inhabit their roles and responsibilities; and embody shared professional values [45–48]. CF, STS, and VT may therefore carry wider implications for physician wellbeing, identity, and patient care than is currently acknowledged.

Despite extensive attention within nursing and psychology, the landscape of CF, STS, and VT in medicine remains fragmented and poorly synthesized. In particular, the potential implications for belief systems, personhood, and PIF have yet to be mapped or conceptually integrated. To address this gap, we propose a systematic scoping review (SSR) to clarify what is known about CF, STS, and VT in medicine.

This review will address the following questions:

1. What is known about compassion fatigue (CF), secondary traumatic stress (STS), and vicarious trauma (VT) in medicine?
2. What factors exacerbate and attenuate CF, STS and VT?
3. What interventions have been identified to mitigate the impact of CF, STS and VT?

It is hoped that synthesizing this literature will provide a more contemporary and clinically relevant understanding of CF, STS and VT within the *cost of caring* framework and inform evidence-based strategies to support physicians’ wellbeing, PIF and patient care.

## Materials and methods

### Theoretical lens

This SSR employs Moss and Haertel’s [49] principle of methodological pluralism, which supports the integration of positivist and constructivist paradigms. A positivist paradigm allows the adoption of PRISMA guidelines [50] and the AI-supplemented modified Systematic Evidence-Based Approach (mSEBA) to ensure transparency, reproducibility and comprehensive data mapping and synthesis. Concurrently, a constructivist ontology and a relativist epistemology recognize CF, STS and VT as sociocultural constructs emerging from evolving belief systems and unique contexts, and that knowledge about them is co-constructed through physicians’ experiences and shared narratives [51,52].

Methodological pluralism also supports the concurrent use of the Krishna-Pisupati Model (KPM) [53,54] to sensitize the analysis to changes in personhood and PIF. This combined theoretical orientation facilitates both thorough evidence synthesis and interpretive engagement with context-dependent constructs.

#### The Krishna-Pisupati model.

The KPM integrates the Ring Theory of Personhood (RToP) [40,41,55], which advances four interrelated domains of belief systems that constitute personhood:

- Innate Ring: contains spiritual, existential and demographic belief systems;
- Individual Ring: contains belief systems that inform conscious function including behavior, emotions and personality;
- Relational Ring: contains belief systems that govern close personal, familial and social relationships;
- Societal Ring: contains belief systems tied to sociocultural, professional, legal and ethical norms, rights, expectations, roles and responsibilities.

Building on the RToP, the KPM outlines how belief systems may become misaligned following new experiences (see S1 Fig. Krishna-Pisupati Model). Such misalignment may take the form of *disharmony* when emerging belief systems oppose existing ones within a single ring, or *dyssynchrony* when these conflicts occur across two or more rings [43,53]. If unaddressed, this dissonance can reshape thinking, decision-making and conduct and, in turn, negatively influence PIF [14,56].

Central to mediating these effects is the *internal compass*—an internalized schemata of moral, ethical and professional values that guide physicians to think, feel and act in ways that reflect their evolving sense of self [14,56–58]. A mature *internal compass* enhances sensitivity to possible changes within belief systems and enables the physician to appraise such dissonance, strengthening their willingness to make meaningful adaptations to sustain PIF [58].

Given the emotionally charged nature of prolonged empathic engagement, we posit that experiences with CF, STS and VT can precipitate changes in belief systems.

### The modified Systematic Evidence-Based Approach (mSEBA)

The mSEBA is an adaptation of the Systematic Evidence-Based Approach (SEBA), originally designed to guide reproducible and transparent systematic scoping reviews [15,59–62]. This modified version integrates the use of artificial intelligence (AI) tools, such as ChatGPT and Elicit AI Pro, to triangulate literature searches and analysis, as well as finesse data in parallel with traditional manual approaches.

The mSEBA methodology adopts an Interpretative Approach to Analysis, informed by phenomenological and hermeneutic lenses, that extends beyond rote summarization to *interpret* what findings mean. A phenomenological lens in this context considers how physicians make meaning of their experiences—shaped by personal motivations, life experiences, evolving belief systems and the personal, professional, ethical, psychosocial, cultural, legal and societal contexts in which they practice. A hermeneutic lens recognizes that researchers’ own experiences can influence data interpretation. This underpins our inclusion of reflexivity and dialogue, including team-based independent searches and analyses, as well as the use of Sandelowski and Barroso’s [63] “negotiated consensual validation” to minimize personal biases and maintain a balanced review.

The mSEBA unfolds across six stages, outlined in S2 Fig. Modified Systematic Evidence-Based Approach.

#### Stage 1 of mSEBA: The systematic approach.

An expert team of medical librarians, local educational experts and clinicians guided the research team in navigating all stages of mSEBA. This inclusion of an expert team served to strengthen the reliability and transparency of the research process whilst adhering to the PRISMA-ScR guidelines (see S1 Appendix PRISMA Checklist).

i. **Determining the research question and inclusion criteria**

The fragmented CF, STS and VT landscape in medicine presented a significant research gap, leading to the formulation of the following primary and secondary research questions:

1. What is known about CF, STS and VT in medicine?
2. What factors exacerbate and attenuate CF, STS and VT?
3. What interventions have been identified to mitigate the impact of CF, STS and VT?

A clear inclusion and exclusion criteria were established based on the Population, Context, Concept (PCC) framework (Table 1). Here, we focused on physicians in adult medical specialties and excluded pediatric and surgical specialties due to distinct variations in relational dynamics, ethical contexts and clinical settings that could introduce substantial conceptual heterogeneity. Pediatric care, for example, involves triadic child-parent-clinician interactions [64–66] while surgical specialties are often characterized by procedure-focused encounters rather than sustained relational care [67]—distinctions that likely shape CF, STS and VT in ways not directly comparable to those experienced by physicians in adult medical specialties.

**Table 1:** Population, Context, Concept (PCC) Study Design.

	Inclusion Criteria	Exclusion Criteria
**Population**	• Physicians in all adult medical specialties that care for patients with serious illnesses (We assumed that this larger perspective would capture wider concepts of the *cost of caring)*	• Medical students • Nursing, psychology, or allied health professionals • Physicians in pediatrics and surgical specialties, including obstetrics
**Context**	• All study designs: qualitative, quantitative and mixed methods studies • Clinical practice • Published 1^st^ January 2000–30^th^ June 2025 • Systematic reviews, literature reviews, narrative reviews, grey literature	• Research or academic settings where patient care is not practiced
**Concept**	• Compassion fatigue, secondary traumatic stress, vicarious trauma, from prolonged exposure to suffering and traumatized patients • Impact of compassion fatigue, secondary traumatic stress and vicarious trauma on physicians, patients, and healthcare system • Risk factors and protective factors for the cost of caring • Strategies to mitigate/treat the cost of caring	• Distress arising from the work environment that is not related to the doctor-patient relationship

ii. **Human-led literature searches**

Applying the search strategy outlined in Table 2, three pairs of research team members performed a systematic search of relevant articles published between 1^st^ January 2000 and 30^th^ June 2025 in PubMed, Embase, PsycINFO, CINAHL and Google Scholar. The decision to focus on studies published from 2000 onward reflected a balance between achieving a thorough review and ensuring that included studies represented current perspectives on CF, STS and VT. To further enhance the review, snowballing of reference lists of eligible studies was also performed for additional relevant literature. Each team subsequently consolidated the list of articles identified from these searches.

**Table 2:** Example of Search Strategy.

**PubMed**
After publication date filter (from 1^st^ Jan 2000–30^th^ June 2025) and language filter (English) (Physician*[Mesh] OR Clinician*[Title/Abstract] OR physician*[Title/Abstract] OR Doctor*[Title/Abstract]) AND (“Compassion Fatigue”[Mesh] OR compassion fatigue*[Title/Abstract] OR empath* stress OR empath* fatigue OR ((stress*[Title/Abstract] OR trauma* [Title/Abstract] OR fatigue) AND (secondary[Title/Abstract] OR empath* [Title/Abstract] OR vicarious[Title/Abstract])))
**Google Scholar**
After publication date filter (from 2000 to 2025) and language filter (English) allintitle: doctor OR doctors OR physician OR physicians OR residents OR resident OR clinician OR clinicians “compassion fatigue”

To reach an agreement on the set of articles to be reviewed for inclusion in the study, a three-step process was adopted. Step one, each team screened the title and abstracts of their identified articles to determine study relevance. This step was performed on EndNote which facilitated the management of large quantities of data. Step two, each team created an independent list of articles that passed the title and abstract screening. Step three, the research teams convened to compare their lists. Sandelowski and Barroso’s [63] principle of negotiated consensual validation was practiced, which allowed “research team members [to] articulate, defend and persuade others of the ‘cogency’ or ‘incisiveness’ of their points of view”. In this way, consensus on the shortlisted articles to proceed to a full-text review was reached.

iii. **Extracting and charting**

Guided by the inclusion and exclusion criteria outlined in Table 1, each team performed a full-text sieve of the shortlisted articles to assess their suitability for inclusion. This data was recorded on Microsoft Excel to keep track of articles that met or did not meet the eligibility criteria. Through regular group discussions that adopted the practice of negotiated consensual validation, 114 articles were determined to have met the eligibility criteria.

iv. **AI-supplementation**

mSEBA is marked by its iterative cycle of human-led and AI-assisted searches to minimize the risk of data omission whilst expanding the breadth of the review. This stage thus saw the use of Elicit AI Pro to triangulate and supplement manual searches. Elicit AI Pro’s semantic search capabilities and advanced filtering functions enhanced the efficiency and sensitivity of the search process.

An independent researcher applied the same search protocols on Elicit AI Pro as those used in the human-led searches to identify articles uncaptured manually. Of the 497 articles retrieved through Elicit AI Pro, 21 overlapped with 114 articles manually included by the research team. The remaining 476 articles were independently screened by the researcher through Elicit AI Pro’s abstract screening function, calibrated to the PCC framework outlined in Table 1. Eight articles met the inclusion criteria and were selected to be included in the review.

v. **Quality appraisal**

The research team consolidated the finalized articles, summarizing their key findings and performing quality appraisals using the Medical Education Research Study Quality Instrument (MERSQI) [68] and the Consolidated Criteria for Reporting Qualitative Studies (COREQ) [69] for methodological transparency and balanced synthesis. These finalized articles are detailed in S2 Appendix Final List of Included Articles.

#### Stage 2 of mSEBA: Split approach.

The finalized articles subsequently underwent concurrent thematic and content analyses by the same three independent teams of researchers. This combined approach accounted for the limitations of each method (e.g., by mitigating the subjectivity in thematic analysis with the objectivity in content analysis and attenuating the lack of depth in content analysis with thematic analysis) to safeguard a more holistic and robust output of data.

The first team adopted Braun and Clarke’s [70] approach to thematic analysis, which prompted the organic emergence of themes without reliance on predetermined groupings. Team members independently reviewed each included article for relevant findings, constructing codes from the text’s immediate meaning [71]. Through an iterative process, emerging codes were linked to prior ones, synthesizing semantic themes derived directly from the raw data. Thereafter, the team convened to discuss their independent findings, shortlisting the final list of themes through negotiated consensual validation.

Simultaneously, the second team of researchers independently performed Hsieh and Shannon’s [72] directed content analysis. This approach utilized pre-existing coding frameworks to assuage concerns regarding inconsistency, incoherence and omission of negative results observed in thematic analysis. Here, the codes were derived from Sinnathamby et al.’s [61] and Krishna et al.’s [73] studies on CF and death and dying. New codes were created for data that did not align with the priori codes. This was followed by the merging of complementary codes into broader categories. Similarly, negotiated consensual validation was used to reach consensus on the final categories [63].

Lastly, the third team of researchers employed ChatGPT o3 Pro to assist with data analysis. PDFs of all 122 included articles identified from both the manual and AI-assisted searches were uploaded to ChatGPT o3 Pro on June 23, 2025.

ChatGPT o3 Pro was tasked to analyze the included articles using the categories identified through directed content analysis, serving as a form of triangulation. Prompts were designed to exclusively confine the model’s attention to the included articles, rather than drawing from external data. All AI-assisted analyses made use of data uploaded by the research team only and external memory, browsing and retrieval were not used during the analyses.

i. **Sequential upload of included articles**

The included articles were uploaded in batches of 10 due to upload limitations on ChatGPT o3 Pro. Using the prompts shown in Table 3, the AI conducted the analysis for each batch of articles and compiled a feature matrix for the characterization of CF, STS and VT mapped to the RToP as its output. The prompt to upload and characterize CF, STS and VT was then rerun for subsequent batches of articles. ChatGPT o3 Pro was instructed to add onto its previous output with each upload. The categories identified in the feature matrix remained consistent. Once all articles had been sequentially uploaded, ChatGPT o3 Pro could draw information from all 122 articles at the same time. These analyses were then consolidated into summary tables that are presented in Domains 1, 2 and 3, as indicated by the summary of outputs in Table 3.

**Table 3:** AI Transparency: Summary of Key Prompts and Outputs in Search and Analysis.

Function of Prompt	AI Tool	Simplified Prompts	Summary of Outputs	Use of Output in the Study
Search	Elicit AI	“What are the risk and protective factors associated with Compassion Fatigue (CF), Secondary Traumatic Stress (STS) and Vicarious Trauma (VT?) and what interventions have been identified to mitigate the impact of CF, STS and VT?”	497 articles were identified.	All relevant articles were identified through Elicit AI’s systematic review function. This search yielded eligible articles that may not have been identified in the manual searches.
Analysis	ChatGPT o3 Pro	“Please go through all the zip folders and draw all elements of CF, VT and STS and please reference each and every account.” “Please also look for aspects of PIF, RToP and the internal compass related to the new characterization of CF, VT and STS, please be detailed and thorough as this is part of a systematic scoping review.”	Provided an outline of scope and method used in compiling the included articles. Clustering and mapping of features, generated as a consolidated feature matrix.	The consolidated feature matrix was used to guide the AI in searching for the most relevant characteristics of CF, VT and STS throughout the included articles, used later to generate the characterization tables for each.
		“Compile a table each for CF, VT and STS with the characteristics in one column and each characteristic in a row with the corresponding references (APA style).” “I will upload them in batches of 10 pdfs”	Referenced tables for the characterization of CF, VT and STS were generated individually. The included articles were uploaded in batches of 10 and each row of the tables were sequentially built upon with each upload.	This output was the first iteration of the characterization tables in Domains 1, 2 and 3, though some articles were not included by the AI.
		“Consolidate all the data into the 3 tables requested and add features of the RToP, internal compass and PIF in each table, list all mention of each characteristic and all references in APA style”	Added new columns indicating the effects on the rings of the RToP, internal compass and PIF.	This output built upon the previously generated tables by adding new columns for the analysis of RToP, internal compass and PIF respectively.
		“List all the missing articles”	Identified and provided a list of articles missing from the previously generated tables.	This output aimed to guide the AI in identifying the missing articles to be added in the next iterations of the characterization tables.
		“These are the missing articles, please verify these findings”	Missing articles that were reuploaded were included in newly generated summary tables.	The missing articles from the previously generated list were uploaded by the researcher and added to the characterization tables.
		“Please provide 1. Consolidated tables for CF, SRS and VT each 2. Ensure APA referencing 3. Provide the tables and the lists of the references as a pdf and word document”	Generated the finalized tables with all articles included and referenced.	This output was the final iteration of the characterization tables presented in Domains 1, 2 and 3 of the Results.

ii. **Use of prompts to consolidate outputs**

To maintain AI transparency, Table 3 details how ChatGPT o3 Pro was used in this study, including verbatim simplified prompts and output summaries. These prompts were shortened to exclude contextual information regarding the theoretical lens used.

iii. **Verification of AI outputs**

All outputs were reviewed by research team members to identify potential hallucinations, omissions or errors. This process of verification is outlined below:

1. Review of the feature matrix: The research team reviewed the feature matrix compiled by ChatGPT o3 Pro once all 122 articles were uploaded. This involved i) checking if all 122 articles were referenced, ii) ensuring all outputs were correctly referenced without hallucinations and iii) the pertinent features identified matched with the content within each referenced article.
2. Inclusion of missing articles: 15 included articles that were missing in the feature matrix were identified and reuploaded to ChatGPT o3 Pro for inclusion. The process of ensuring all outputs were correctly referenced and aligned with the content of the articles was repeated.
3. Review of consolidated summary tables: The final consolidated summary tables were similarly reviewed by the research team to ensure accurate referencing. Team members cross-checked the citation details provided in each output against the list of included articles to ensure that no non-existent articles or fabrications were introduced. In total, 35 citation errors were identified through manual checks and modified to include the correct details.
4. Comparison of AI-assisted analyses with human-led analyses: The AI-driven findings were compared with those derived from human-led analyses. This process was overseen by a senior researcher, who evaluated the corroboration by ChatGPT o3 Pro and determined if there were new themes or omitted categories to be considered.

While the use of AI aided in streamlining data, the research team retained full autonomy in coding decisions and synthesis of results.

Inter-rater reliability was not calculated as coding was undertaken as part of a research training process for medical students in the research team, who worked under guidance of the senior author. Analytic rigor was ensured through iterative discussions and consensus-building rather than statistical agreement.

#### Stage 3 of mSEBA: The Jigsaw Perspective.

The notion that complementary qualitative data gives “a richer, more nuanced understanding of a given phenomenon” [49] laid the foundation for the Jigsaw Perspective. Visualizing the identified themes and categories as pieces of a jigsaw puzzle, the research team merged complementary pieces into broader thematic/categorical units that portrayed a fuller picture of the data [74]. This process was guided by Phases 4–6 of France et al.’s [74] adaptation of Noblit et al.’s [75] seven phases of meta-ethnographic approach.

#### Stage 4 of mSEBA: The Funneling Process.

The merged themes/categories were subsequently compared with the tabulated summaries of the included articles to ensure an accurate representation of the funneled data. This then led to the formation of the resulting domains.

#### Stage 5 of mSEBA: Analysis of evidence-based and non-data-driven literature.

The final step involved the comparison of findings from peer-reviewed publications with those from grey literature to assess potential biases. Yielding similar results, bias from non-data-driven literature was deduced to be minimal.

## Results

**Fig 1:**
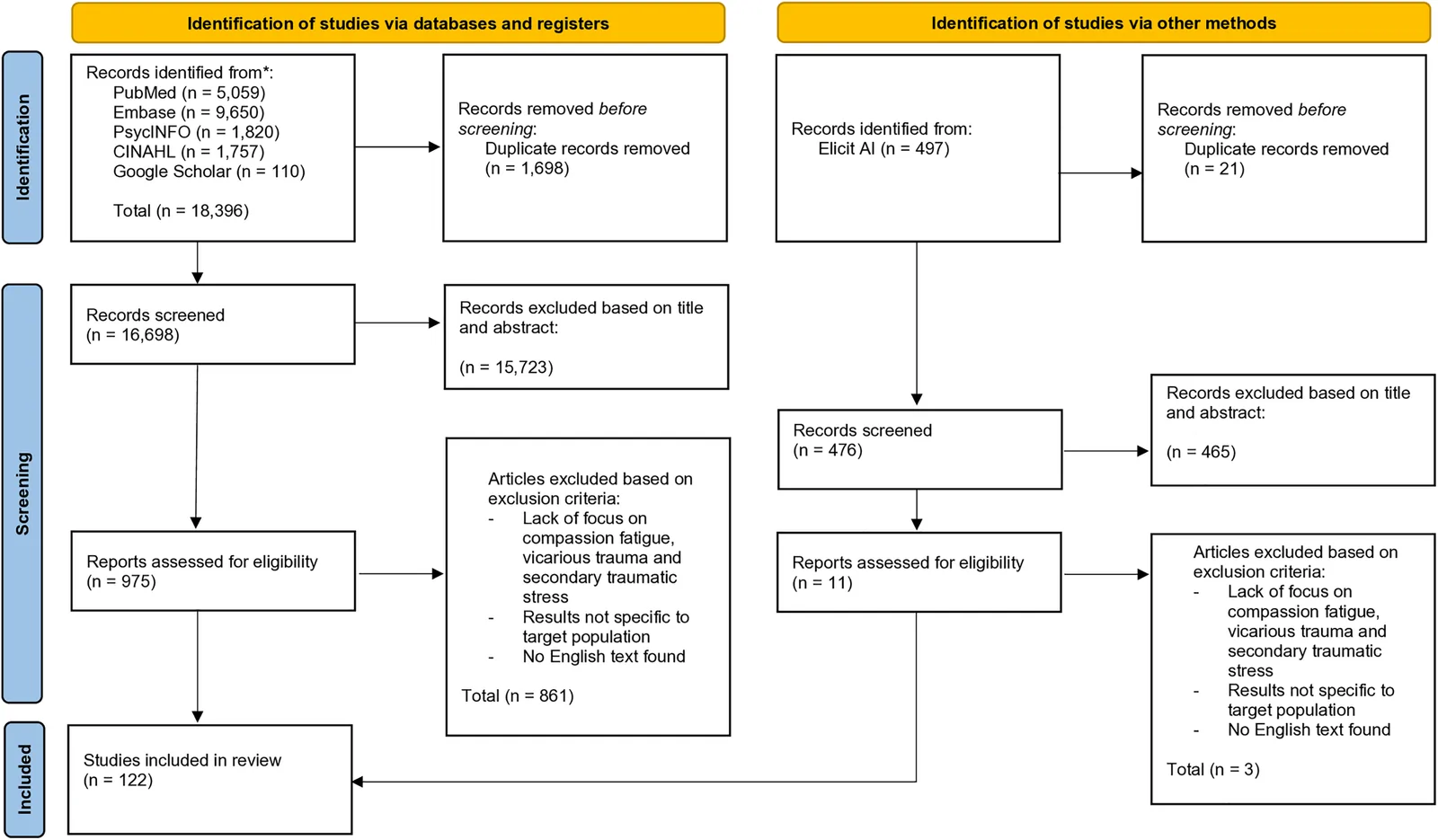
PRISMA Flowchart.

A total of 18,396 abstracts from databases and 497 abstracts from Elicit AI were identified, totaling to 18,893 abstracts. A total of 986 non-duplicate full-text articles were reviewed and 122 studies met the inclusion criteria. The PRISMA flowchart depicting the selection process is shown in Fig 1.

Five key domains emerged from the analysis: 1) characteristics of CF; 2) characteristics of STS; 3) characteristics of VT; 4) risk and protective factors; and 5) interventions. Features attributed to CF, STS and VT were mapped according to KPM lens, offering an opportunity to compare traditional notions of these constructs with current thinking.

### Domain 1: Characteristics of compassion fatigue

Notions of CF have evolved since Joinsons’s and Figley’s initial characterization of it as a state of physical and emotional exhaustion accompanied by a diminished capacity for compassion and empathy. Mapping CF onto the KPM reveals deeper disruptions across the Individual, Relational and Societal Rings, with both personal (e.g., emotional dysregulation, cognitive overload, diminished self-worth and confidence) and professional (e.g., decision-fatigue, impaired clinical judgment, compromised physician-patient relationships, imposter syndrome) ramifications. These personal and professional consequences of prolonged empathic engagement are mutually reinforcing and can ultimately influence the quality of patient care. Table 4 outlines the current characteristics of CF in greater detail.

**Table 4:** Current Characterizations of Compassion Fatigue.

Characteristics	Description
Emotional and Physical Exhaustion [61,76,77]	Emotional exhaustion and somatic complaints disrupt the Individual Ring and the internal compass by limiting reflective capacity, eroding self-efficacy and weakening moral judgment. These effects may also extend to the Relational Ring by promoting social withdrawal and straining interpersonal and professional relationships.
Depersonalization / Diminished Empathy [78,79]	Depersonalization and diminished empathy undermine the connection that sustains compassionate care as physicians maintain distance from patients, destabilizing the Relational Ring. This detachment may also clash with professional expectations of empathy and patient-centered care embodied in the Societal Ring.
Guilt, Helplessness and Moral Residue [16,80]	Feelings of guilt and helplessness erode confidence, self-worth and emotional regulation, straining the Individual Ring and the internal compass. The resulting moral residue may also cause dyssynchrony between personal ethics in the Individual Ring and professional expectations in the Societal Ring.
Bereavement Overload / Cumulative Grief [81,82]	The cumulative weight of bereavement diminishes emotional balance and resilience, undermining the Individual Ring. It may also spill into the Relational Ring through reduced empathic engagement with patients and withdrawal from personal relationships.
Decision‑Fatigue / Cognitive Overload [78,80]	Decision-fatigue disrupts the Individual Ring and weakens the internal compass through the impairment of moral judgement, reflective capacity and self-regulation. It may also compromise adherence to professional standards, straining the Societal Ring.
Boundary Erosion (Over / Under Involvement) [61,83]	The blurring of professional and personal boundaries disrupts the Relational Ring. Over-involvement may lead to enmeshment while under-involvement risks detachment and declining empathy, destabilizing the Individual Ring.
Moral Distress / Moral Injury [16,61,84]	Moral distress introduces dyssynchrony between personal values and professional expectations, destabilizing both the Individual and Societal Rings and distorting professional identity.
Emotional Labor / Surface Acting [85]	Emotional labor and surface acting cause disharmony between authentic and displayed emotions in the Individual Ring. This may extend to the Relational Ring over time and disrupt meaningful patient relationships through impersonal interactions.
Maladaptive Perfectionism / Imposter Feelings [86]	Maladaptive perfectionism and imposter feelings unsettle the Individual Ring by triggering self-doubt and self-criticism and eroding confidence. This may lead to a questioning of professional calling, roles and responsibilities, undermining alignment with professional values and expectations in the Societal Ring.

Taken together, these findings highlight an evolved understanding of CF that transcends its traditional conceptualization to reveal a multi-dimensional construct with moral, cognitive, relational and identity-related dimensions that reshape the *internal compass* and influence PIF.

### Domain 2: Characteristics of secondary traumatic stress

Recent conceptualizations of STS, which outline post-traumatic-like symptoms, build on Figley’s notion of stress arising from knowledge of another’s trauma. Marked by a conflation of others’ distress with one’s own and a blurring of professional and personal spaces, these PTSD-like features of STS are centered on destabilization within the Individual Ring, including invasive flashbacks, physiological hyperarousal, emotional numbing and concentration deficits. These disruptions may cascade into the Relational and Societal Rings when physicians withdraw from patients, colleagues and interpersonal relationships, or struggle to meet professional expectations. Table 5 details these features of STS.

**Table 5:** Characteristics of Secondary Traumatic Stress.

Characteristics	Description
Re-Experiencing Thoughts and Dreams [87–89]	PTSD-like intrusions disrupt meaning-making and self-reflection in the Individual Ring and distort the internal compass as moral reasoning becomes tainted with unresolved emotional residue.
Avoidance of Reminders or Specific Patients [87,90]	Avoidant behaviors compromise professional expectations and responsibilities within the Societal Ring, as well as tarnish patient-physician relationships embodied in the Relational Ring through reduced empathic engagement. This detachment may dull the internal compass’ sensitivity to patient needs.
Physiological Hyper‑Arousal [87,89]	Persistent hyper-arousal impairs concentration, reflection and judgment through heightened stress reactivity, destabilizing the Individual Ring. In turn, the internal compass becomes reactive rather reflective through assessment of perceived threats over moral deliberation.
Emotional Numbing / Blunted Affect [89,91]	Emotional numbing as protective behavior can strain the connection that underpins interpersonal and professional relationships, undermining the Relational Ring. This effect may also compromise resilience and disrupt the Individual Ring, alongside weakening the internal compass’ sensitivity to moral and emotional cues.
Concentration Deficits [90]	Concentration deficits impair self-efficacy in the Individual Ring and misalign with professional standards and expectation in the Societal Ring. This erosion of focus weakens the internal compass’ capacity for complex and ethical decision-making.
Social Withdrawal [83]	Social withdrawal jeopardizes interpersonal connections and collegial support, destabilizing the Relational Ring.
Sleep Disturbance & Nightmares [34,90,92]	Sleep disturbances disrupt emotional and cognitive balance within the Individual Ring, impairing self-reflection and resilience. This fatigue blunts the internal compass, fostering impulsive responses

### Domain 3: Characteristics of vicarious trauma

While VT and STS share overlapping features akin to post-traumatic stress disorder (PTSD), VT is distinguished by its emphasis on enduring cognitive transformations. These include alterations in physicians’ worldviews surrounding safety, trust and justice, brought about by repeated exposure to suffering and traumatized patients and families [93]. Such changes reflect deeper disruptions in underlying belief systems, extending beyond the immediate emotional and physiological responses characteristic of STS. Table 6 summarizes these current attributes of VT.

**Table 6:** Characteristics of Vicarious Trauma.

Characteristics	Description
Intrusive Images / Flashbacks [78,94]	Flashbacks intrude upon the capacity to self-reflect, disrupt emotional regulation and diminish resilience, unsettling the Individual Ring and clouding the moral clarity of the internal compass.
Alteration of Worldview [87,93,95–98]	Outlooks on trust, safety and meaning may be rewired following exposure to others’ trauma, reshaping the Individual Ring and the guiding values and moral orientation that frame the internal compass.
Persistent Negative Affect [61,99]	Enduring emotional storms destabilize self-control and emotional regulation that underpins the Individual Ring. This negative affect colors moral interpretation and impairs the internal compass.
Avoidance / Emotional Numbing [61,76,100]	Avoidance or emotional numbing as protective behavior can strain the connection that underpins interpersonal and professional relationships, undermining the Relational Ring. This effect may also compromise resilience and disrupt the Individual Ring, alongside weakening the internal compass’ sensitivity to moral and emotional cues.
Relationship Shifts (Isolation/ Over-Identification) [87,93,97,98]	Relationship shifts place strain on the Relational Ring. Withdrawal reduces social grounding while overidentification clouds personal and professional boundaries.
Hyper-Arousal & Physiological Vigilance [101]	Persistent hyper-arousal impairs concentration, reflection and judgment through heightened stress reactivity, destabilizing the Individual Ring. In turn, the internal compass becomes reactive rather reflective through the assessment of perceived threats instead of moral deliberation.
Neuro‑Reciprocity / Mirror‑Neuron Resonance [83,101]	Heightened empathic mirroring of others’ trauma erodes the emotional boundaries within the Relational Ring and results in empathic overload in the Individual Ring. This conflation of others’ trauma with the self weakens the internal compass.

### Domain 4: Predisposing and protective factors

Presentations of CF, VT and STS are shaped by a range of individual and environmental risk factors. The differing strength and interaction of these factors reveals the fluidity of CF, VT and STS that may account for physicians’ shifting coping abilities and resilience.

These insights are key, particularly in high-intensity specialties such as oncology [102], palliative medicine [61,103] and emergency medicine [100,104], where physicians face recurrent, concurrent and prolonged patient suffering [99]. The interplay of individual and environmental factors also accounts for the varying effects of high patient volumes [76,100,104–106], long working hours [104,107] and work-life imbalance [83,99,101,105,108–110] on manifestations of CF, VT and STS.

Yet, there are also protective factors that can mitigate these experiences. These include sustaining professional fulfilment in caring for seriously ill patients—even in high pressure work environments [83,110–113]—healthy coping mechanisms and strong family support [77,101,105,114]. Access to timely and personalized counselling, as well as peer mentorship [76,77,79,83,96,97,100,112,115–119] also serve as buffers against CF, STS and VT.

The evolving nature of individual factors—such as accumulated experience, growing competence, reflective capacity, and reframing and meaning-making skills—coupled with changing settings, workloads and available support, further contribute to intra-individual variability support. Likewise, shifts in psycho-emotional states and changes in social and family circumstances influence how physicians experience and respond to CF, STS and VT. Table 7 marks these risk and protective factors associated with CF, STS and VT.

**Table 7:** Risk and Protective Factors Associated with CF, STS and VT.

Risk Factors	Protective Factors
**Environmental Factors**	
**Structural Stressors** • Fast-paced, high-pressure work environments [96,100,104,107,115,120] • Frequent exposure to death and medical trauma [34,82,83,91,102,105–109,112,116,119,121–126] • High patient volumes [76,100,104–106] • Insufficient organizational or supervisory support [99,101,105,106,108,117,127] • Long working hours and night on-calls [104,107,109,120,124] • Poor workplace safety [76,93] • Physically demanding work [104] • Understaffed workplaces [124] • Physician having to act against his own beliefs [100,113,128,129] **High-Intensity Specialties** • Critical care [117,129–131] • Emergency medicine [100,132–134] • Infectious diseases [135,136] • Oncology [61,85,102,137] • Palliative medicine [61,95,97,103] • Psychiatry [91,138–140] **Exposure Risks** • Close, prolonged relationships with dying patients [105,107–109,119,123–125,141] • Managing complex cases lacking a definitive diagnosis [76,100,105] • Engaging challenging patient interactions [104,108,142] • Caring for patients who have experienced trauma [91,99,101] • Caring for patients with intractable pain [111] • Caring for patients facing social disparities [125]	**Organizational and Interpersonal Supports** • Access to institutional support [76,77,115,116,143] • Access to mentors [76] • Adequate downtime after traumatic or intense work periods [100] • Feeling valued and supported by supervisors [76,96,114] • Good teamwork [115] • Higher employment protection [112] • Manageable workload [79,109] • Mutual interdisciplinary respect [76] • Support from co-workers [114,141] • Organizational training and education [76,83,143] • Working in fields offering practical treatment options [93] • Family and peer support [105,114] • Existential or spiritual support [135] • Not working on the frontlines [144] • Shorter shift lengths [76]
**Individual Factors**	
**Intrapersonal and Psychological Vulnerabilities** • Alexithymia or apathy [96,119,126] • Emotional volatility [90] • Introversion [90] • High empathy and relatability to patients [34,79,96,111,116,123,145] • Low levels of resilience [90] • Low self-esteem or harsh self-criticism [108,116] • Maladaptive coping strategies [79,81,97,118] • Experiencing burnout [102,129] • Personal history of trauma [99,105,108,130,146,147] • Overestimation of own abilities [99,105] • Perfectionism [109,111] • Poor team player [111] • Heightened sensitivity to negative feedback [109] • Psychological comorbidities [83,94,102,106,112,119,127,129,147,148] **Demographic and Situational Vulnerabilities** • Being married or having young children [124,127,149] • Early career physicians [99,105,108,109,111,112,115,127,135] • Female gender [124,149] • Further education beyond medical school [108,131] • Sleeping fewer than six hours per nightly [117]	**Intrapersonal and Psychological Strengths** • Ability to reframe thoughts [101] • Ability to regulate emotions [113,119] • Adequate self-care practices [6,100,114] • Engaging in physical activity [100,115] • Compassion satisfaction [76,81,83,96,99,102,113,119,141] • Empathy [116,119,148] • High emotional stability [90,98] • Use of humor [114] • Internal locus of control [113] • Mindfulness [150] • Strong resilience [6,76,83,93,98,99,101,151] • High self-awareness [105,119,141] • Self-compassion [100,116] • Enjoyment of clinical practice [83,112] • Finding meaning in work [93] • Fulfilment in helping patients [113] • Higher education level [76,136] • Clinical experience [76,129]

### Domain 5: Interventions

Current efforts to mitigate CF, STS and VT overlap and revolve around:

1. Reducing work stress and enhancing the work environment [6,77,87,94,101,106–108,111,126,128,152–154].
2. Enhancing the work culture to prioritize staff well-being and support [100,101,106,153], including peer and mentoring support [16].
3. Providing personalized, appropriate, specific, timely and accessible support, including
  a. debriefs after a patient’s death [106,111,152]
  b. access to cognitive behavioral therapy [106,136]
  c. emotional regulation strategies [82,110,126,145]
  d. communication training and counseling [106,126,155]
  e. guided reflections on patient death, patient trauma and work stressors [103]
  f. improving coping and resilience skills [79,103,111,126]
4. Pre-emptive strategies, including:
  a. periodic evaluations of emotional variables and CF/STS/VT risk assessment such as ProQOL, Beck Depression Inventory and Balanced Emotional Empathy Scale [106,123,146]
  b. regular physician well-being and satisfaction screening [105]
  c. raising awareness and psychoeducation on CF, VT and STS and their identifiable symptoms [6,93,100,105,106,110,116,126,154].

## Discussion

This mSEBA-guided, AI-assisted review advances a clinically relevant and physician-specific conceptualization of CF that encapsulates its intersections with VT and STS. Building on growing evidence, this review highlights the increasingly complex ways CF, STS and VT are described and understood. In doing so, it reveals the shared mechanisms of overidentification, boundary breaches and transference through which CF, STS and VT unfold—suggesting that these constructs are better understood not as discrete entities, but as a continuum of emotional, empathic and trauma-related strain.

**Fig 2:**
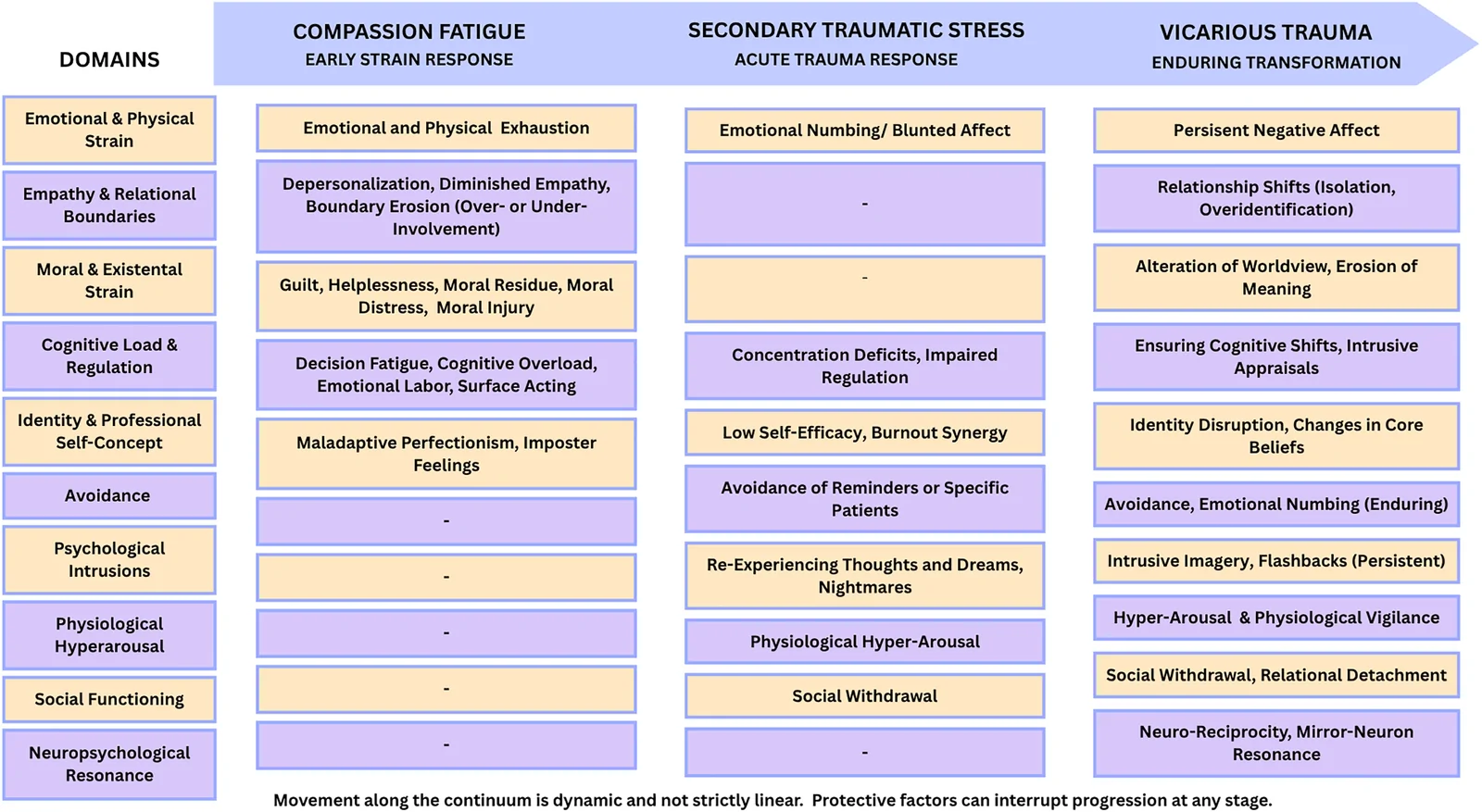
Compassion Fatigue-Secondary Traumatic Stress-Vicarious Trauma Continuum.

Fig 2 presents this continuum visually by clustering shared and differentiating features. Understanding CF, VT and STS as a continuum clarifies how their shared mechanisms exert cumulative and progressively intensifying effects on PIF. Across CF, STS and VT, physicians commonly experience moral (guilt, helplessness, moral injury), cognitive (decision-fatigue, cognitive overload, surface acting), relational (depersonalization, boundary erosion) and identity-related (perfectionism, imposter feelings) disruptions that destabilize the physician’s *internal compass* guiding emotional regulation, professional conduct and meaning-making. When these stabilizing mechanisms are weakened, the physician becomes increasingly vulnerable to the intrusive, avoidant and hyper-arousal symptoms characteristic of STS and the deeper worldview or identity shifts associated with VT.

Within this continuum, the distinction between STS and VT becomes clearer. Marked by their differing emphases, STS is primarily concerned with post-traumatic stress symptomatology, whereas VT centers on more enduring cognitive transformations, including alterations in worldview and core belief systems. Concurrently, depressive symptoms, social isolation, avoidant behavior and PTSD-like responses associated with STS and CF can progressively erode professional fulfilment and emotional-cognitive acumen and can, over time, contribute to the more entrenched worldview changes seen in VT. The recurring presence of MD within CF also suggests a progressive pathway, wherein MD and CF may intensify trauma-related strain, increasing susceptibility to STS and, ultimately, VT [34,116,135].

Understanding CF, VT and STS as a continuum also provides a more cohesive account of their cumulative effects on PIF. Given the dynamic, relational and context-dependent nature of PIF [45,47], each point along the continuum disrupts different layers of identity development. Early perturbations within the Individual Ring (e.g., emotional exhaustion, diminished confidence) signal CF’s erosion of foundational self-evaluative processes. Unaddressed, these disruptions may progress to encroachments in the Relational Ring denoted by STS-driven avoidance and withdrawal. At the furthest end of the continuum, VT disrupts meaning-making structures housed in the Innate and Societal Rings, reshaping beliefs about the world, one’s role and what it means to be a good doctor.

In this way, the CF–STS–VT continuum not only reflects escalating trauma-related stress but also delineates a trajectory of progressive disruption to PIF, wherein *the internal compass* becomes increasingly compromised. Understanding this trajectory is essential for designing effective interventions that refine the *internal compass*, restore identity coherence and promote resilience in physicians.

### Practical implications

These insights have significant ramifications upon clinical training and practice. To begin, there must be greater attention paid to the structure, culture and support systems within training practices. General provision of support for physicians in specialties characterized by high exposure to patient suffering, heavy patient volumes, long working hours and work-life imbalance—such as oncology [61,85,102,137], palliative care [61,95,97,103], emergency medicine [100,132–134], critical care [76,117,129–131], infectious diseases [135,136] and psychiatry [91,138–140]—is insufficient if limited to improvements in the workplace and learning environments alone. Rather, such support should also extend to guidance in personal domains, including marriage and parenthood [117,124,127,131,149], alongside relationships with family, colleagues and patients [76,77,115,116,143].

Training programs should therefore recognize the value of general support measures whilst acknowledging the evolving needs of physicians. This necessitates a proactive, rather than reactive, approach. Education programs should increase awareness of CF, VT and STS; enhance training in reflective cycles; cultivate resilience, self-awareness and adaptive coping strategies. In addition, regular assessments, check-ins, debriefings and case discussions ought to attenuate the risk of snowballing issues and improve emotional regulation.

These recommendations are summarized below. Host organizations should:

1. Foster supportive workplace cultures [100,101,106,153] that normalize help-seeking behavior and value psychological safety.
2. Improve working conditions, including reducing patient volumes [76,100,104–107] and limiting excessive working hours [109,120,124,139,156–158].
3. Embed educational initiatives to promote self-awareness [6,79,93,100,101,105,106,108,110,116,126,131,154,159], emotional regulation, work-life balance and self-care [6,82,94,100,103,105,106,111,131,155,160–162] to effectively navigate work-related stressors [79,103,111,126].
4. Implement proactive assessment-driven policing [34,90,91,96,110,120,124,127,135,146,163,164] to identify and address the risk early.
5. Ensure access to confidential counseling services [87,165], mindfulness training and cognitive behavioral therapy [106,136].
6. Strengthen interpersonal and institutional supports through family, collegial and leadership engagement [94,101,111,146].

### Future research directions

There remains a need to prioritize high-quality, longitudinal studies evaluating the long-term impact of the evolving concept of CF, VT and STS. More personalized interventions, particularly beyond North American and European contexts, are also required to contend with sociocultural influences, such as Confucian-based beliefs that shape many Asian clinical environments. These insights will allow for better development of comprehensive assessment tools that capture the emotional and existential dimensions of well-being essential to advancing this field.

### Limitations

This review has several limitations. One, it does not account for cultural or country-specific factors that may influence physicians’ roles, coping mechanisms and experiences with CF, STS and VT. Two, the confinement of literature searches to only English-language publications has likely contributed to the predominance of studies from North America and Europe, potentially limiting the generalizability of our findings. Three, the paucity of published intervention studies with negative results may have introduced bias. Four, the lack of inter-rater reliability, given that this study formed part of a research mentoring program, raises concerns on the trustworthiness of this review. Five, the focus on medical specialties that exclude surgical and pediatric disciplines, limits the exploration of plausible differences in the conceptualization or management of CF, STS and VT. Six, although recommendations are offered to host organizations, many suggested assessments and interventions have not been fully evaluated. Seven, few studies have evaluated the efficacy of the various interventions advanced, even though they have been shown promise in other contexts. Eight, our use of mSEBA’s AI models, though used in tandem with traditional manual approaches, may introduce bias.

## Conclusion

CF, VT and STS exert profound effects across all dimensions of a physician’s professional and personal lives, with downstream implications for patient care, team dynamics and workforce sustainability. As this SSR highlights, the CF, STS and VT extend beyond emotional and physical exhaustion following empathic care to encompass moral, cognitive, relational and identity-related dimensions that shape belief systems and PIF. Future efforts should thus focus on the active evaluation of these phenomena within the clinical setting and invest in the development of an effective assessment tool to better capture and address this expanded notion of the CF, STS and VT.

## Supporting information

S1 Fig Krishna-Pisupati Model. (TIF)

S2 Fig Modified Systematic Evidence-Based Approach. (TIF)

S1 Appendix PRISMA-ScR Checklist. (PDF)

S2 Appendix Final List of Included Articles. (DOCX)
