# Supplementary material for: Understanding the cost of caring in medicine through personhood: A systematic scoping review of compassion fatigue, vicarious trauma and secondary traumatic stress
Source: PLoS One. 2026 Sep 10;21(9):e0357726. doi: 10.1371/journal.pone.0357726 (PMC13561344; doi:10.1371/journal.pone.0357726)
Supplement: Supplementary file 2 [file pone.0357726.s004.docx]

**S2 Appendix. Final List of Included Articles**

| **No.** | **Author** | **Article Year** | **Article Title** | **Study Type** | **Study Quality Assessment Score i.e. MERSQI** | **Qualitative Research Reporting Checklist Compliance Score i.e. COREQ** | **Study Aim** | **Methodology** | **Key Findings** | **Proposed Solutions/Conclusions** |
| --- | --- | --- | --- | --- | --- | --- | --- | --- | --- | --- |
| 1 | Laor- Maayany | 2020 | Compassion fatigue among oncologists: the role of grief, sense of failure, and exposure to suffering and death | Cross- sectional study | 12.5 | NA | Oncologists grapple with distinctive job  characteristics that elevate their susceptibility to compassion fatigue, encompassing burnout and secondary traumatic stress. This condition may diminish their ability and inclination to empathize with others' suffering.  Simultaneously, oncologists may also encounter compassion satisfaction, signifying the positive aspects of caregiving. This study delves into the connections between compassion fatigue, compassion satisfaction, oncologists' experience of grief, and their sense of failure, extending beyond their reported exposure to suffering and death. | A total of 74 oncologists participated in the study, completing self-administered questionnaires that assessed compassion fatigue, compassion satisfaction, grief, exposure to suffering and death, and their sense of failure. | The surveyed oncologists revealed that they confront the loss of around 50% of their patients, who often endure intense emotional and physical pain. The reported levels of compassion fatigue and grief were high, while the sense of failure was moderate. Surprisingly, the findings indicated no significant association between exposure to suffering and death and compassion fatigue or satisfaction. However, both grief and the sense of failure emerged as predictors for both dimensions of compassion fatigue: secondary traumatic stress and burnout. | The outcomes underscore the significance of oncologists' personal encounters with grief and a sense of failure, surpassing their accounts of exposure to suffering and death, regarding their compassion fatigue levels. The implications of these findings stress the necessity for interventions tailored to oncologists, enabling them to recognize, process, and overcome negative experiences related to failure and grief. |
| 2 | Şahan, S | 2022 | Investigating the compassion fatigue in emergency department physicians and nurses | Cross- Sectional Study | 13 | NA | This study aims to investigate levels of compassion fatigue in doctors and nurses in the Emergency Departments in Turkey. | This study was carried out as a descriptive study to assess compassion fatigue among physicians and nurses employed in emergency departments in Turkey. | This research found that the levels of compassion fatigue in 67% of the doctors and 73.5% of the nurses working in the emergency departments were higher than moderate levels. There was a notable distinction between the levels of compassion fatigue in nurses and doctors.  Additionally, for every 3 doctors, 2 doctors experienced levels of compassion fatigue which exceeded average levels. | The study findings revealed that physicians and  nurses in the emergency department experienced compassion fatigue above average levels.  Emergency department managers should implement timely and effective interventions to alleviate compassion fatigue among physicians and nurses. Such interventions may include training programs focused on managing stress and education on coping methods for those working in the emergency department. |
| 3 | Marcin Rzeszutek | 2016 | SECONDARY TRAUMATIC STRESS DISORDER SYMPTOMS IN A SAMPLE OF THERAPISTS AND PSYCHIATRISTS  WORKING WITH PEOPLE AFTER TRAUMATIC EVENTS | Cross- sectional study | 14 | NA | The objective of this study was to compare the level of symptoms related to secondary traumatic stress disorder among trauma therapists and psychiatrists, considering demographic variables such as age and work- related factors including years of experience, the average number of trauma patients attended to in the past 12 months, and the presence of clinical supervision. | The research comprised 160 participants, consisting  of 80 therapists and 80 psychiatrists, all of whom were actively involved in working with trauma victims. To gauge the extent of symptoms related to secondary traumatic stress disorder, participants completed the PTSD Questionnaire: Factorial Version. Additionally, the Berlin Social Support Scales were utilized to evaluate the level of social support available to them. Participants also supplied demographic details and information related to their work. | The outcomes of our study unveiled elevated rates of symptoms related to secondary traumatic stress disorder (STSD) among psychiatrists in comparison to therapists. Additionally, notable differences were observed in terms of age, years of experience, and the actual support received between the two groups of professionals. | Mental health specialists should be mindful of the potential adverse effects associated with working with trauma victims, particularly in the manifestation of symptoms related to secondary traumatic stress disorder. |
| 4 | Jackson- Koku, Gordon | 2021 | An exploratory study of the role of emotion regulation and emotional intelligence in compassion satisfaction and fatigue among doctors and nurses | Cross- sectional study | 12.5 | NA | The study aims to investigate the relationships among emotion management strategies (ER), emotional intelligence (EI), compassion satisfaction (CS), and fatigue (CF) in doctors and nurses working at a major National Health Service (NHS) Hospital in the United Kingdom. Additionally, the goal is to pinpoint the variable or variables that can predict levels of compassion satisfaction and fatigue in both doctors and nurses. | A total of 487 participants responded to questionnaires evaluating six potential explanatory factors for compassion satisfaction and fatigue.  These factors included (i) intrapersonal emotional intelligence (EI), (ii) interpersonal EI, (iii) cognitive reappraisal, (iv) cognitive fusion, (v) expressive suppression, and (vi) emotional contagion.  Additionally, two open-ended questions were included to capture insights into the positive and negative aspects of doctors and nurses' roles that might contribute to emotional stress and potentially impact compassion satisfaction (CS) and fatigue (CF). The data analysis involved descriptive statistics, correlations, regression, and mediation analysis. Content analysis was employed to analyze responses to the open-ended questions. | Cognitive reappraisal and intrapersonal emotional  intelligence (EI) were identified as predictors, with intrapersonal EI being the stronger predictor.  Intrapersonal EI demonstrated a positive prediction for compassion satisfaction (CS) and a negative prediction for fatigue (CF), surpassing the predictive impact of cognitive reappraisal on CS and CF. Furthermore, intrapersonal EI played a partial mediating role in the relationships between cognitive reappraisal and both CS and CF. Content analysis of open-ended questionnaire responses highlighted that 54% of participants found enjoyment in aspects related to direct patient care/ contact, while 60% expressed dissatisfaction due to workplace stress, bureaucracy, lack of support and resources, and workplace politics. This qualitative insight supports the quantitative findings, emphasizing the significance of developing intrapersonal emotional intelligence skills to enhance reappraisal strategies crucial for managing negative emotions stemming from workplace stressors, ultimately influencing CS and CF. | Addressing intrapersonal emotional intelligence (EI) emerges as a critical focal point for potential psychological interventions designed to improve compassion satisfaction (CS) and mitigate fatigue (CF) among doctors and nurses in clinical settings. |
| 5 | Franco | 2021 | Alexithymia and professional quality of life in radiation oncology: The moderator effect of the professional profile | Cross- sectional study | 13.5 | NA | Cancer care poses significant challenges, and  the presence of alexithymia, a personality trait marked by difficulties in recognizing and expressing emotions, an externally-focused thinking style, and a lack of imagination, is closely linked to higher levels of secondary traumatic stress (STS) and burnout, as well as lower levels of compassion satisfaction among medical professionals in radiation oncology.  This study aims to investigate variations in professional quality of life (QoL) and its connection to alexithymia within different professions in the multidisciplinary field, namely radiation/clinical oncologists (RO), medical physicists (MP), and radiation  therapists (RTT). | The study used an online questionnaire, collecting 1500 responses from May to October 2018. The assessment of alexithymia utilized the Toronto Alexithymia Scale (TAS-20), while professional quality of life (QoL) was gauged using the Professional Quality of Life Scale (ProQoL) version  5. To compare the groups of radiation/clinical oncologists (RO), radiation therapists (RTT), and medical physicists (MP), ANOVA or MANOVA was conducted, followed by Bonferroni-corrected ANOVAs for continuous variables and Pearson's chi- square test for categorical variables. The effect size was determined through the calculation of partial eta- squared (η²). | The correlation between alexithymia and secondary traumatic stress (STS) was influenced by the profession, with radiation oncologists (RO) showing a higher risk compared to medical physicists (MP) and radiation therapists (RTT). The study highlights a noteworthy prevalence of decreased well-being at work across professional categories, including medical physicists, despite their technical profile and limited patient interaction. In contrast to medical physicists, radiation oncologists exhibited a significantly higher percentage of respondents with elevated levels and a lower percentage with low levels of secondary traumatic stress symptoms. | The study underscores the significance of alexithymia as a contributing factor to the decline in professional quality of life (QoL) among professionals in radiation oncology. The impact is more pronounced on radiation oncologists (ROs) with alexithymia compared to medical physicists (MPs) and radiation therapists (RTTs), particularly due to their indirect exposure to patients in distress. These findings suggest a need to address these observations in professional education with the aim of enhancing the overall quality of life for healthcare personnel in the field. |
| 6 | Hayuni | 2019 | Between empathy and grief: The mediating effect of compassion fatigue among oncologists | Cross- sectional study | 14 | NA | Oncologists frequently encounter suffering, loss, and death, potentially eliciting grief reactions. While grieving for patients is a natural outcome of the close and enduring oncologist-patient bond, the empathy oncologists invest in their patients may make them susceptible to compassion fatigue (CF). This study aims to investigate how the two elements of CF—secondary traumatic stress (STS) and burnout—mediate the connection between empathy and grief in oncologists. | Participants comprised 71 Israeli oncologists, and the assessment tools included a demographic survey, the Texas Revised Inventory of Grief-Present, the Interpersonal Reactivity Index, and the Professional Quality of Life questionnaire. | Oncologists indicated moderate grief levels and  relatively high levels of both secondary traumatic stress (STS) and burnout. They also reported elevated levels of the three empathy components: perspective taking, empathic concern, and personal distress. Utilizing the PROCESS SPSS macro, the study found substantial support for the mediation model. The mediators, specifically STS and burnout, completely explained the relationship between two empathy components, perspective taking and personal distress, and the experience of grief. | Oncologists frequently undergo grief due to their patients, and the study revealed a connection between oncologists' grief and their empathy. This association was elucidated by their levels of compassion fatigue (CF). The current research signifies progress in acknowledging and comprehending the processes linked to oncologists' grief. |
| 7 | Ondrejková, N. | 2022 | Prevalence of compassion fatigue among helping professions and relationship to compassion for others, self-compassion and self- criticism | Cross- sectional study | 13 | NA | Professionals in helping roles face a  heightened risk of experiencing the negative consequences associated with helping, such as compassion fatigue. However, no study has yet presented a comprehensive overview of compassion fatigue and compared its prevalence across various helping professions. This study aims to investigate the prevalence of compassion fatigue among different helping professions and examine potential differences. Additionally, we seek to explore the relationships between compassion, self- compassion, self-criticism, and compassion fatigue. | 607 participants employed in helping professions were enrolled. Among 607 participants, 44 of them are doctors, the remaining are home nurses, teachers, police officers etc. Data collection utilized an online questionnaire battery to assess levels of compassion, self-compassion, self-criticism, compassion fatigue, and compassion satisfaction. | The study identified noteworthy variations in compassion fatigue levels across distinct helping professions. No substantial distinctions were observed in the levels of compassion and self- compassion between professionals with moderate versus low compassion fatigue scores.  Nevertheless, individuals with elevated compassion fatigue demonstrated higher self- criticism. Notably, self-criticism emerged as the most significant predictor of compassion fatigue. Furthermore, the impact of one's profession on compassion fatigue, mediated by self-criticism and self-compassion, was found to be significant. | The findings suggest the need for programs aimed at alleviating compassion fatigue among helping professionals. These programs should focus on imparting skills to effectively manage work time and workload, specifically the hours spent per week with clients or patients. Additionally, interventions should target fostering healthier inner dialogue by reducing self-critical tendencies and enhancing self- compassion. |
| 8 | Račić | 2019 | Compassion fatigue and compassion satisfaction among family physicians in the Republic of Srpska, Bosnia and Herzegovina | Cross- sectional study | 14 | NA | The study aimed to assess how family physicians in Bosnia and Herzegovina perceive their own levels of compassion fatigue and compassion satisfaction, as well as to outline factors that may contribute to these perceptions. | In this cross-sectional study, 120 family physicians participated. The assessment of compassion satisfaction, along with two components of compassion fatigue (secondary traumatic stress and burnout), was conducted using the Professional Quality of Life Compassion Satisfaction and Fatigue, Version 5 (ProQOL5). Additionally, chronic fatigue symptoms were evaluated using the Chalder Fatigue Scale. | A significant portion of family physicians  experienced moderate levels of compassion satisfaction (70%), burnout (75%), and secondary traumatic stress (55.8%). Family physicians with elevated levels of secondary traumatic stress tended to report chronic fatigue, longer length of service, and residency training. Higher risk of burnout was associated with chronic fatigue, residing in a rural environment, managing larger- sized practices, and having a higher number of patients with chronic diseases. | Family physicians at risk of developing compassion fatigue include those with extensive practices, extensive years of experience, a substantial number of chronically ill patients, and those experiencing chronic fatigue. A systematic investigation into the relationship between compassion fatigue and working conditions could serve as a valuable starting point for the formulation of preventive interventions. |
| 9 | Loolo | 2016 | Compassion fatigue and crisis workers' attitude to work | Cross- sectional study | 13.5 | NA | Mental health practitioners often face  challenging emotional reactions while working with traumatized clients. The strain of providing care in trauma-related settings can lead to a depletion of empathy and compassion, resulting in compassion fatigue—an apathetic and indifferent response to the suffering of others. This cross-sectional study, conducted in West Africa, explores the predictive relationships between compassion fatigue and work attitudes among primary care physicians. The study utilizes the etiological model of compassion fatigue and the constructivist self- development theory (CSDT) as a conceptual framework to understand how clinicians respond to trauma-related experiences in their clinical work. The primary research question investigates the extent to which the level of compassion fatigue predicts practitioners' attitudes toward their daily work activities in the clinical setting. | Information from 67 primary care physicians was gathered through validated surveys and analyzed employing a linear regression modeling method. | The findings revealed that the compassion fatigue of practitioners significantly predicted their work attitudes, as indicated by statistical significance. Additionally, the results affirmed that compassion satisfaction can moderate the impact of compassion fatigue in practitioners. | This study delivers valuable insights to primary care physicians regarding compassion fatigue and underscores the importance of effective self-care. The results can influence healthcare leaders in developing countries to implement training and welfare programs as strategies to enhance physicians' well-being. |
| 10 | El-bar | 2013 | Compassion fatigue, burnout and compassion satisfaction among family physicians in the Negev area  - a cross-sectional study | Cross sectional | 12.5 | NA | Healthcare professionals' experience of compassion fatigue has become a focal point in recent years. This phenomenon, alongside burnout, has been linked to reduced personal connections and substandard patient care.  Professionals in caregiving roles are consistently exposed to the risks associated with compassion fatigue (CF), burnout (BO), and diminished levels of compassion satisfaction (CS). Despite CF being studied among healthcare professionals, minimal literature examines its occurrence specifically among family physicians. This study aimed to gauge the prevalence and intensity of CF within family practitioners (FPs) in the Negev region of Israel. Additionally, it aimed to explore the correlations between CF, BO, and CS, and how these relate to various socio- demographic factors and work-related characteristics. | Between July 2007 and April 2008, 194 family physicians from Clalit Health Services clinics in the Negev completed anonymous Compassion Satisfaction and Fatigue Test questionnaires (CSFT) to evaluate CF, BO, and CS. The study assessed correlations among CF, BO, and CS. Multivariable logistic regression models, employing backward elimination, were constructed to analyze the data. | Among respondents, 46.1% showed extremely high or high levels of CF, while 21.1% exhibited low CS and 9.4% had high BO. The study discovered robust correlations between BO and CF, and between BO and CS. However, no correlation emerged between CS and CF. In the logistic regression model, former immigration to Israel was the sole factor linked to a notably increased risk of CF. Female gender, a history of personal trauma, and lacking academic affiliation were associated with an increased risk of BO. Conversely, higher CS was associated with holding management positions and teaching residents. | Family physicians in the Negev face a notable risk of CF, which could lead to patient dissatisfaction, compromised safety, and increased medical errors. We suggest establishing an educational and early intervention program aimed at family physicians and other healthcare professionals to address CF. These programs should focus on training individuals to facilitate physician well-being and build resilience. Additionally, analyzing factors contributing to higher CF and organizational elements is recommended. Policy-wise, integrating these programs into mandatory risk management continuing medical education is advisable. |
| 11 | Highfield, J. | 2020 | Professional Quality of Life in intensive care medicine: The 2018 Faculty of Intensive Care Medicine Workforce survey | Cross sectional | 11 | NA | The study aimed to explore the equilibrium  between emotional rewards and stress among UK critical care consultants affiliated with the Faculty of Intensive Care Medicine. This investigation was prompted by the perception that critical care, being emotionally taxing, potentially poses a higher risk of burnout among medical staff compared to other specialties. | The methodology involved executing a Wellbeing survey within the Faculty of Intensive Care Medicine consultant membership, employing the Professional Quality of Life (Pro-QOL) survey tool. This survey was integrated into the Annual Workforce Census process. | The findings revealed a moderate risk of burnout and secondary traumatic stress among critical care consultants, offset by a moderate level of compassion satisfaction. No significant associations were identified based on age, gender, or the size of the critical care unit they worked in. | It's suggested that ongoing monitoring of this consultant group could provide insight into burnout risk factors and help develop strategies to address them while enhancing the positive aspects of working in critical care medicine. |
| 12 | Carmassi, C. | 2022 | Mental Health Symptoms among General Practitioners Facing the Acute Phase of the COVID-19 Pandemic: Detecting Different Reaction Groups | Cross- Sectional Study | 14 | NA | In the initial wave of the COVID-19 pandemic  in 2020, general practitioners (GPs) served as the forefront of primary healthcare and were thus highly susceptible to pandemic-related risks, leading to an increased likelihood of experiencing various mental health symptoms. However, there is limited data available which explores factors that are linked to a more adverse mental health outcome. This study aims to find out more about the impact of the first COVID-19 national lockdown on the mental health of GPs in Italy. | This research studied the effects of the first COVID-19 national lockdown on139 GPs in Italy.  This study explored the extent of depressive, anxiety, and post-traumatic stress symptoms amongst these participants, the effects of these symptoms on their quality of life and assessed how it affected their function. | A cluster analysis revealed the presence of three groups characterized by mild (44.6%), moderate (35.3%), and severe psychopathological burden (20.1%). Being youngr, having lesser years of experience as general practitioners (GPs), employment in areas with high COVID-19 incidence, having a relative susceptible to medical complications from COVID-19, as well as experiencing profound impairment to overall function, burnout, and secondary traumatic stress was associated with an increase in severity of symptoms. | The current results indicated that general practitioners (GPs), who had no choice but to carry out their professional duties even in exceedingly stressful situations amidst the COVID-19 pandemic, were very susceptible to experiencing mental health issues and a worsened quality of life. |
| 13 | Kindermann, D. et al. | 2018 | Experiences and psychological strain in volunteer medical doctors providing medical visual examination for asylum seekers in a reception center in Germany-a qualitative interview study | Qualitative research study | NA | 13 | The study aimed to analyze the motivations, experiences, and psychological strain faced by volunteer medical doctors performing medical visual examinations (MVEs) for asylum seekers at a reception center in Germany. Specific attention was paid to the emergence of secondary traumatic stress (STS), vicarious traumatization (VT), and the need for psychosocial support. | Semistandardized interviews were conducted with 18 medical doctors after they had performed MVE. Interview recordings were transcribed and subsequently underwent qualitative thematic analysis. Finally, thematic clusters were identified. The analysis revealed 512 relevant single codes, from which three main categories were derived. | Motivations: Physicians cited occupational and moral duty, professional curiosity, and personal or familial experiences of displacement as motivations for volunteering.  Experiences: Some participants reported positive feelings from interactions with asylum seekers. Others described the atmosphere as chaotic, depressing, or bureaucratic. Refugee narratives, particularly involving children, elicited strong emotional reactions.  Challenges included language barriers, limited scope of medical assessments, and ethical dilemmas.  Psychological Strain: Symptoms indicative of VT were observed in some participants, including cognitive schema changes. Most respondents reflected on their personal beliefs and moral concepts after the experience. The need for psychosocial support was highlighted, though opinions on its necessity varied. | The study underscores the psychological challenges of volunteering in high-stress environments like refugee reception centers. While intrinsic motivations drive many physicians, the emotional burden of exposure to traumatic narratives and systemic challenges requires better support mechanisms, such as supervision and targeted interventions to prevent VT and promote resilience. |
| 14 | Iyamuremy e | 2015 | The development of a model for dealing with secondary traumatic stress in mental health workers in Rwanda | Pre post studies | 13.5 | 8 | Mental health workers are constantly exposed  to the stories of suffering of their clients, putting them at risk of developing secondary traumatic stress. In Rwanda, mental health workers are at especially higher risk because of the 1994 genocide, which made them not only witnesses but also victims of the violence. This study aims to form a framework for management of the impacts of secondary traumatic stress in mental health workers in Rwanda. | Researchers interviewed mental health professionals, including nurses, doctors, psychologists, trauma counsellors, and social workers and measured secondary traumatic stress levels in them using Trauma Attachment Belief Scale. | Researchers formulated a framework, Intervention Model to Manage Secondary Traumatic Stress (IMMSTS). It encompasses a range of strategies, including preventive, evaluative, and curative measures designed to address secondary traumatic stress (STS) in mental health workers operating in Rwanda. These strategies are tailored to operate at multiple levels, spanning the individual, social, and organizational dimensions. | The Intervention Model to Manage Secondary Traumatic Stress can be used to effectively address STS. |
| 15 | Ahmed, F. | 2022 | Examining the Constructs of Burnout, Compassion Fatigue, Secondary Traumatic Stress in Physicians Using Factor Analyses | Cross- Sectional Study | 12.5 | NA | There is extensive literature which studies the negative emotional experiences faced by healthcare professionals. These negative emotional experiences are often characterized using various terms, such as burnout, secondary traumatic stress (STS), and compassion fatigue (CF). This study explores the conflicting models of STS, CF, and burnout constructs specifically in physicians. | Researchers distributed surveys to physicians  holding active Rhode Island medical licenses, resulting in 375 complete responses.. These surveys incorporated sections to measure levels of Secondary traumatic stress (STS), compassion fatigue (CF), and burnout amongst respondents. In analysis, Confirmatory Factor Analysis (CFA) was employed to assess the discriminant validity of the three constructs as well as to test 5 a priori (1-, 2-, and 3-factor) theoretical models. In the event where CFA did not support any current model, the researchers planned to use Exploratory Factor Analysis (EFA) to evaluate the underlying factor structure. | This study showed that with the use of the  Confirmatory Factor Analysis (CFA), there was a failure to show adequate model fit for the five a priori models for burnout, compassion fatigue (CF), and secondary traumatic stress (STS) (Standardized Root Mean Square Residual >0.10, Tucker-Lewis Index <0.90). Employing Exploratory Factor Analysis (EFA) with parallel analysis revealed four factors underlying the three measures of burnout, STS, and CF. These factors are (1) depressive mood; (2) primary traumatic stress-like symptoms; (3) responses to patients’ trauma; and (4) sleep disturbances and these factors account for 54.3% of variance. | This study showed that measures of Burnout, CF, and STS did not align with their theoretical factor structures, despite there being extensive amounts of discussion on these. In future, other research studies may look into alternative constructs and measures to describe the negative emotional effects experienced by physicians. |
| 16 | Hamilton | 2016 | Compassion fatigue in emergency medicine: The cost of caring | Grey Literature | NA | NA | This article examines the prevalence and impact of compassion fatigue (CF) among emergency medicine (EM) professionals. It highlights the emotional toll of continuous exposure to trauma and distress in the emergency department (ED) and underscores the importance of recognizing and addressing CF to maintain clinician well-being and ensure quality patient care | NA | Definition of Compassion Fatigue: CF is described as the emotional and physical exhaustion that EM professionals may experience due to the constant exposure to patients' trauma and suffering. This condition can lead to a diminished capacity for empathy and a decline in job performance.  Prevalence in Emergency Medicine: The article notes that EM practitioners are particularly susceptible to CF because of the high-stress environment, frequent encounters with critical incidents, and the need for rapid decision-making under pressure.  Impact on Healthcare Providers: CF can result in decreased job satisfaction, increased absenteeism, and a higher likelihood of errors in patient care. It may also contribute to burnout, further exacerbating the challenges faced by EM professionals.  Strategies for Mitigation: The authors suggest several approaches to mitigate CF, including:  Self-Care: Encouraging EM professionals to engage in regular self-care practices to manage stress and prevent burnout.  Organizational Interventions: Promoting a supportive work environment that acknowledges the challenges of EM and fosters open communication. | Given the growing presence of compassion fatigue in Emergency Departments (EDs), it's crucial to identify its symptoms and understand the possible repercussions of not addressing this issue, both for individuals and patient care within healthcare systems. As frontline healthcare providers, understanding this potentially damaging phenomenon is essential. There's a pressing need for more data specifically focused on Emergency Medicine (EM) within Australasia to evaluate the impact of compassion fatigue in our settings. This will enable the implementation of timely interventions to alleviate its effects. |
| 17 | Crowe, L. | 2016 | Identifying the risk of compassion fatigue, improving compassion satisfaction and building resilience in emergency medicine | Grey Literature | NA | NA | In the field of Emergency Medicine, healthcare  professionals are at risk of compassion fatigue (CF). This risk is attributed to various factors such as the fast-paced nature of the work, high expectations, the volume of patients, and the acuity of cases that staff encounter on a daily basis. | NA | Compassion Fatigue (CF): CF is identified as a significant risk for health professionals working in EM due to the high-paced environment, heavy patient loads, and the emotional demands of providing care. The article highlights that CF can lead to emotional exhaustion, reduced empathy, and decreased job satisfaction.  Compassion Satisfaction (CS): CS refers to the fulfillment derived from helping others and the positive feelings associated with being an effective caregiver. The author suggests that increasing CS can counterbalance the effects of CF and contribute to overall well-being.  Resilience Building: The article proposes several strategies to build resilience among EM professionals, including:  Self-awareness: Encouraging practitioners to reflect on their emotional responses and recognize signs of CF early.  Professional Support: Promoting a supportive work environment where colleagues can share experiences and provide mutual support.  Work-Life Balance: Emphasizing the importance of maintaining a healthy balance between professional responsibilities and personal life to prevent burnout.  Continuing Education: Encouraging ongoing learning and professional development to enhance skills and job satisfaction. | The nature of Emergency Medicine healthcare professionals' work demands a constant connection with the emotional and often distressing experiences of those they serve, contributing to the persistent risk of compassion fatigue among emergency healthcare professionals |
| 18 | Fitzpatrick, M. et al. | 2020 | Emotional exhaustion among regional doctors in training and the application of international guidelines on sustainable employability management for organisations | Cross-sectional study | 10 | NA | The first objective of this study was to assess the associations between individual, community and hospital factors with emotional exhaustion (EE) among rural Australian doctors in training (DITs); the second objective was to apply criteria from an international standard that measures sustainable employability in organisations. | A cross-sectional study of 70 DITs was conducted. Odds ratios (ORs) with 95% confidence intervals (CIs) of EE across individual, community and organisational factors were calculated | DITs with poor or moderate ability to cope with mental work demands had higher odds of being emotionally exhausted (OR 8.273; 95% CI 1.693-40.423), as did DITs with sleep disturbance (OR 6.187; 95% CI 1.721-22.242). Higher psychological distress (OR 1.701; 95% CI 1.286-2.249), more hours worked on-call (OR 1.052; 95% CI 1.011-1.094) and increased presenteeism (OR 1.285; 95% CI 1.049-1.576) were associated with being emotionally exhausted. Those who felt ambivalent or unsatisfied with hospital support networks had threefold higher odds of EE (OR 3.323; 95%CI 1.191-9.273). All metrics associated with the International Organisation for Standardisation (ISO) sustainable employability guidelines were significantly associated with EE; DITs who thought the hospital did not promote physical (OR 5.489; 95%CI 1.494-20.162) or mental health behaviours (OR 4.750; 95%CI 1.228-18.406) and those who did not perceive DITs overall well-being to be prioritised had higher odds of EE (OR 8.800; 95% CI 1.920-40.336). | When DITs perceive the hospital promotes and prioritises well-being measures and provides a supportive environment, they are less likely to experience EE. This pilot study demonstrated the value of using an international standard to measure sustainable employability in hospitals. Rural hospital administrators and policy makers can potentially use the results to guide hospital interventions aimed at alleviating EE in DITs. It appears that supportive hospital environments are key factors to consider when developing these interventions. The international standard guidelines on sustainable employability can be used by top management and human resource managers to develop sustainable employability metrics. |
| 19 | Ozen | 2021 | Comparison of emotional approaches of medical doctors against COVID-19 pandemic: Eastern and Western Mediterranean countries | Cross- sectional study | 11.5 | NA | Pandemics represent widespread disease  outbreaks with profound impacts on populations, giving rise to various life-altering events, including heightened anxiety, depression, anger, sleep disturbances, emotional distress, and stress. On March 11, the World Health Organization (WHO) declared the coronavirus disease 2019 (COVID-19) a pandemic due to the escalating cases in over 110 countries, surpassing 118,000. The pandemic has taken a toll on healthcare workers, with some falling ill and others tragically losing their lives. This study aims to assess and compare the stress levels associated with the COVID-19 pandemic among doctors in Turkey and Italy. | This study adopts a cross-sectional research design, employing the Perceived Stress Scale (PSS-10) and Secondary Traumatic Stress Scale (STSS) distributed online through social networks. The data collection instruments were disseminated to individuals from June 1 to June 15, 2020, and responses were collected using the Google Forms application. A total of 618 medical doctors were included in this study. | Elevated levels of perceived stress (PS) and secondary traumatic stress (STS) were observed in individuals identifying as female, those who were married, working in pandemic hospitals, and those in older age groups. Notably, stress levels were statistically higher among Turkish doctors compared to their Italian counterparts for both stress scales. | In comparison to Italy, Turkey has a lower number of doctors per 1000 of the population, coupled with a higher per capita rate of visits to physicians.  Beyond the challenges posed by the pandemic, these demanding working conditions, along with increased weekly working hours, may contribute to stress among Turkish doctors. Communicating such findings is crucial, and international collaborations are vital for devising future prevention strategies. It is imperative to foster stronger international connections and encourage collaborations beyond national boundaries. Furthermore, immediate interventions promoting mental well-being in healthcare professionals exposed to COVID-19 should be implemented. |
| 20 | Jacob W. Roden- Foreman | 2017 | Secondary traumatic stress in emergency medicine clinicians | Cross- Sectional study | 15 | NA | Formerly referred to as Secondary Traumatic  Stress (STS), secondary exposure to trauma is currently recognized as a valid DSM-5 Criterion A stressor for posttraumatic stress disorder (PTSD). Existing research has identified elevated rates of STS among clinicians involved in the care of traumatically injured patients. Nevertheless, there is limited research on the prevalence of STS among Emergency Medicine (EM) physicians and advanced practice providers (APPs). | The present study included Emergency Medicine (EM) providers, totaling 118 individuals, employed in one of 10 hospitals. The investigation aimed to assess risk factors, protective factors, and the prevalence of Secondary Traumatic Stress (STS) in this relatively unexplored group. The majority of participants were physicians (72.9%), identified as Caucasian (85.6%), and male (70.3%), with an average age of 39.7 (SD = 8.9). | In general, 12.7% of the sample exhibited positive screening for Secondary Traumatic Stress (STS), characterized by clinical levels of intrusion, arousal, and avoidance symptom clusters.  Additionally, 33.9% of the participants displayed at least one symptom cluster at clinical levels. | The study found that low resilience and a history of personal trauma were positively linked to positive screens for Secondary Traumatic Stress (STS) and higher STS severity scores. There was a borderline significance indicating that female gender and spending ≥10% of one's time with trauma patients might also be additional risk factors. These results imply that interventions aimed at building resilience could potentially be beneficial in mitigating the impact of STS. |
| 21 | Chan | 2015 | Addressing physician quality of life: understanding the relationship between burnout, work engagement, compassion fatigue and satisfaction | Cross sectional | 13.5 | NA | burnout and compassion fatigue are acknowledged as occupational risks within the medical field. What's fascinating is that while these challenges are prevalent, not every physician experiences them. Many healthcare professionals manage to discover fulfillment, meaning, and satisfaction in their work, despite the inherent stressors and difficulties. This study sought to determine the relationship between burnout, work engagement, compassion fatigue and satisfaction amongst doctors. | This study investigated the interconnections among burnout, work engagement, compassion fatigue, and satisfaction within the medical community, focusing on doctors. Additionally, it explored the correlations between these factors and four measurable intrinsic human traits: self-efficacy, resilient personality types, a sense of gratitude, and work calling. | The research revealed that a considerable percentage of doctors were at a high risk of experiencing burnout, while a smaller proportion were at high risk of compassion fatigue.  Conversely, only a small percentage were identified as having a low risk of burnout and compassion fatigue. A limited proportion demonstrated high levels of work engagement, and an even smaller percentage reported high levels of compassion satisfaction.  The study also found a mild but noteworthy negative correlation between burnout and engagement, along with a weak negative correlation between compassion fatigue and satisfaction. Intriguingly, the only factors significantly linked to burnout, work engagement, compassion fatigue, and satisfaction were intrinsic human traits. These findings suggest a strong association between these inherent characteristics and doctors' experiences regarding these factors. | The initial discoveries indicate that specific inherent factors contribute to higher levels of work engagement and compassion satisfaction among doctors. Interestingly, some of these intrinsic factors also seem to act as protective measures against burnout and compassion fatigue. By enhancing work engagement and compassion satisfaction, not only can individuals strengthen their resilience against burnout and compassion fatigue, but this could also potentially lead to overall improvements in health, professional quality of life, and longevity in a doctor's career. |
| 22 | Moreno- Jimenez, Jennifer E. | 2020 | Pilot study of a brief psychological intervention for reducing emotional exhaustion and secondary traumatic stress among physicians of intensive care units in Mexico | Non- randomised controlled clinical trial | 12 | NA | ICU physicians face the potential risk of  experiencing negative outcomes, including emotional exhaustion and secondary traumatic stress (STS). The job demands within these units, such as work stressors and emotional effort, are expected to positively predict these outcomes. Conversely, personal resources like harmonious passion and self-compassion may mitigate these negative effects. The study aims to create a targeted intervention for ICU physicians and offer initial evidence of its effectiveness. | A concise intervention, consisting of five weekly sessions (each lasting two hours), was implemented with four ICU physicians in a Mexican hospital.  Another four physicians were chosen as a control group. The intervention's goal was to decrease emotional exhaustion and secondary traumatic stress (STS) by fostering harmonious passion and self- compassion, while also reducing emotional effort. | The intervention group demonstrated a notable decrease in: a) work stressors, emotional effort, emotional exhaustion, and secondary traumatic stress (STS). One physician experienced a significant increase in harmonious passion, but overall, the intervention appeared to maintain high levels of both harmonious passion and self- compassion compared to the control group. The control group exhibited poorer outcomes in their results. | Despite its limitations, this study offers initial evidence for the successful reduction of emotional exhaustion and secondary traumatic stress (STS). The results underscore the importance of individually analyzing effective tools for each physician and emphasize interventions that target harmonious passion and self-compassion. This study advocates for future research to explore intervention proposals in ICUs, incorporating follow- up measures to mitigate long-term negative consequences. |
| 23 | Isobel, S. | 2018 | Neuro-reciprocity and vicarious trauma in psychiatrists | Literature Review | NA | NA | This paper seeks to provide a concise overview of the mechanisms of neuro- reciprocity that are pertinent to the vicarious traumatization experienced by psychiatrists during their clinical engagements. | NA | Neuro-reciprocity: The article discusses how empathic attunement between psychiatrists and their patients involves neurobiological processes that can lead to shared emotional experiences. This neuro-reciprocity, while essential for effective therapy, may also render psychiatrists susceptible to vicarious trauma.  Vicarious Trauma in Psychiatrists: Due to high rates of trauma exposure among mental health service users, psychiatrists frequently encounter traumatic narratives. The cumulative effect of these interactions can lead to vicarious traumatization, characterized by changes in the clinician's cognitive schemas and emotional well-being  Trauma-Informed Care: The authors highlight the importance of implementing trauma-informed approaches at an organizational level to prevent vicarious trauma. Such approaches include providing adequate support systems, promoting self-care among clinicians, and fostering an environment that acknowledges the impact of trauma on both patients and healthcare providers. | Given the elevated prevalence of trauma among  individuals utilizing mental health services, coupled with an understanding of how trauma impacts the brain and the mechanisms of neuro-reciprocity involved in empathic attunement, psychiatrists face a heightened risk of experiencing vicarious trauma. Consequently, instituting trauma-informed approaches at an organizational level becomes critically important in preventing vicarious trauma among psychiatrists. |
| 24 | Doolittle | 2020 | Association of burnout with emotional coping strategies, friendship, and institutional support among internal medicine physicians | Cross- sectional study | 12.5 | NA | Physician burnout has wide-ranging effects on physician mental well-being, staff stress, safety events, and patient outcomes. However, there is limited research on the correlation between burnout and factors like compassion satisfaction, secondary stress, emotional coping strategies, and various psychosocial variables such as institutional support, friendship, and spirituality. This study aims to investigate and understand these associations. | A survey utilizing validated instruments was emailed to a convenience sample of internal medicine physicians to investigate the associations mentioned earlier. The response rate was 33%, with 337/1021 physicians participating. Within the responding group, 52% reported experiencing burnout, totaling 175 individuals. | Several factors, including grit, acceptance, active  coping, positive reframing, and strategy planning, were linked to lower burnout levels and increased compassion satisfaction. On the contrary, certain emotional coping strategies such as denial, disengagement, self-blame, substance abuse, and venting were associated with higher burnout and reduced compassion satisfaction. Institutional support showed a significant association with lower burnout, secondary stress, and greater compassion satisfaction. Additionally, friendship was linked to lower burnout and increased compassion satisfaction. | The findings of this study propose that alleviating burnout necessitates a combination of intrinsic strategies focusing on physician coping skills and extrinsic strategies that tackle institutional support. |
| 25 | Bhutani | 2012 | Compassion fatigue and burnout amongst clinicians: a medical exploratory study | Cross- sectional study | 11.5 | NA | Compassion fatigue encompasses two primary components: burnout and secondary traumatic stress. This study aims to discern the presence of both 'burnout' and 'compassion fatigue' among clinicians engaged in the care of patients. | The study comprised 60 clinicians, and a semi- structured questionnaire was employed to collect information concerning the participants' personal, professional, anthropometric, and metabolic profiles. The Professional Quality of Life Scale (ProQoL Version V) was utilized to evaluate burnout, compassion satisfaction, and secondary traumatic stress. Statistical analysis was conducted using SPSS version 19.0. | The average age of the clinicians was 46.68 ±  11.06 years, ranging from 26 to 67 years. Notably, those engaged in diabetology practice exhibited significantly higher burnout scores. Additionally, greater years of practice and working in private practice were associated with higher compassion satisfaction scores. Clinicians reporting poor working conditions, in contrast to those reporting good conditions, experienced more burnout and less compassion satisfaction. | The findings from the present study emphasize the importance of identifying strategies to reduce burnout and compassion fatigue among clinicians. |
| 26 | Abdelhadi Ibrahim, B. | 2022 | Professional quality of life among physicians of tertiary care hospitals: An Egyptian cross- sectional study | Cross- sectional study | 11.5 | NA | This study aim to evaluate the elements of professional quality of life and identify the factors that influence it, recognizing its significant impact on the well-being and performance of professionals in the caregiving field. | The cross-sectional study involved 167 physicians selected through stratified random sampling from tertiary care hospitals in Ismailia, Egypt. A structured interview questionnaire was utilized, which included the Maslach Burnout Inventory to assess burnout syndrome and the Professional Quality of Life version 5 (Pro QOL-5) subscale for evaluating compassion fatigue and satisfaction. | Among the surveyed participants, 78.9% exhibited  high burnout levels, 76% demonstrated moderate potential for compassion satisfaction, and 82% showed moderate potential for compassion fatigue. The correlation between different scales of professional quality of life scores yielded significant results. Multiple linear regression analysis identified marital status, frequency of dealing with critical patients, and compassion fatigue score as significant predictors of burnout score. Marital status and burnout score were positive predictors of compassion fatigue, while compassion satisfaction score served as a negative predictor. The predictors of compassion satisfaction included marital status and compassion fatigue score. | Physicians in tertiary care hospitals exhibited high prevalence rates of burnout, compassion fatigue, and satisfaction, indicating a poor professional quality of life. |
| 27 | Austin, C. L. | 2017 | Moral distress in physicians and nurses: Impact on professional quality of life and turnover | Cross- Sectional Study | 12.5 | NA | Thi study aimed to examine the correlation between moral distress (MD) and their willingness to voluntarily leave their jobs, in relation to their professional quality of life, amongst doctors and nurses at a teertiary care hospital. | This study involved the anonymous participation of healthcare professionals from various hospital departments and required them to complete two validated questionnaires, namely the Moral Distress Scale–Revised and the Professional Quality of Life Scale. Professional quality of life was measured using Compassion Fatigue (assessed through secondary traumatic stress (STS) and burnout (BRN)) and Compassion Satisfaction as subscales. The study investigated the associations between these aspects and clinicians' years of experience in healthcare, amount of patients who required critical care, and their professional discipline. | The results, involving 329 participants, revealed  notable correlations between secondary traumatic stress (STS), burnout (BRN), and moral distress (MD). The level of MD was calculated by scores reflecting the inclination for participants to either stay or leave their jobs. This study highlights the cases with the highest scores of MD and the circumstances in which doctors and nurses appear to be most susceptible to experiencing STS, BRN, and MD. Participants, including doctors and nurses, pointed out that factors associated with the highest levels of MD included being obligated to deliver seemingly ineffective care and having to work in a setting where the patient load for critical care patients exceeded 50%. | This study found a significant influence of moral distress (MD) on turnover intention and professional quality of life. Consequently, there is a need to put in place initiatives focusing on the emotional wellbeing of healthcare professionals, such as discussions about ethical dilemmas, empowerment and implementing relevant policies. Additionally, it is recommended to regularly monitor the wellbeing of physicians and nurses and have routine screenings which assess them for maladaptive characteristics. |
| 28 | Kamajian, Steven | 2022 | When Helping Hurts: Compassion Fatigue in Family Physicians | Grey Literature | NA | NA | This article examines the phenomenon of compassion fatigue (CF) among family physicians, highlighting the emotional and physical exhaustion that can result from prolonged exposure to patient suffering and high-stress environments. It emphasizes the importance of recognizing CF to maintain physician well-being and ensure quality patient care. | NA | Definition of Compassion Fatigue: CF is described as a state where physicians, due to continuous exposure to patient distress, experience a decline in their ability to empathize, leading to emotional exhaustion and reduced compassion over time.  Symptoms and Indicators: The article outlines common symptoms of CF, including deep physical and emotional exhaustion, a pronounced change in the caregiver's ability to empathize, and a decline in the quality of care provided.  Differentiation from Burnout: While both CF and burnout involve emotional exhaustion, CF is specifically associated with the caregiver's exposure to patients' traumatic experiences, whereas burnout relates more broadly to work-related stress and overload.  Impact on Physicians: The article discusses how CF can lead to decreased job satisfaction, increased absenteeism, and a higher likelihood of medical errors, ultimately affecting both physician health and patient outcomes.  Management and Prevention Strategies: Recommendations include seeking support through talking with colleagues or professionals, understanding that experiencing pain is normal, maintaining physical health through proper diet and exercise, ensuring adequate sleep, taking time off when needed, developing interests outside of medicine, and identifying personal values and priorities. | Physicians frequently have experiences working  with patients who are suffering from trauma and this can negatively affect their psychological wellbeing. In the context of the COVID-19 pandemic, physicians have experienced even more fatigue, stress, and sadness. There is a suggestion that physicians may be able to feel more satisfied and be better able to show more compassion to patients if they shift their expectations to a more spiritually derived intent to care for them, instead if merely conquering disease and overcoming death. To persist in doing good despite these challenges, physicians must learn to proactively recognise CF, implement protective practices against CF such as self care and work life balance and intervene early in CF to reduce the physical and mental effects on physicians. There is also a need to prevent CF as having CF resilience can help family physicians remain compassionate in caring for patients, over the long term course. This has benefits on the quality of life for both patients and physicians. |
| 29 | Karlafti, Eleni | 2022 | Exhaustion and burnout in the healthcare system in Greece: A cross-sectional study among internists during the COVID-19 lockdown | Cross- Sectional Study | 11.5 | NA | There has been a lot of impact of the  COVID-19 pandemic on people's lives around the world. As a result of the circumstances created by the COVID-19 pandemic, there has been serious emotional and psychological impacts observed in frontline medical and nursing staff. This study's objective is to explore various psychological aspects, including exhaustion, disengagement, secondary traumatic stress, compassion satisfaction, burnout, as well as depression, anxiety, and stress among internists in Greece. The investigation is specifically focused on the context of the second lockdown period during the COVID-19 pandemic. | This study recruited internists through the Internal Medicine Society of Greece, with a total of 117 participants, and a response rate of 15.3%.  Participants filled in a Google form which included questions to find out more about their emotional and psychological wellbeing. Measurement tools included Depression, Anxiety and Stress Scale-21, the Oldenburg Burnout Inventory (OLBI) and the Professional Quality of Life Scale version 5  (ProQOL-5). | Secondary Traumatic Stress (STS) was predominantly reported at a moderate level in the studied sample, with 64.8% of participants experiencing it at this level | The researchers found that most of the internists in Greece were "exhausted" and were experiencing a toll on their psychological and emotional health as there were high levels of negative psychological and emotional symptoms. Thus, it is important to be aware of the negative effects of the COVID-19 pandemic on the psychological and emotional health of internists. There is a need to focus on interventions for this and prevention of stressful situations for healthcare professionals. |
| 30 | Vacca | 2021 | The emotional impact of the operator in the care of patients with mental disorders during the pandemic: Measure of interventions on compassion fatigue and burn-out | Cross- Sectional Study | 13 | NA | The COVID-19 pandemic has exerted a profound impact on various facets of society, including mental and physical health. This study explores compassion fatigue, empathy decline and lack of hope linked to burnout in healthcare providers, in the discipline of psychiatric rehabilitation. | This study consists of 87 healthcare professionals from 3 different psychiatric rehabilitation communities and explored compassion fatigue, vicarious trauma, burnout and hope (hopeless), empathy and depressive symptoms in them, with the use of measurement scales from May to June 2021. This study made use of the following measurement scales; ProQOL, BHS, SAVE-9, BDI-II and BEES. | This study found significant levels of compassion fatigue in participants, varying levels of burnout with burnout being absent in some groups. This study also found an association between high levels of hope, compassion satisfaction with moderate levles of empathy. This suggests that high levels of empathy in healthcare workers may reduce their risk of suffering from stress and mental health problems secondary to work stressors. | The findings from this study highlights the  significance of factors such as hope and empathy in healthcare workers caring and providing rehabilitation for patients with psychiatric disorders. This highlights the necessity for implementation of strategies, which target managing emotions and mental health of care providers, to improve quality of care to patients and reduce occurrence of burnout. This is especially relevant in the settings of the pandemic which renders very stressful situations. |
| 31 | Granek, L. | 2017 | Oncologists' communication about end of life: the relationship among secondary traumatic stress, compassion satisfaction, and approach and avoidance communication | Cross sectional | 12 | NA | The goal of this study was to investigate the connections between secondary traumatic stress (STS) and compassion satisfaction (CS) and the manner in which oncologists engage in conversations about end-of-life (EOL) topics with cancer patients. Specifically, the study aimed to explore how these mental health factors relate to both approachable and avoidant communication regarding end-of-life matters. | In this study, a convenient sample of 79 oncologists (comprising 27 men and 52 women) took part. These oncologists were asked to complete a survey that encompassed questions related to their sociodemographic background, clinical information, the Professional Quality Of Life Scale, and the Communication about End of Life Survey. To explore the impact of secondary traumatic stress and compassion satisfaction on both approachable and avoidant communication, while considering the influence of gender and age, the researchers conducted two hierarchical linear regression analyses. | The oncologists in the study indicated elevated levels of both secondary traumatic stress and compassion satisfaction. Their scores on the approach and avoidant communication scales fell in the middle of the rating scale. Lower levels of reported secondary traumatic stress and higher compassion satisfaction were linked to a to employing higher approach communication strategies. However, only higher levels of secondary traumatic stress were associated with employing avoidant communication strategies. | The results underscore the relationship between emotional factors and the approach to communication. These findings hold clinical significance, particularly in shaping the design of communication skills training. It's crucial for further research and training initiatives to consider the influence of secondary traumatic stress and compassion satisfaction as incorporating these factors into training programs can enhance the quality of end-of-life care delivered to terminal patients and their families. |
| 32 | Braun, M. | 2022 | Oncologists’ Locus of Control, Compassion Fatigue, Compassion Satisfaction, and the Mediating Role of Helplessness | Cross sectional | 12.5 | NA | The study delves into how oncologists' sense of control, specifically their locus of control (LOC), might impact their experience of compassion fatigue and satisfaction. Locus of control, based on Rotter's definition, refers to how individuals attribute causes to events in their lives. Those with an internal LOC believe their efforts influence outcomes, while those with an external LOC see events as independent of their actions, often attributing them to fate or external forces. The research also explores emotional variables like guilt and helplessness as potential mediators, aiming to explain the connections between LOC and oncologists' experience of compassion fatigue. This investigation provides these variables as potential explanatory factors for understanding how LOC influences compassion fatigue among oncologists. | A group of 73 oncologists participated by completing several questionnaires - the Professional Quality of Life scale, Levenson’s Internal, Powerful Others, and Chance scale, the Guilt Inventory, specifically focusing on the State Guilt subscale, and the Learned Helplessness scale. | The study revealed that oncologists reported elevated levels of secondary traumatic stress and burnout, along with moderate levels of compassion satisfaction. It was observed that there was a direct positive relationship between oncologists’ external locus of control (LOC) and compassion fatigue. Conversely, an inverse association was identified between oncologists’ internal LOC and compassion fatigue. Moreover, helplessness, but not guilt, played a mediating role in these connections. Additionally, internal LOC showed a positive correlation with compassion satisfaction. | This study underscores that oncologists are susceptible to compassion fatigue and underscores the significance of their locus of control in influencing this experience. Furthermore, both the cognitive and emotional dimensions of control were identified as crucial factors linked to the development of compassion fatigue among oncologists. |
| 33 | Kearney, M. K. et al. | 2009 | Self-care of physicians caring for patients at the end of life: "Being connected... A key to my survival" | Perspective | NA | NA | The article aims to explore the challenges and psychological strains faced by physicians providing end-of-life care. It examines how self-care practices, empathy, and professional boundaries influence physician well-being and the quality of care delivered to terminally ill patients. | Design: A narrative, qualitative exploration of physician experiences with self-care and compassion fatigue.  Participants: Physicians engaged in end-of-life care, focusing on their personal accounts and reflections.  Data Sources: Real-life case studies and interviews from clinicians providing end-of-life care, integrated with literature on burnout, compassion fatigue, and self-care practices | 1. Compassion Fatigue and Burnout:  Physicians often experience burnout driven by work-related stress and compassion fatigue stemming from empathic involvement with patients and their families.  Signs include emotional exhaustion, depersonalization, and a diminished sense of personal accomplishment.  2. Self-Care and Coping Strategies:  Effective self-care practices such as mindfulness meditation, reflective writing, peer support, and maintaining professional boundaries are critical for mitigating burnout.  Empathy, when well-boundaried and practiced as "exquisite empathy," can invigorate rather than deplete clinicians.  3. Factors Mitigating Stress:  A supportive work environment, opportunities for personal and professional growth, and fostering self-awareness are vital in reducing stress and enhancing job satisfaction.  4. Impact on Patient Care:  Physicians who balance self-care with empathy report greater professional fulfillment and provide better end-of-life care, facilitating meaningful patient-clinician relationships. | The study emphasizes the importance of clinician self-awareness and self-care as integral components of sustainable and compassionate end-of-life care. It highlights the need for institutional support and personal practices that bolster resilience and promote professional satisfaction among physicians. |
| 34 | Gleichgerrc ht, E. | 2014 | The relationship between different facets of empathy, pain perception and compassion fatigue among physicians | Cross- Sectional Study | 12 | NA | Physicians are consistently confronted with the suffering and distress of patients. By having a better conceptualisation of the connection between pain perception, empathetic tendencies, and professional quality of life, effective strategies to safeguard health professionals from burnout and compassion fatigue can be formed. This study aims to examine the correlation between individual attitudes and behavioral indicators of pain sensitivity, empathy, and professional quality of life. | 1,199 board-certified physicians were asked to complete a behavioural task, a set of self-report measures on a web based platform. The behavioural task involved participants watching video clips depicting patients experiencing varying levels of pain and providing ratings for both pain intensity and induced personal distress. Surveys were also conducted to collect information on demographic and professional backgrounds, dispositional empathy (including empathic concern, personal distress, and perspective taking), as well as positive (compassion satisfaction) and negative (burnout and secondary traumatic stress) aspects of their professional lives. | Experienced physicians perceived lower pain  intensity. The levels of perceived pain was similar amongst these physicians regardless of the emotional stress demands associated with their different specialty fields. However, when watching videos of patients in pain, physicians in high-stress medical fields experienced greater personal distress, despite comparable empathy dispositions with other disciplines. There was a tendency for male patients' pain to be perceived as less than that of female patients, and this effect was more pronounced among female physicians.  Dispositional empathy's impact on perception of pain and induced personal distress varied for each sub-category. Perspective-taking and empathic concern (EC) were predictors for behavioral outcomes. Physicians who simultaneously experience both compassion satisfaction and fatigue tend to perceive more pain and suffer greater personal distress than those who only experience negative effects in the aspect of their  professional quality of life. | Experience in the healthcare industry appears to make physicians desensitised to the pain experienced by others, yet this does not aid them in reducing their trauma from exposure to other's pain. The perception of pain is also connected to certain facets of empathy and is influenced by contextual factors, such as the gender of their patients. It seems that high levels of empathy are not necessarily required for physicians to be able to gain from the positive aspects of professional quality of life in medicine. |
| 35 | Ghazanfar | 2018 | Compassion Satisfaction, Burnout, and Compassion Fatigue in Cardiac Physicians Working in Tertiary Care Cardiac Hospitals in Pakistan | Cross sectional | 13 | 10 | The study aims to identify how prevalent compassion satisfaction, compassion fatigue, and burnout are among cardiac physicians employed in tertiary care cardiac hospitals in Pakistan, as well as the potential risk factors associated with these conditions. | During a cross-sectional study conducted between  June 2017 and January 2018 in four tertiary care cardiac hospitals in Rawalpindi and Lahore, Pakistan, a three-stage approach was employed. Initially, the Professional Quality of Life Scale (ProQOL-5) questionnaire was administered to assess compassion satisfaction, compassion fatigue, and burnout among cardiac physicians. In the subsequent stage, physicians were categorized based on their compassion fatigue levels. Finally, through convenience sampling, 50 participants engaged in 15-minute interviews discussing compassion fatigue and associated risk factors. Data analysis was performed using IBM SPSS version 21.0 | Among the 200 participants, 55.0% were male, and 45.0% were female. The mean score for compassion satisfaction was 39.13 ± 5.54, while for burnout, it was 24.7 ± 4.28, and for secondary traumatic stress (compassion fatigue), it was 25.97  ± 6.39. Those under 40 years old had higher scores in both burnout and secondary traumatic stress categories. | Raising awareness about compassion fatigue and burnout among cardiac care physicians in Pakistan is crucial, given the lack of attention to these issues despite their significant impact. Increasing awareness can help address these challenges and support the well-being of healthcare providers in cardiac care settings. |
| 36 | Kleiner, S. | 2017 | Oncologist burnout and compassion fatigue: investigating time pressure at work as a predictor and the mediating role of work-family conflict | Cross sectional | 11 | NA | Oncologists face a heightened risk of  experiencing poor mental health. Previous studies have primarily concentrated on burnout, pinpointing a heavy workload as a significant predictor. Compassion fatigue, a less explored phenomenon among physicians overall, might be particularly prevalent in medical specialties like oncology. Our contribution to this research lies in identifying factors predicting both burnout and compassion fatigue among oncologists. We aim to differentiate between quantitative workload factors, such as work hours, and subjective work pressure. Additionally, we're examining whether work-family conflict acts as a mediator in the connections between work pressure and the experiences of burnout or compassion fatigue. | In a cross-sectional study, a group of oncologists from various regions in Canada (totaling 312 participants) filled out questionnaires aimed at evaluating burnout, compassion fatigue, workload, time pressure at work, work-family conflict, and additional personal, family, and occupational traits. The analysis of the collected data involved using Ordinary Least Squares regression as the primary statistical method. | The study highlights that perceived time pressure at work significantly predicts both burnout and compassion fatigue among oncologists.  Additionally, the findings indicate that work-family conflict fully mediates these associations between time pressure and the experiences of burnout and compassion fatigue. All in all, the models employed in the study explain a greater degree of variation in burnout compared to compassion fatigue. | Our research emphasizes the importance of incorporating subjective time pressure, alongside quantitative workload, in interventions aimed at enhancing the mental well-being of oncologists. Furthermore, the results underscore the necessity of delving deeper into comprehending other factors that might predict compassion fatigue among these professionals. |
| 37 | Kiliç Durankuş, N. | 2021 | Increased levels of anxiety, depression, and secondary trauma in radiation oncologists during covid-19 pandemic: A preliminary report from turkey | Cross- Sectional Study | 12 | NA | The study aimed to assess the levels of anxiety, depression, and secondary trauma among Turkish Radiation Oncologists in relation to the COVID-19 pandemic. | This study developed an online questionnaire which incorporated inquiries about demographics and occupational status, along with the administration of standardized assessment tools, including the Secondary Traumatic Stress Scale (STSSS), The Beck Depression Inventory (BDI), The Beck Anxiety Inventory (BAI), and the State-Trait Anxiety Inventory (STAI). It was targeted at Turkish Radiation Oncologists and responses were anonymous. | Resarchers found that there were greater mental  health effects of COVID-19 and social isolation on those who had a Beck Depression Inventory (BDI) score or more than 19, as compared to those who had a BDI score of 19 and below. This group also had higher STSS, STAIS, STAIT, BAI and BDI scores, indicating that they experience higher levels of secondary traumatic stress, state and trait anxiety, and depression. This study also found that there is a significant relationship between BAI, BDI, STAIT, STAIS, STSS, mental health effects of COVID-19 and social isolation. | This study is one of the first that explores the effect of the COVID-19 pandemic on depression, anxiety levels, and secondary trauma in Radiation Oncologists. Notably, this group does not partake in direct patient contact with COVID-19 positive patients. |
| 38 | Shekinah Manning- Jones, | 2016 | Secondary traumatic stress, vicarious posttraumatic  growth, and coping among health professionals; A  comparison study. | Cross- Sectional study | 14 | NA | The present study delves into the connection between coping strategies and the psychological outcomes associated with vicarious traumatic exposure. Additionally, it seeks to examine inter-specialty differences among five distinct groups of health professionals. | The authors recruited 365 health professionals,  including social workers, nurses, counselors, psychologists, and medical doctors, to participate in the study. The participants completed three self- report measures: the Posttraumatic Growth Inventory (PTGI), the Secondary Traumatic Stress Scale (STSS), and the Traumatic Stress Schedule (TSS). The PTGI was used to measure vicarious posttraumatic growth, the STSS was used to measure secondary traumatic stress, and the TSS was used to measure direct traumatic exposure. The authors also collected demographic information, including age, gender, ethnicity, and years of experience. The data were analyzed using Pearson's r correlation matrices, t-test analysis, and quadratic hierarchical multiple regression. | The findings indicated that engaging in self-care and receiving social support from friends and family were negatively associated with the occurrence of secondary traumatic stress (STS). On the other hand, utilizing humor, practicing self- care, and receiving support from peers were positively linked to vicarious posttraumatic growth (VPTG). | Social workers encountered the most significant levels of secondary traumatic stress (STS) and post-traumatic growth (VPTG), with psychologists registering the lowest levels for both variables. In terms of coping, psychologists and counselors  generally reported the most extensive use of coping strategies, whereas doctors and nurses reported the least utilization. However, a contrasting trend emerged for peer social support, as nurses reported notably higher levels of support from peers compared to psychologists. The implications of these findings are explored in the discussion. |
| 39 | Dasan | 2015 | Prevalence, causes and consequences of compassion satisfaction and compassion fatigue in emergency care: a mixed-methods study of UK NHS Consultants | Cross- sectional study | 12.5 | NA | The study aims to determine the prevalence of compassion satisfaction and compassion fatigue among emergency medicine consultants in the UK and to investigate potential causes and consequences associated with these phenomena. | The study employs a sequential mixed-methods design, starting with a cross-sectional e-survey distributed to all UK NHS emergency medicine consultants (n=1317). The survey includes the assessment of Professional Quality of Life (ProQOL) to measure compassion satisfaction and fatigue.  Subsequently, interviews will be conducted with a subset of consultants, specifically those scoring above (n=6) and below (n=6) predefined ProQOL thresholds | In the survey, 52% of the consultants (681) responded, with a significant majority (98%) reporting at least an 'average' level of compassion satisfaction. Higher satisfaction scores were linked to specific workplace characteristics, with designated trauma centers performing better. The number of years worked as a consultant showed a gradual decline in satisfaction over time, except for those with 20 years or more of experience, where satisfaction improved. Consultants with lower satisfaction scores were more likely to exhibit behaviors such as irritability with patients or colleagues, reduced standards of care (reported by a third at least monthly), and expressed a higher likelihood of intending to retire early (59% had such plans). The distinguishing features between 'satisfied' and 'fatigued' consultants in interviews included having effective strevalence and factors assorategies to cope with high work intensities, positive perceptions of their team, the degree of variety in their roles, and the ability to maintain empathy for their patients. | The results advocate for an immediate reassessment of workforce and resources in emergency medicine. The findings suggest a comprehensive approach is needed to identify, prevent, and treat occupational stress in the workforce. This approach should take into account individual, job, and organizational factors, with a particular focus on aspects influencing perceived control and support at work. |
| 40 | Kartsonaki, M. G. et al. | 2023 | Prevalence and factors associated with compassion fatigue, compassion satisfaction, burnout in health professionals | Cross-sectional study | 11.5 | NA | Constant exposure of health professionals to the pain and suffering of patients can adversely affect their emotional wellbeing. The study aimed to investigate the prevalence and the factors affecting the levels of secondary traumatic stress/compassion fatigue (STS/CF), burnout (BO) and compassion satisfaction (CS) of health professionals working in adult and paediatric Intensive Care Units (ICU) as well as in departments treating patients with serious illness in five hospitals in Crete. | A descriptive cross-sectional survey with the use of the Professional Quality of Life Scale (ProQOL-CSF-R-IV) questionnaire. Medical, nursing and support staff working in the adult and paediatric ICU, emergency, oncology, haematology and neurosurgical departments, haemodialysis unit and operating theatre were enrolled in the study. | 598 health professionals completed the questionnaire (response rate 73.2%). Significantly increased levels of STS/CF were observed in non - ICU as compared to ICU staff (p = .009) females compared to males (p < .001), those who have previously experienced a traumatic event (p < .004), nurses and support staff compared to doctors (p = .007 and p = .028 respectively), and people not working in a department by choice (p < .001). CS was higher for older professionals, personnel subjected to stress reduction techniques (p < .019) and professionals working with children or mixed adults and children population (p = .009). Rolling schedule and bad working conditions negatively affected CS (p = .02, p = .001). Increased BO levels were associated with younger age (p = .029) and showed a positive correlation with STS/CF (r = .356, p < .001). | STS/CF is common in health professionals regardless of their profession, working department or hospital level. Non-ICU staff displayed higher STS/CF levels. Working in a department by choice ameliorates CS, BO and STS/CF. |
| 41 | Nimmo,  Arohaina | 2013 | A systematic review of the measurement of compassion fatigue, vicarious trauma, and secondary traumatic stress in physicians | Systematic review | NA | NA | The emotional impact on healthcare professionals, stemming from their interactions with traumatized patients, is often encapsulated by terms like compassion fatigue, vicarious traumatization, and secondary traumatic stress. While these terms are sometimes used interchangeably, recent perspectives suggest nuanced differences between them. The repercussions of enduring emotional distress due to patient interactions are equally significant for physicians compared to other healthcare providers. However, these particular constructs have not been thoroughly explored within the physician workforce. This article presents findings from a systematic review of literature that delves into one or more of these constructs, including efforts made to measure and understand them. | The researchers conducted a systematic review of scholarly articles by extensively searching electronic literature databases. They followed established methodologies and quality criteria to guide their search and evaluation of literature. Initial searches helped identify suitable databases and guided the creation of specific search conditions. The criteria for inclusion and exclusion were applied to refine the literature search, resulting in a limited number of articles.  Numerous methodologies and guidelines exist for reporting systematic reviews and meta-analyses in healthcare research, particularly for quantitative studies like observational studies, randomized controlled trials, and public health interventions.  Among these, the PRISMA protocol was selected for this study because of its applicability to both meta- analytical and systematic literature reviews. | The findings from the review indicate that physicians might indeed face experiences of compassion fatigue and vicarious trauma.  However, the absence of articles specifically measuring secondary traumatic stress in physicians was notable, possibly suggesting a limited acknowledgment or use of this term within health professional literature. Key discoveries included an inverse relationship between physician age and compassion fatigue/vicarious trauma.  Additionally, a significant association was identified between compassion fatigue and spirituality. The type of care provided by physicians also played a role in the risk of developing compassion fatigue, with those involved in sexual abuse therapy or HIV-AIDS treatment being at higher risk. | The study's outcomes underscore the minimal attention given to researching the presence of compassion fatigue, vicarious trauma, and secondary traumatic stress among physicians, despite these being reported as negative experiences within this professional group. This scarcity in literature signals an opportunity for further investigation. Another critical research avenue involves assessing the impact of preventive or mitigating interventions aimed at helping physicians manage the effects of compassion fatigue, vicarious traumatization, or secondary traumatic stress. |
| 42 | Latsou, Dimitra | 2022 | Professional Quality of Life and Occupational Stress in Healthcare Professionals During the  COVID-19 Pandemic in Greece | Cross- sectional study | 12 | NA | Healthcare professionals (HPs) dealing with the challenges posed by the COVID-19 pandemic face the potential of working in stressful environments that can affect their professional well-being. The aim of this study is to investigate the professional quality of life and occupational stress experienced by HPs in Greece amid the COVID-19 pandemic. | A cross-sectional study was undertaken in a prominent COVID-19 reference hospital in Attica, Greece, from October to December 2020. Utilizing convenience sampling, data was collected through an anonymous, self-administered questionnaire that included demographic and occupational information, the Professional Quality of Life Scale (ProQoL), and the Job Stress Measure. A total of 250 questionnaires were distributed to physicians and nurses, with 186 questionnaires fully completed, yielding a response rate of 78.8%. The analysis involved descriptive and inductive statistics using SPSS v25. | Participants, with a mean age of 41.5 ± 10.4 years,  comprised 75.3% women and 62.4% nursing staff. ProQoL analysis indicated that a majority reported moderate levels of compassion satisfaction (74.2%) and burnout (78.5%), while 48.8% experienced a moderate level of secondary post- traumatic stress. The mean occupational stress level was 2.76, signifying a moderate stress level. Demographic and occupational characteristics of health professionals were found to influence both work stress and ProQoL. Occupational stress demonstrated a positive correlation with both burnout and secondary post-traumatic stress, implying that an increase in stress at work for health professionals corresponds to a simultaneous increase in ProQoL. | Healthcare professionals (HPs) in Greece experienced moderate professional quality of life and occupational stress during the COVID-19 pandemic. To enhance the overall well-being of HPs, there is a need to focus on strengthening the healthcare system's capacity and providing support for stress management and psychological resilience among healthcare professionals. |
| 43 | Salur | 2021 | Compassion fatigue in chest disease clinicians: The effect of psychological capital and the relationship between colleagues | Cross sectional | 12 | 13 | The study aimed to evaluate the extent of compassion fatigue and explore potential predictors—such as demographics, job-related characteristics, satisfaction levels, and psychological capital—in physicians and nurses working within a chest diseases and thoracic surgery hospital. The objective is to gain a better understanding of compassion fatigue to aid in defining, preventing, and implementing interventions for this phenomenon. | In this cross-sectional study, 205 clinicians— comprising 83 physicians and 122 nurses—directly involved in caring for in-patients participated.  Information was gathered through a semi-structured interview, the Professional Quality of Life Scale, and the Psychological Capital Scale. | The participants had an average age of 31.96 ±  8.60 years, with a majority being women. The study noted a statistically significant difference in the average compassion fatigue scores between physicians and nurses,with physicians scoring higher.  However, upon conducting regression analysis, the profession itself was not found to have a significant impact. Dissatisfaction with colleagues was identified as a factor that increased compassion fatigue by 2.5 times .Additionally, low resilience, specifically within one of the Psychological Capital Scale subscales, was linked to a 2.14-fold increase in compassion fatigue. Other professional and demographic factors did not show significant associations with compassion fatigue. | The study findings underscore the significance of strong relationships and resilience among colleagues as crucial in preventing compassion fatigue. Additionally, the research highlights that physicians are particularly at risk of experiencing compassion fatigue. |
| 44 | Gleichgerrcht, E. and Decety, J. | 2013 | Empathy in clinical practice: How individual dispositions, gender, and experience moderate empathic concern, burnout, and emotional distress in physicians | Cross sectional study | 15 | NA | In a comprehensive study involving 7,584 board-certified practicing physicians, the aim was to gain a deeper understanding of clinical empathy and identify factors that could hinder its experience and outcomes in caregiving settings. | Validated online instruments were utilized to assess various dimensions of empathy, distress, burnout, altruistic behavior, emotional awareness, and well- being. | The results revealed a strong association between  compassion satisfaction and empathic concern, perspective taking, and altruism. Conversely, compassion fatigue (comprising burnout and secondary traumatic stress) was more closely linked to personal distress and alexithymia.  Gender played a significant role, particularly affecting empathic concern, with women exhibiting higher values, leading to a range of negative and devalued emotions. Interestingly, years of experience did not independently influence dispositional measures, once the effects of age and gender were controlled. Those experiencing compassion fatigue without much compassion satisfaction demonstrated the highest scores in personal distress and alexithymia, as well as the most pronounced indicators of compassion fatigue. | Physicians struggling to regulate negative arousal and articulate and identify emotions appeared more susceptible to emotional exhaustion, detachment, and a diminished sense of accomplishment. On the contrary, the ability to engage in self-other awareness, regulate emotions, and possess a predisposition to assist others appeared to contribute to the sense of compassion derived from aiding patients in clinical practice. |
| 45 | Franco, P. | 2020 | Professional quality of life and burnout amongst radiation oncologists: The impact of alexithymia and empathy | Cross- sectional study | 12 | NA | Various elements can impact the professional quality of life among oncology professionals, with personality traits such as alexithymia and empathy being insufficiently explored.  Alexithymia involves deficits in emotion processing and awareness, while empathy refers to the ability to understand another person's state of mind or emotion. The PROject on BurnOut in RadiatioN Oncology (PRO BONO) is dedicated to assessing professional quality of life, including burnout, within the field of radiation oncology, and delves into the roles of alexithymia and empathy as potential contributing factors. | An online survey targeting ESTRO members  involved the completion of three validated questionnaires to assess alexithymia, empathy, and professional quality of life. The questionnaires utilized were the Toronto Alexithymia Scale, the Interpersonal Reactivity Index, and the Professional Quality of Life Scale. In this analysis, specifically focusing on radiation/clinical oncologists, the study examines Compassion Satisfaction (CS), Secondary Traumatic Stress (STS), and Burnout. The analysis also explores correlations with alexithymia and various aspects of empathy (empathic concern, perspective taking, and personal distress) using generalized linear modeling. Univariate linear regression identified significant covariates, which were then included in the multivariate linear regression model. | The study involved 825 radiation oncologists who completed all questionnaires. Elevated levels of alexithymia were linked to a decrease in Compassion Satisfaction (CS) and an increase in both Secondary Traumatic Stress (STS) and burnout. Higher empathic concern was significantly associated with increased CS and STS, with no impact on burnout. Personal distress was linked to a decrease in CS and an increase in both STS and burnout. | The study revealed that having the personality trait of alexithymia increases the likelihood of experiencing burnout along with reduced professional satisfaction. Conversely, empathic concern was associated with heightened stress but did not result in burnout, contributing to a greater sense of professional fulfillment. These findings can serve as reference points for implementing preventive strategies, such as enforcing work-hour limitations, fostering peer support, conducting debriefing sessions, and introducing leadership initiatives to support professionals at risk. |
| 46 | Yaakubov L | 2020 | Secondary traumatic stress, vicarious posttraumatic growth and their association in emergency room physicians and nurses | Cross- Sectional study | 13 | NA | Indirect exposure to trauma, remains underexplored in emergency room personnel. While limited studies have investigated secondary traumatic stress in emergency room nurses, and only one study has focused on emergency room physicians, the scope of vicarious post-traumatic growth—defined as the positive psychological changes associated with indirect exposure to trauma—has not been extensively studied in this specific group of healthcare professionals. | Our first goal was to examine secondary  traumatization in both emergency room nurses and physicians. Our second goal was to examine vicarious post-traumatic growth in emergency room personnel. Finally, we also address the association (linear and curvilinear) between secondary traumatization and vicarious traumatic growth.  Methods: A questionnaire comprising demographic variables, secondary traumatic stress and vicarious post-traumatic growth was administered  electronically to a sample of emergency room personnel from the Wolfson Hospital, Holon, Israel. | Nurses and physicians did not exhibit variations in  their overall levels of secondary trauma or vicarious post-traumatic growth. Among physicians, there was both a linear and a curvilinear relationship between secondary trauma and vicarious post-traumatic growth. In contrast, nurses did not display an overall association between these variables. However, upon conducting additional subgroup analyses, it was found that emergency room nurses with low workload and limited work experience did demonstrate a linear association. | The findings suggest that, in emergency room  physicians, there exists an association between vicarious post-traumatic growth and secondary traumatic stress, which is not observed in nurses. Theoretical considerations are explored regarding the impact of trauma symptoms on vicarious post- traumatic growth. The clinical implications underscore the importance of identifying elevated levels of secondary traumatic stress and emphasize the necessity for interventions aimed at reducing stress levels while simultaneously fostering an increase in vicarious post-traumatic growth. |
| 47 | Woolhouse | 2012 | "Building through the grief": Vicarious trauma in a group of inner-city family physicians | Cross sectional | NA | 20 | This qualitative investigation aimed to delve into the encounters of family doctors who provided care for women using illegal substances. | Ten family doctors practicing in Toronto and Ottawa, Canada, took part in extensive interviews. The information gathered was examined through a method involving an interactive and interpretive analysis. | The primary focus derived from the data analysis  was the emotional toll of their profession. Participants revealed the emotional struggles, moments of grief, and instances of happiness while navigating patient care. The underlying sub- themes encompassed tragedies and loss, challenging patient behaviors, and feeling disconnected from the broader medical community.  Additionally, a second significant theme revolved around coping mechanisms. Participants openly and thoughtfully discussed three key strategies: adjusting and refining their practice approach, fostering teamwork, and revising their expectations as a means of coping. | The stories shared by the participants, revolving around experiences of loss, grief, and compassion, align with indicators of vicarious trauma.  Consequently, participants faced the potential risk of developing compassion fatigue, which is a distinct manifestation of burnout. These revelations represent novel and significant discoveries. It's crucial to conduct additional research to delve deeper into the correlation between vicarious trauma and the emergence of burnout specifically among family physicians. |
| 48 | Nielsen, H. G. | 2009 | Preventing burnout among general practitioners: is there a possible route? | Qualitative research | NA | 17 | This study aims to provide a detailed observational account, presented in the form of a case study, of the strategies employed to mitigate burnout among General Practitioners (GPs). | The study examined a supervision group formed in 2000 with nine GPs, led by an experienced supervisor. Monthly sessions aimed to enhance reflective practices, increase awareness of occupational conditions, and address compassion fatigue. Various methods, including the reflecting team model, were employed. Qualitative research methods, such as interviews and participant observation, were used over three sessions. Analysis followed a phenomenological-hermeneutic tradition using NVivo software. Results provide insights into burnout prevention methods for GPs in a group setting. | All GPs in the supervision group found it beneficial, leading to changes in patient interactions and daily work organization. The support prevented two doctors from considering job changes due to challenging working conditions. GPs shifted from constant troubleshooting to a more reflective, patient-centered approach. The supervisor stressed the importance of slowing down and adopting a holistic perspective. The study addressed challenges faced by self-employed GPs and explored the broader concept of emotional exhaustion, termed "compassion fatigue," in the context of challenging patient encounters. | Current research on supervision has mainly  focused on fields other than General Practitioners (GPs) and often involved one-to-one settings.  However, Balint groups, a form of group supervision, have been extensively studied globally, demonstrating positive effects on doctors' patient- centeredness and job satisfaction. This study examines experienced GPs in a supervision group for over five years, using qualitative methods to capture self-reported changes. The findings emphasize the importance of reflection in supervision, address the impact of working conditions, and introduce the concept of "compassion fatigue" in the context of GPs facing frequent and challenging patient encounters. Group supervision is suggested as a preventive measure against stress and burnout, particularly in the early professional life of GPs, urging further research in longitudinal prospective studies on its burnout prevention contribution. |
| 49 | Aase, M. | 2008 | "If you cannot tolerate that risk, you should never become a physician": a qualitative study about existential experiences among physicians | Cross sectional | NA | 18 | Doctors frequently encounter existential concerns at their workplace, yet there's limited understanding regarding how these matters personally affect them. | A qualitative study was conducted using individual  semi-structured interviews. The analysis involved examining accounts of their experiences in dealing with these challenges. The methodology employed a systematic approach called systematic text condensation, which included several stages:  Initial immersion in the material to grasp an overall understanding while setting aside any preconceived notions.  Identifying meaningful segments representing various aspects of the physicians' experiences and assigning codes to these segments.  Condensing and abstracting the essence within each code group.  Summarizing the contents of each code group to create generalized descriptions and concepts that encapsulate the most significant experiences reported by the participants.  This analysis unfolded in a step-by-step manner, with new interviews complementing the existing sample. The approach used was an editing analysis style, following Crabtree and Miller's methodology, where categories were derived directly from the collected data instead of being guided by a predetermined theoretical framework. | The participants recounted a progression in their ability to manage the existential challenges in their work. Initially, encounters perceived as distressing or traumatic were deeply affecting, but over time, they developed a capacity to navigate such experiences, particularly those involving death, and maintain a sense of detachment. Vulnerability was intertwined with their professional duties and identity, viewed as a weight they needed to manage. However, this responsibility was counterbalanced by a sense of purpose derived from their work, stemming from the impact they made in their patients' lives. Being part of a community of colleagues was deemed essential for handling the isolation and sense of powerlessness associated with their vulnerable professional standing. | The vulnerability experienced by physicians when confronted with life-and-death situations has been undervalued. Being part of supportive and compassionate communities can aid in both personal growth and professional identity. It also aids in coping when exposed to the existential aspects inherent in clinical work. |
| 50 | Ruiz- Fernández | 2020 | Compassion fatigue, burnout, compassion satisfaction and perceived stress in healthcare professionals during the COVID-19 health crisis in Spain | Cross sectional | 11 | NA | The aim of this study was to assess the levels of compassion fatigue (CF), burnout (BO), compassion satisfaction (CS), and perceived stress among healthcare professionals during the COVID-19 health crisis in Spain. Spain has faced significant challenges due to the pandemic, placing healthcare workers in traumatic and stressful work environments.  These conditions may lead to the development of CF or BO among professionals, increasing the risk of mental health issues. | The study involved 506 healthcare professionals,  including physicians and nurses, actively working during the peak of the COVID-19 pandemic in Spain. The professionals participated in an assessment of compassion fatigue (CF), compassion satisfaction (CS), and burnout (BO) using the Professional Quality of Life Questionnaire. Perceived stress was also evaluated using the Perceived Stress Scale-14. The study considered socio-demographic and occupational variables, following the STROBE guidelines for observational study reporting. The data collection period aligned with the highest incidence of COVID-19 cases and mortality rates in Spain. | Physicians exhibited elevated scores in both compassion fatigue (CF) and burnout (BO), whereas nurses reported higher scores in compassion satisfaction (CS). Perceived stress levels were similar between the two professional groups. Those working in specialized COVID-19 units and emergency departments displayed increased CF and BO scores, while CS levels and perceived stress remained consistent across different workplaces. | Despite the challenging health crisis and its impact on healthcare professionals, moderate to high levels of compassion fatigue (CF) and burnout (BO) persist. However, there appears to be an upward trend in compassion satisfaction (CS), particularly among nurses. This could be attributed to their dedication to alleviating suffering and the recognition they perceive socially. Implementing interventions to enhance CS and prevent BO and CF in the long run is essential for the well-being of these professionals. |
| 51 | Jordan Teel, | 2019 | Secondary traumatic stress among physiatrists treating trauma patients | Cross- sectional study | 14 | NA | Secondary traumatic stress is a variant of posttraumatic stress disorder (PTSD) that emerges from exposure to the acute serious physical harm or death experienced by others, irrespective of the specific cause. Despite this, the occurrence of secondary traumatic stress among physiatrists has not been thoroughly investigated. This study aims to explore the prevalence and factors associated with secondary traumatic stress among physiatrists. | The survey was disseminated to members of the Association of Academic Physiatry and local physiatrists. The surveys encompassed assessments of secondary traumatic stress, resilience, personality factors, demographics, and work-related factors. | Out of the 102 surveys received, 88 were fully  completed and considered for analysis. The sample had an average age of 42 ± 11 years, with 45 women (51%). The study revealed that 30% of respondents experienced moderate to severe levels of secondary traumatic stress, and 45% reported clinical levels in at least one symptom cluster. Notably, individuals with higher resilience, higher extraversion, and higher emotional stability demonstrated significantly lower odds of positive screens for secondary traumatic stress and lower symptom severity (all P < 0.023). | In summary, the findings indicate that one-third of participating physiatrists reported experiencing moderate to severe symptoms of secondary traumatic stress. This rate aligns with previous research conducted among clinicians in trauma settings and exceeds the prevalence of posttraumatic stress disorder in the general population. Implementing interventions focused on building resilience is likely to enhance the well- being of physiatrists facing secondary traumatic stress. |
| 52 | Behrani, Aliza | 2020 | The Relationship between Empathy, Coping Strategies, and Compassion Fatigue in Doctors | Cross- Sectional Study | 12 | NA | This study aims to find out about the  correlation between empathy and coping; the correlation between coping and compassion fatigue; and the correlation between empathy and compassion fatigue, in physicians.  Currently, there are some existing studies that have been done to explore compassion fatigue. However, these are mainly studies done in the western world. There is a lack of research done on compassion fatigue in Pakistan, especially with respect to doctors. There is also a lack of current data and studies which explore the relationship between empathy, coping strategies, and compassion fatigue. Findings from this study can be useful in forming interventions such as improving coping skills, to reduce compassion fatigue and to prevent diminishing levels of empathy in doctors.  Based on the current literature, the researchers formulated the following hypotheses: 1) There will be a significant relationship between empathy and avoidance- focused coping. 2) There will be a significant relationship between empathy and approach- focused coping. 3) There will be a significant positive relationship between avoidance- focused coping and compassion fatigue. 4) There will be a significant negative relationship between approach-focused coping and compassion fatigue. 5) There will be a significant positive relationship between  empathy and compassion fatigue. | Participants were selected through purposive sampling, from multiple hospitals and clinics as this researchers wanted to study empathy, compassion fatigue and coping in physicians who have in-person encounters with patients. Other inclusion criteria was for the physicians to be MBBS graduates, who were employed in a private or public healthcare setting in Pakistan during the time of data collection and who treats patients on a daily basis. The participant group consisted of 153 practicing doctors, including 52 males and 101 females, with ages ranging from 22 to 70 years. | The researchers found that doctors exhibit different levels of traumatic stress when caring for ailing patients, irrespective of their years of experience. Avoidance coping was found to be greatly associated with presence of compassion fatigue, whereas avoidance coping and approach coping were found to be less significant in predicting compassion fatigue. However since there is still an association, the study suggests employing coping methods to help decrease incidence of compassion fatigue. | There is a need to raise awareness on stress management and compassion fatigue.  Consequently, it is recommended to implement interventions and programs to raise awareness amongst healthcare professionals and hospital management staff. This can help enhance knowledge regarding adaptive stress-coping strategies and relevant policies to aid in preventing burnout and compassion fatigue. |
| 53 | Allie | 2018 | Bereavement overload and its effects on, and related coping mechanisms of health care providers and ward administrators at National District Hospital in Bloemfontein, Free State | Cross- sectional study | 7 | NA | Patient death is an inevitable experience for healthcare workers, and those working directly with patients and their families often bear significant emotional strain. The concept of bereavement overload, characterized by multiple losses without sufficient time for normal grief, is a phenomenon of interest. The study aims to investigate bereavement overload, exploring its effects and the coping mechanisms employed by personnel working in adult medical wards. | The study employed an analytical cross-sectional design, utilizing an interviewer-administered questionnaire. The target population consisted of health care providers, including 13 doctors and 20 nurses, as well as eight final-year medical students and four administrative staff. The study was conducted in the four adult medical wards at National District Hospital from August to October 2016. | Out of the 45 participants, almost half (48.9%) experienced bereavement overload. No medical students reported bereavement overload, while percentages were higher among nurses (60.0%), administrative staff (75.0%), and doctors (53.9%). A significant majority (64.5%, n = 29) acknowledged suffering from compassion fatigue. Regarding coping mechanisms, 62.2% employed only positive strategies, with the use of negative coping mechanisms being directly associated with a longer duration in the medical field. | It is crucial to establish support systems to address the 49% prevalence of bereavement overload and prevent the consequences of negative coping mechanisms. The goal is for healthcare providers grappling with bereavement overload to attain compassion satisfaction and deepen their commitment to patients' well-being without compromising their own. |
| 54 | Nicola McKinley | 2020 | Resilience, burnout and coping mechanisms in UK doctors: a cross-sectional study | Cross- sectional study | 15 | NA | This cross-sectional study sought to evaluate the resilience, professional quality of life, and coping strategies among doctors in the UK. Additionally, it aimed to examine how demographic variables, including gender, professional grade, and specialty, influence these aspects. | In October and November 2018, medical practitioners in the UK were invited to participate in an online survey comprising validated psychological instruments. The invitation to take part was extended by Royal Colleges and other medical organizations through newsletters, email invitations, websites, and social media channels. | A total of 1651 doctors, spanning various  specialties and grades across the UK, participated in the study. The average resilience score was  65.01 (SD 12.3), which was lower than the norms observed in the general population. Among the respondents, 31.5% experienced high burnout (BO), 26.2% reported high secondary traumatic stress, and 30.7% had low levels of compassion satisfaction (CS). Notably, doctors in emergency medicine exhibited higher levels of burnout compared to other specialties (F=2.62, p=0.001, df 14), while those in general practice had the lowest scores for CS (F=6.43, p<0.001, df 14). A total of 120 doctors (8%) met the criteria for simultaneously high burnout, high secondary traumatic stress, and low compassion satisfaction. The most frequently cited coping mechanism was the maladaptive strategy of self-distraction. | Thirty-three percent of the UK doctors who participated in the survey are experiencing burnout and secondary traumatic stress (STS). Particularly, respondents from emergency medicine and general practice seem to be facing the highest levels of these challenges. More than 100 doctors are categorized as being at risk due to simultaneously high burnout (BO), high secondary traumatic stress (STS), and low compassion satisfaction (CS).  Analyzing the free-text responses from doctors in future studies may provide insights into the factors contributing to the elevated levels of burnout and STS reported by medical professionals. |
| 55 | Tan, S. B. et al. | 2020 | The experiences of well-being of palliative care providers in Malaysia: A thematic analysis | Qualitative Interview Study | NA | 16 | To explore the experiences of well-being among palliative care providers in Malaysia. | Qualitative study using semi-structured interviews. Thematic analysis of data from 18 palliative care providers (9 doctors and 9 nurses). | Identified themes include 1) values and strengths; 2) coping and work-life balance; 3) social support and spirituality; 4) passion and satisfaction; and 5) challenges and stressors. | Palliative care providers derive meaning and satisfaction from their work despite challenges. This study emphasizes the importance of coping strategies, social support, and spirituality in well-being. |
| 56 | Haber, Y. et al. | 2013 | Predictors of professional quality of life among physicians in a conflict setting: The role of risk and protective factors | Cross-sectional study | 10 | NA | Unlike other places in the western world, Israeli physicians are prone to be exposed to victims of terror and war (soldiers and civilians alike), while in some cases the patients are close friends or relatives. Moreover, in other armed conflict situations (stemming from war or terror), there is a direct threat to the physician's life and his/her family. Among hospital personnel, there is little research concerning the factors associated with aspects of professional quality of life such as burnout, compassion fatigue, and compassion satisfaction. The current study compared a set of risk and protective factors associated with burnout, compassion fatigue, and compassion satisfaction. | The sample consisted of 97 physicians who answered a wide battery of questionnaires tapping to the aforementioned factors that served as predictive variables (age, gender, marital status, PTSD symptoms, depressive symptoms, dissociative symptoms, life satisfaction, perceived self-efficacy, perceived family support) using multiple regressions. | The study results showed that higher levels of PTSD symptoms were associated with higher levels of compassion fatigue (β = .594; t = 4.419; p <.001). A higher level of life satisfaction was associated with lower burnout (β = -.436; t = -4.293; p <.001). The same results were found also in lower level of perceived family support (β = -.203; t = -2.533; p <.05), and higher level of perceived self-efficacy was associated with higher burnout (β = .298; t = 2.702; p <.01). Finally, a higher level of life satisfaction was associated with higher compassion satisfaction (β = .493; t = 4.419; p <.001). | These results may suggest that life satisfaction is a predictor associated with burnout and compassion satisfaction. These results are viewed in light of the importance of life satisfaction as a barrier against burnout and its implication for physicians and hospital policy. |
| 57 | Carmassi, C. et al. | 2022 | Gender and occupational role differences in work-related post-traumatic stress symptoms, burnout and global functioning in emergency healthcare workers | Cross-sectional study | 13 | NA | To explore gender and occupational role impact on work-related Post-Traumatic Stress Symptoms, Post-Traumatic Stress Disorder, burnout and global functioning in a sample of emergency healthcare workers. | 126 healthcare workers of the Emergency Department, including Intensive Care Unit, Emergency Room and Emergency Medicine, of a major University Hospital in central Italy were recruited. Participants were assessed by means of the: Trauma and Loss Spectrum-Self Report (TALS-SR) to explore Post-Traumatic Stress Spectrum Symptoms, Professional Quality of Life (ProQOL) Scale to assess Compassion Satisfaction, Burnout and Compassion Fatigue and Work and Social Adjustment Scale (WSAS) to measure global functioning. | The present findings showed females were more prone to develop Post-Traumatic Stress Symptoms, particularly re-experiencing (p = .010) and hyperarousal (p = .026) symptoms and medical doctors reporting higher Burnout (p < .001) and lower Compassion Satisfaction (p = .009) mean scores than nurses. Higher levels of functioning impairment emerged amongst medical doctors rather than nurses, in both social (p = .029) and private (p = .020) leisure activities. Linear correlations highlighted relationships between the TALS-SR, ProQOL and WSAS scores. Finally, medical doctor status was significantly associated with lower Compassion Satisfaction (p = .029) and higher Burnout (p = .015). | Results highlight high post-traumatic stress symptoms and burnout levels in emergency healthcare workers with a relevant impact of female gender and occupational role, supporting the need for preventive strategies, also in light of the current COVID-19 pandemic. |
| 58 | Boateng | 2019 | Elisabeth Kübler-Ross as Astrophysicist: Emotional Intelligence and Resilience Unlock the Black Hole of Physician Burnout, Moral Distress, and  Compassion Fatigue | Report | NA | NA | The aim of the paper is to explore the emotional challenges faced by physicians in the context of end-of-life care, particularly focusing on the impact of physician burnout, moral distress, and compassion fatigue. The document aims to highlight the interconnectedness of these emotional burdens and their potential implications for the quality of patient care. Additionally, the paper seeks to emphasize the importance of emotional intelligence and resilience in addressing these challenges and promoting effective communication and decision-making in healthcare settings. | qualitative methodology: It draws on a range of sources, including literature reviews, clinical observations, and commentary from experienced healthcare professionals to provide a comprehensive understanding of the emotional challenges faced by physicians in caring for seriously ill and dying patients. The methodology involves synthesizing existing research, theoretical frameworks, and practical insights to present a nuanced analysis of the impact of emotional burden on healthcare professionals and the strategies to promote emotional intelligence and resilience in this context. | This paper highlights the significant impact of emotional burden on healthcare professionals, particularly in the context of caring for seriously ill and dying patients. It emphasizes the interconnectedness of physician burnout, moral distress, and compassion fatigue, and their potential to compromise the quality of patient care. The document also underscores the importance of emotional intelligence and resilience in addressing these challenges, promoting effective communication, and enhancing decision-making processes in healthcare settings. | We propose that recognizing and truly  understanding the individual beyond the array of symptoms can enhance clinician job satisfaction and resilience. This involves a profound awareness of one's emotions, enabling the redirection of potentially maladaptive feelings and creating a safe space for shared experiences. This emotional intelligence comprises four principles: perceiving emotions, using emotions, understanding emotions, and managing emotions. Integrating this emotional intelligence into medical practice improves interpersonal communication, judgment, and personal clarity in decision-making. Especially when caring for seriously ill or dying patients, clinicians must acknowledge, address, and navigate their own, the patient's, and the patient's family's intense and often conflicting emotions. Self- awareness skills that foster emotional intelligence include naming feelings, accepting their normalcy, and reflecting on emotions. Evidence indicates that individuals with high emotional intelligence exhibit higher levels of job performance across diverse domains. This emotional intelligence further fosters, if not engenders, resilience – the mechanism many physicians adopt to reconcile managing emotions while navigating difficult decision-making. |
| 59 | Malik, H. | 2022 | The impact of mindfulness practice on physician burnout: A scoping review | Scoping Review | NA | NA | Burnout amongst physicians is becoming exceedingly common within the healthcare system internationally, especially in the context of COVID-19. Increasing emotional exhaustion, depersonalization, and reduced personal accomplishment can detriment the quality of healthcare. Compassion fatigue and moral injury can also harm the doctor-patient relationship. This study recommends mindfulness practice as a possible intervention to prevent and reduce the occurrence of such issues. Mindfulness practice has been proven to be able reduce burnout, increase compassion and prevent moral injury in physicians. | This study is a scoping review which aims to find out the impacts of mindfulness practice on burnout amongst physicians. This search focused on studies published by well acclaimed journals, to ensure the quality of these studies. The search also included studies carried out on current practicing physicians, to ensure the studies selected were relevant in today's context. The interventions considered were mainly on various types of mindfulness practice. This review excludes studies and gray literature carried out only on allied healthcare professionals. | This scoping review incorporated a total of 31 studies. Mindfulness practice demonstrated a reduction in emotional exhaustion and depersonalization, along with improvements in mood, stress responses, and vigor among participants. Additionally, the studies revealed increased levels of self-awareness, compassion, and empathy. Based on this review, there were four overarching themes that were identified.  These include mindfulness and positive psychology, new methods in mindfulness practice, identification of barriers and facilitators to physician mindfulness practice and the effect of mindfulness on work and patient care. | Though the reviewed studies commonly highlighted the positive impact of mindfulness on mental health and well-being, they appeared to have a narrow focus which did not take into account the effects of mindfulness practice on healthcare leadership, organizational culture, and practices. Mindfulness practice is integral to positive psychology and is correlated to improving leadership. Physician leaders who are mindful and compassionate can significantly contribute to dealing with current problems in healthcare, taking into account the importance of mental health of staff, and fostering a supportive environment for innovation. |
| 60 | McCain, R. S. et al. | 2017 | A study of the relationship between resilience, burnout and coping strategies in doctors | Cross-sectional study | 10 | NA | The aim of this study was to measure resilience, coping and professional quality of life in doctors. | A cross-sectional study using an online questionnaire in a single National Health Service trust, including both primary and secondary care doctors. | 283 doctors were included. Mean resilience was 68.9, higher than population norms. 100 (37%) doctors had high burnout, 194 (72%) doctors had high secondary traumatic stress and 64 (24%) had low compassion satisfaction. Burnout was positively associated with low resilience, low compassion satisfaction, high secondary traumatic stress and more frequent use of maladaptive coping mechanisms, including self-blame, behavioural disengagement and substance use. Non-clinical issues in the workplace were the main factor perceived to cause low resilience in doctors. | Despite high levels of resilience, doctors had high levels of burnout and secondary traumatic stress. Doctors suffering from burnout were more likely to use maladaptive coping mechanisms. As doctors already have high resilience, improving personal resilience further may not offer much benefit to professional quality of life. A national study of professional Quality of Life, Coping And REsilience, which we are proposing to undertake, will for the first time assess the UK and Ireland medical workforce in this regard and guide future targeted interventions to improve professional quality of life. |
| 61 | Pruthi | 2014 | Doctors do cry | Report | NA | NA | This article explores the emotional experiences of physicians, particularly focusing on the act of crying in response to patient suffering or death. It examines whether such emotional expressions are perceived as a lack of professional control or as a demonstration of humanity and empathy. | The methodology used in the paper is to patient care primarily based on narrative analysis and the compilation of existing research on the topic of doctors' emotional responses, particularly crying. The authors also of medical students draw on personal and physicians to experiences and observations explore the emotional to support their arguments. | Both medical students and experienced physicians do cry when overwhelmed by stress and emotions, contradicting the belief that doctors should remain detached. The study emphasizes the lack of emotional training in the medical curriculum, which focuses on disease treatment but not on dealing with death, grief, and emotional situations. | These findings shed light on the emotional experiences of medical professionals and the need for a more comprehensive approach to emotional training in medical education. |
| 62 | Rabin, S. et al. | 2009 | Balint groups as 'shared care' in the area of mental health in primary medicine | Perspective | NA | NA | To describe how Balint groups can be effective for primary care doctors and facilitate dialogue between mental health professionals and primary care physicians. | Descriptive analysis of Balint group activities and discussion on the role of group leaders in interdisciplinary learning. | Balint groups with joint leadership of professionals from different disciplines can be an effective method to improve primary care and mental health cooperation. Balint groups can facilitate dialogue and improve cooperation between mental health professionals and primary care physicians. Balint groups led by a mix of primary care and mental health professionals can help generate new interdisciplinary perspectives and relieve emotional burden for the presenting physician. | Balint groups are effective in supporting primary care doctors and promote shared care in mental health through facilitated dialogue |
| 63 | Kegye, A. | 2016 | How does the suffering of cancer patients affect us? Reviewing the physical and mental wellbeing of hospice workers | Systematic Review | NA | NA | In Hungary, the negative physical and mental effects due to occupational stress in hospice workers is becoming more prevalent. The development of physical, psychological, and social symptoms is noted as a factor that may contribute to the challenges experienced by caregivers, potentially leading to a fluctuating workforce. The researchers aim to be able to help to address the well-being of hospice workers and reduce the occurrence of physical and mental fatigue amongst them. | A comprehensive literature search was conducted across international databases to identify pertinent studies on the physical and mental state of health care workers who deal primarily with terminally ill patients, particularly palliative and hospice care workers. Inclusion criteria were articles that were published between 2000 and 2013 that were written in the English language. | 40 articles met the inclusion criteria. Of the 40, 16 articles were chosen for the systemic review, as the other articles focused only on a particular area of physical and mental fatigue. While reviewing the available literature, researchers were also able to carefully examine the emotional wellbeing and coping strategies of hospice workers. Researchers found that a noteworthy addition to recent research is the exploration of the relationship between compassion satisfaction, compassion fatigue, and burnout, symptoms of these and coping strategies. | The literature review enhanced understanding of the causes of negative physical and mental fatigue and serves as a foundation for developing more effective methods aimed at preventing such issues amongst hospice professionals. |
| 64 | Dale, S. and Olds, J. | 2012 | Maintaining professionalism in the face of burnout | Commentary | NA | NA | To discuss the challenges of maintaining professionalism amid burnout and the associated risks | Discussion based on existing literature and professional experiences. | Doctors are able to maintain professional levels of care for patients despite experiencing depersonalization and other symptoms of burnout. Nearly half of UK GPs report experiencing emotional exhaustion, depersonalization, and low levels of personal accomplishment, indicating high rates of burnout. The high standards of professionalism expected of doctors can contribute to a form of institutionalized perfectionism that may lead to burnout. Burnout can lead to serious errors, underperformance, or sudden absence. There is pressure to maintain professionalism may cause individuals to hide concerns or mistakes, increasing risk. | Organizations should address burnout to prevent risks associated with compromised professionalism. Open communication is encouraged to address concerns and mistakes. |
| 65 | Lee, F. J. et al. | 2008 | Stress, burnout, and strategies for reducing them: What's the situation among Canadian family physicians? | Cross sectional survey | 12 | NA | To ascertain Canadian family physicians' levels of stress and burnout and the strategies they use to reduce these problems. | Design: Census survey.  Setting: Kitchener-Waterloo, an urban area with a population of approximately 300 000 in southwestern Ontario.  Participants: Family physicians.  Main outcome measures: Scores on the Family Physician Stress Inventory, scores on strategies to reduce personal stress, scores on strategies to reduce stress on the job, and scores on the Maslach Burnout Inventory. | Participation rate was 77.8% (123 of 158 surveys returned). About 42.5% of participants had high stress levels. Burnout was defined by 3 components: emotional exhaustion, depersonalization (going through the day like an "automaton"), and perceived lack of personal accomplishment. Many respondents scored high on the burnout inventory, and almost half had high levels of emotional exhaustion and depersonalization (47.9% and 46.3%, respectively). No demographic factors were associated with high scores on these components. Use of strategies to reduce personal and occupational stress was associated with lower levels of burnout. Scores on the Family Physician Stress Inventory correlated highly with scores on the Maslach Burnout Inventory. | Regardless of demographic factors, family physicians are at risk of having high levels of stress and burnout. Classic burnout is related to stress brought on by factors such as too much paperwork, long waits for specialists and tests, feeling undervalued, feeling unsupported, and having to abide by rules and regulations. Common strategies for reducing personal stress included eating nutritiously and spending time with family and friends. Common strategies for reducing stress on the job included valuing relationships with patients and participating in continuing medical education. Stress and burnout are related to the desire to give up practice and are, therefore, a human resources issue for the entire health care system. |
| 66 | Tan, S. B. | 2013 | The experiences of Stress of Palliative Care Providers in Malaysia: A thematic analysis | Cross- Sectional Study | NA | 20 | This is a qualitative study which was carried  out to find out more about the stress experiences of 20 palliative care providers at the University Malaya Medical Centre in Malaysia, using semi-structured interviews. Several themes were identified from the results, namely 9 main themes: (1) organizational challenges, (2) care overload,  (3) communication challenges, (4) differences in opinion, (5) misperceptions and misconceptions, (6) personal expectations, (7) emotional involvement, (8) thoughts on death and dying, and (9) appraisal and coping. Based on this, this study offers a comprehensive care model to target stress faced by palliative care providers due to work exposure. This model may offer insights in  developing methods aimed to prevent and manage stress in palliative care settings. | The participants for this study were selected by convenience sampling and gave informed consent. 10 doctors and 10 nurses underwent semistructured interviews conducted by a principal investigator, with the use of a voice recorder and a list of questions (Table 1). The 8 questions were curated based on the biopsychosocial–spiritual model of suffering. | The results were classified into 9 primary themes:  (1) organizational challenges, (2) care overload,  (3) communication challenges, (4) differences in opinion, (5) misperceptions and misconcep- tions,  (6) personal expectations, (7) emotional involvement, (8) death and dying thoughts, and (9) appraisal and coping. | Occupational stress in palliative care providers can be derived from 2 main causes, that is; difficulties in caring for others and problems with caring for themselves. This study recommends a three- pronged method targeting work factors (organizational challenges and care overload), interpersonal factors (communication challenges, differences in opinion, and misperceptions– misconceptions), and personal factors (personal expectations, emotional involvement, and death and dying thoughts). |
| 67 | Sood, A. et al. | 2011 | Stress management and resilience training among department of medicine faculty: A pilot randomized clinical trial | Poster Presentation of Randomised control trial | 8 | NA | Physician distress is common and related to numerous factors involving physicians' personal and professional lives. The present study was designed to assess the effect of a Stress Management and Resiliency Training (SMART) program for increasing resiliency and quality of life, and decreasing stress and anxiety among Department of Medicine (DOM) physicians at a tertiary care medical center. | Forty DOM physicians were randomized in a wait-list controlled clinical trial to either the SMART intervention or a wait-list control group for 8 weeks. The intervention involved a single 90 min one-on-one training in the SMART program. Primary outcome measures assessed at baseline and week 8 included the Connor Davidson Resilience Scale (CDRS), Perceived Stress Scale (PSS), Smith Anxiety Scale (SAS) and Linear Analog Self Assessment Scale (LASA). | Thirty-two physicians completed the study. A statistically significant improvement in resiliency, perceived stress, anxiety, and overall quality of life at 8 weeks was observed in the study arm compared to the wait-list control arm: CDRS: mean ± SD change from baseline +9.8 ± 9.6 vs. -0.8 ± 8.2, t(30) = 3.18, p = 0.003; PSS: -5.4 ± 8.1 vs. +2.2 ± 6.1, t(30) = -2.76, p = 0.010; SAS: -11.8 ± 12.3 vs.+ 2.9 ± 8.9, t(30) = -3.62, p = 0.001; and LASA: +0.4 ± 1.4 vs. -0.6 ± 1.0, t(30) = 2.29, p = 0.029. | A brief training to enhance resilience and decrease stress among physicians using the SMART program was feasible. Further, the intervention provided statistically significant improvement in resilience, stress, anxiety, and overall quality of life. In the future, larger clinical trials with longer follow-up and possibly wider dissemination of this intervention are warranted. |
| 68 | Sood, A. et al. | 2014 | Stress management and resiliency training (SMART) program among department of radiology faculty: A pilot randomized clinical trial | Pilot randomized clinical trial | 12 | NA | To test the efficacy of a Stress Management and Resiliency Training (SMART) program for decreasing stress and anxiety and improving resilience and quality of life among Department of Radiology physicians. | The study was approved by the institutional review board. A total of 26 Department of Radiology physicians were randomized in a single-blind trial to either the SMART program or a wait-list control arm for 12 weeks. The program involved a single 90-min group session in the SMART training with two follow-up phone calls. Primary outcomes measured at baseline and week 12 included the Perceived Stress Scale, Linear Analog Self-Assessment Scale, Mindful Attention Awareness Scale, and Connor-Davidson Resilience Scale. | A total of 22 physicians completed the study. A statistically significant improvement in perceived stress, anxiety, quality of life, and mindfulness at 12 weeks was observed in the study arm compared to the wait-list control arm; resilience also improved in the active arm, but the changes were not statistically significant when compared to the control arm. | A single session to decrease stress among radiologists using the SMART program is feasible. Furthermore, the intervention afforded statistically significant and clinically meaningful improvement in anxiety, stress, quality of life, and mindful attention. Further studies including larger sample size and longer follow-up are warranted. |
| 69 | Wallace, J. E. | 2009 | Physician wellness: A missing quality indicator | Literature Review | NA | NA | To argue that health systems should routinely measure physician wellness and discuss implementation challenges. | The authors conducted a literature review, searching Medline and the Cochrane Library for relevant articles published between 1985-2009. They focused on reports published in the past 5 years, but also included highly regarded older studies. The authors selected a subset of high-quality studies that they judged to be most effective and relevant to support the central themes of the review. | Physician wellness is an important indicator of health system quality, as physician distress can negatively impact health system performance. Many physicians face significant work-related stresses and high rates of burnout, up to 75% in some studies. Physician wellness is influenced by individual, professional, and organizational factors, with the medical culture often deterring physicians from seeking care for themselves. | This study recommends routine measurement of physician wellness as a quality indicator and highlights the need to overcome barriers to implementing wellness initiatives. |
| 70 | Krasner, M. S. | 2009 | Association of an educational program in mindful communication with burnout, empathy, and attitudes among primary care physicians | Quantitative interventional study, single group | 11.5 | NA | Primary care physicians report high levels of distress, which is linked to burnout, attrition, and poorer quality of care. Programs to reduce burnout before it results in impairment are rare; data on these programs are scarce. The objective is to determine whether an intensive educational program in mindfulness, communication, and self-awareness is associated with improvement in primary care physicians' well-being, psychological distress, burnout, and capacity for relating to patients. | Before-and-after study of 70 primary care physicians in Rochester, New York, in a continuing medical education (CME) course in 2007-2008. The course included mindfulness meditation, self-awareness exercises, narratives about meaningful clinical experiences, appreciative interviews, didactic material, and discussion. An 8-week intensive phase (2.5 h/wk, 7-hour retreat) was followed by a 10-month maintenance phase (2.5 h/mo). Main outcome measures include Mindfulness (2 subscales), burnout (3 subscales), empathy (3 subscales), psychosocial orientation, personality (5 factors), and mood (6 subscales) measured at baseline and at 2, 12, and 15 months. | Over the course of the program and follow-up, participants demonstrated improvements in mindfulness (raw score, 45.2 to 54.1; raw score change [Delta], 8.9; 95% confidence interval [CI], 7.0 to 10.8); burnout (emotional exhaustion, 26.8 to 20.0; Delta = -6.8; 95% CI, -4.8 to -8.8; depersonalization, 8.4 to 5.9; Delta = -2.5; 95% CI, -1.4 to -3.6; and personal accomplishment, 40.2 to 42.6; Delta = 2.4; 95% CI, 1.2 to 3.6); empathy (116.6 to 121.2; Delta = 4.6; 95% CI, 2.2 to 7.0); physician belief scale (76.7 to 72.6; Delta = -4.1; 95% CI, -1.8 to -6.4); total mood disturbance (33.2 to 16.1; Delta = -17.1; 95% CI, -11 to -23.2), and personality (conscientiousness, 6.5 to 6.8; Delta = 0.3; 95% CI, 0.1 to 5 and emotional stability, 6.1 to 6.6; Delta = 0.5; 95% CI, 0.3 to 0.7). Improvements in mindfulness were correlated with improvements in total mood disturbance (r = -0.39, P < .001), perspective taking subscale of physician empathy (r = 0.31, P < .001), burnout (emotional exhaustion and personal accomplishment subscales, r = -0.32 and 0.33, respectively; P < .001), and personality factors (conscientiousness and emotional stability, r = 0.29 and 0.25, respectively; P < .001). | Participation in a mindful communication program was associated with short-term and sustained improvements in well-being and attitudes associated with patient-centered care. Because before-and-after designs limit inferences about intervention effects, these findings warrant randomized trials involving a variety of practicing physicians. |
| 71 | Shapiro, S. L. et al. | 2005 | Mindfulness-based stress reduction for health care professionals: Results from a randomized trial | Randomised controlled pilot study | 12 | NA | To examine the effectiveness of an 8-week Mindfulness-Based Stress Reduction (MBSR) program in reducing stress and improving quality of life among health care professionals. | Prospective randomized controlled pilot study was conducted. Participants underwent an 8-week MBSR intervention. Measures of stress, quality of life, and self-compassion were assessed. | The MBSR intervention led to significant reductions in perceived stress and increases in self-compassion compared to the control group. The majority of participants in the MBSR group showed improvements in stress, self-compassion, life satisfaction, job burnout, and psychological distress. | This study suggests that MBSR programs can be beneficial for health care professionals and recommends further research and practice implications. |
| 72 | Beckman, H. B. et al. | 2012 | The impact of a program in mindful communication on primary care physicians | Qualitative Interview Study | NA | 18 | In addition to structural transformations, deeper changes are needed to enhance physicians' sense of meaning and satisfaction with their work and their ability to respond creatively to a dynamically changing practice environment. The purpose of this research was to understand what aspects of a successful continuing education program in mindful communication contributed to physicians' well-being and the care they provide. | In 2008, the authors conducted in-depth, semistructured interviews with primary care physicians who had recently completed a 52-hour mindful communication program demonstrated to reduce psychological distress and burnout while improving empathy. Interviews with a random sample of 20 of the 46 physicians in the Rochester, New York, area who attended at least four of eight weekly sessions and four of eight monthly sessions were audio-recorded, transcribed, and analyzed qualitatively. The authors identified salient themes from the interviews. | Participants reported three main themes: (1) sharing personal experiences from medical practice with colleagues reduced professional isolation, (2) mindfulness skills improved the participants' ability to be attentive and listen deeply to patients' concerns, respond to patients more effectively, and develop adaptive reserve, and (3) developing greater self-awareness was positive and transformative, yet participants struggled to give themselves permission to attend to their own personal growth. | Interventions to improve the quality of primary care practice and practitioner well-being should promote a sense of community, specific mindfulness skills, and permission and time devoted to personal growth. |
| 73 | Hick, S. F. and Bien, T. | 2008 | Mindfulness and the therapeutic relationship | Book | NA | NA | This book focuses specifically on how mindfulness can deepen the therapeutic relationship. Grounded in research, chapters demonstrate how therapists' own mindfulness practice can help them to listen more attentively and be more fully present. Leading proponents of different treatment approaches—including behavioral, psychodynamic, and family systems perspectives—illustrate a variety of ways that mindfulness principles can complement standard techniques and improve outcomes by strengthening the connection between therapist and client. Also presented are practical strategies for integrating mindfulness into clinical training. | NA | Therapist Presence: Emphasizes the significance of the therapist's presence and how mindfulness can enhance attunement to clients' experiences.  Empathy and Compassion: Highlights how mindfulness fosters greater empathy and compassion within the therapeutic relationship.  Self-Awareness: Encourages therapists to develop self-awareness to manage countertransference and maintain therapeutic boundaries. | This book serves as a comprehensive guide for therapists interested in incorporating mindfulness into their practice. By focusing on the therapist's own mindfulness, the book provides a pathway to deeper therapeutic connections and more effective client outcomes. |
| 74 | Figley, C. R. |  | Treating compassion fatigue | Book | NA | NA | .  This book delves into the identification, assessment, and treatment of compassion fatigue, particularly among professionals in caregiving and trauma-related fields. Insights from various experts are compiled to address the challenges faced by those who experience secondary traumatic stress as a result of their empathetic engagement with traumatized individuals. | NA | Secondary Traumatic Stress: Explores the impact of indirect exposure to trauma on professionals, highlighting the emotional and psychological toll of empathetic engagement with survivors.  Therapeutic Interventions: Details various therapeutic modalities effective in treating compassion fatigue, such as the Accelerated Recovery Program (ARP), a multimodal brief treatment protocol for professional caregivers.  Professional Quality of Life: Discusses factors influencing the well-being of professionals, including work environment, support systems, and personal coping mechanisms. | This book guides professionals seeking to comprehend and mitigate the effects of compassion fatigue. Through its multidisciplinary perspectives and practical interventions, the book equips caregivers with the knowledge and tools necessary to maintain their well-being while effectively supporting those they serve |
| 75 | Mathieu, F. | 2011 | The compassion fatigue workbook: Creative tools for transforming compassion fatigue and vicarious traumatization | Book | NA | NA | This book is a practical guide designed to help professionals in caregiving roles recognize, understand, and transform compassion fatigue and vicarious traumatization. The workbook offers concrete strategies and experiential activities aimed at promoting self-care and resilience among those exposed to secondary trauma in their professional lives. | NA | Self-Care: Emphasizes the importance of self-care practices in preventing and addressing compassion fatigue, encouraging readers to prioritize their well-being.  Awareness and Education: Highlights the need for awareness and education about compassion fatigue among professionals and organizations to foster supportive environments.  Personal and Professional Balance: Encourages maintaining a healthy balance between personal life and professional responsibilities to sustain long-term effectiveness in caregiving roles. | This book serves as a comprehensive and user-friendly resource for individuals experiencing the emotional strains of caregiving professions. By providing practical tools and strategies, the author empowers readers to transform compassion fatigue into resilience, ensuring they can continue to provide compassionate care without compromising their own well-being. |
| 76 | West, C. P. et al. | 2014 | Intervention to promote physician well-being, job satisfaction, and professionalism: A randomized clinical trial | Randomised clinical trial | 12 | NA | Despite the documented prevalence and clinical ramifications of physician distress, few rigorous studies have tested interventions to address the problem. The objective is to test the hypothesis that an intervention involving a facilitated physician small-group curriculum would result in improvement in well-being | Randomized clinical trial of 74 practicing physicians in the Department of Medicine at the Mayo Clinic in Rochester, Minnesota, conducted between September 2010 and June 2012. Additional data were collected on 350 nontrial participants responding to annual surveys timed to coincide with the trial surveys. The intervention involved 19 biweekly facilitated physician discussion groups incorporating elements of mindfulness, reflection, shared experience, and small-group learning for 9 months. Protected time (1 hour of paid time every other week) for participants was provided by the institution. Meaning in work, empowerment and engagement in work, burnout, symptoms of depression, quality of life, and job satisfaction were assessed using validated metrics. | Empowerment and engagement at work increased by 5.3 points in the intervention arm vs a 0.5-point decline in the control arm by 3 months after the study (P = .04), an improvement sustained at 12 months (+5.5 vs +1.3 points; P = .03). Rates of high depersonalization at 3 months had decreased by 15.5% in the intervention arm vs a 0.8% increase in the control arm (P = .004). This difference was also sustained at 12 months (9.6% vs 1.5% decrease; P = .02). No statistically significant differences in stress, symptoms of depression, overall quality of life, or job satisfaction were seen. In additional comparisons including the nontrial physician cohort, the proportion of participants strongly agreeing that their work was meaningful increased 6.3% in the study intervention arm but decreased 6.3% in the study control arm and 13.4% in the nonstudy cohort (P = .04). Rates of depersonalization, emotional exhaustion, and overall burnout decreased substantially in the trial intervention arm, decreased slightly in the trial control arm, and increased in the nontrial cohort (P = .03, .007, and .002 for each outcome, respectively). | An intervention for physicians based on a facilitated small-group curriculum improved meaning and engagement in work and reduced depersonalization, with sustained results at 12 months after the study. |
| 77 | Dar | 2020 | Beyond linear evidence: The curvilinear relationship between secondary traumatic stress and vicarious posttraumatic growth among healthcare professionals | Cross- sectional study | 13.5 | NA | There is a lack of thorough and consistent research on the correlation between secondary traumatic stress (STS) and vicarious posttraumatic growth (VPTG). While some authors propose a linear connection between STS and VPTG, others argue for a curvilinear relationship. The aim of this study is to investigate whether the association between STS and VPTG is fundamentally linear or curvilinear. | The study utilized a cross-sectional correlational research design, involving a sample of 258 surgeons, psychiatrists, and physicians who work with victims of the ongoing conflict. | After accounting for the impact of vicarious and  direct traumatic exposure and experience, hierarchical regression results offer evidence supporting the curvilinear relationship between secondary traumatic stress (STS) and vicarious posttraumatic growth (VPTG) across the entire sample. The curvilinear model explained an additional 4.6% of the variance in Posttraumatic Growth Inventory (PTGI) scores compared to the linear model. This curvilinear association was also observed within specific professional groups, with the model explaining 4.3% and 3.5% of the variance in PTGI scores for surgeons and physicians, respectively, beyond what was explained by the linear models. | The results indicate that the highest levels of vicarious posttraumatic growth (VPTG) are most closely associated with moderate levels of secondary traumatic stress (STS). |
| 78 | Vaidya, A. | 2020 | Professional quality of life among medical doctors working in Kathmandu: A descriptive cross- sectional study | Cross- sectional study | 11.5 | NA | This study aims to investigate the professional quality of life among medical doctors working in the Kathmandu Valley, acknowledging the honorable nature of the medical profession alongside its challenging work environment. | A descriptive cross-sectional study was undertaken among 174 medical doctors in various hospitals within the Kathmandu Valley. Ethical approval was obtained from the Ethical Review Board of the Nepal Health Research Council. The research employed the WHO Professional Quality of Life Scale-5 to gather information on Compassion Satisfaction, Burnout, and Secondary Traumatic Stress among medical doctors practicing in the Kathmandu Valley. Data analysis was conducted using the Statistical Package for the Social Sciences version 16.0. | Among the 174 participants, 101 (58%) demonstrated a moderate level of Compassion Satisfaction, 126 (72.4%) exhibited Burnout at a moderate level, and 135 (77.6%) experienced a moderate level of Secondary Traumatic Stress. | More than half, almost two-thirds, and over two-  thirds of the participants demonstrated moderate levels of Compassion Satisfaction, Burnout, and Secondary Traumatic Stress, respectively. The overall findings of the study indicate a favorable balance between Compassion Satisfaction and Compassion Fatigue (comprising burnout and secondary traumatic stress) among Nepalese medical doctors. It is recommended to conduct a more extensive and multifaceted study to assess the professional quality of life of doctors and other healthcare workers. |
| 79 | Wong, W. K. T. et al. | 2018 | What lies beneath? Experiencing emotions and caring in oncology | Qualitative research study | NA | 19 | The aim of the study was to explore the medical encounter from the perspectives of oncologists, with a particular emphasis on the presence and experiences of emotions within clinical encounters. The study aimed to understand how emotions impact the doctor- patient relationship, the emotional experiences of oncologists, and the construction of their professional identity. | The methodology employed in this paper involved an interpretive approach to sociological research, using in-depth interviews with medical oncologists. 22 medical oncologists participated and interviewed.  Thematic analysis was used to identify emergent patterns in the transcripts, involving constant discussion within the research team to develop and examine themes. | The key findings of this paper include the  importance of emotional reflexivity in the experiences of oncologists during medical encounters, the concept of "bounded caring" as a site of expertise and professional identity, and the tension between professionalism and the experiences of emotions. The participants discussed the significance of establishing intimate connections with their patients, recognizing it as both a challenge and a potential opportunity. The study also highlights the evolving demands on oncologists and the enduring significance of emotional connections in medical encounters, emphasizing the co-production of caring experiences despite the challenges involved. | The proposed solutions in this paper include the recognition of the embedded emotionality within medical encounters and the need for oncologists to engage with their own emotions as well as those of their patients. The study suggests that this duality is likely to facilitate greater well-being for both parties. Additionally, it recommends that effective management of emotions in medical practice should consider the needs of both oncologists and patients, emphasizing the importance of recognizing and addressing emotions in oncology work. |
| 80 | Sprang, G. et al. | 2007 | Compassion Fatigue, Compassion Satisfaction, and Burnout: Factors Impacting a Professional's Quality of Life | Cross sectional study | 14 | NA | This research delves into the factors that could affect mental health professionals' responses to vicarious exposure to traumatic stress. It examines the concepts of compassion fatigue (CF), compassion satisfaction (CS), and burnout in a rural state, while also investigating the individual, occupational, and environmental factors that may influence the manifestation of these conditions. | The study used a quantitative approach to explore the impact of provider characteristics, practice characteristics, and rurality on the levels of compassion fatigue, burnout, and compassion satisfaction among mental health professionals in a rural state. It employed a survey instrument to collect data from 6,720 licensed or certified behavioral health providers, with 1,121 who completed the questionnaires. The researchers also used hierarchical regression analysis to assess the independent and combined contributions of various factors toward compassion fatigue, burnout, and compassion satisfaction. | The psychiatrists suffered from higher levels of CF than other professionals. The discovery of higher rates of compassion fatigue (CF) among psychiatrists sheds light on various factors contributing to the challenges faced by physicians in this study such as high workload and shortage of psychiatrists. The elevated CF scores for psychiatrists did not seem to be influenced by age or practice setting, as there were no significant differences in these demographic variables across disciplines. Additionally, the gender effects observed in the total sample did not apply to physicians. The increased levels of CF in this predominantly male group highlight the substantial impact of discipline on CF. | Lack of psychotherapy training may leave  physicians less capable of handling the stress they will face when dealing with challenging patients after completing their education. Proficiency in pharmacology does not shield against the emotional strain experienced when working closely with traumatized individuals. Specialized trauma training seems to offer clinicians some protection against compassion fatigue and enhances compassion satisfaction. Organizations working with traumatized patients are encouraged to promote such training and provide ongoing education opportunities. Educating clinicians about risk and protective factors, along with offering resources to enhance protection, could help reduce levels of compassion fatigue and burnout, with rural providers and psychiatrists potentially benefiting the most. Despite challenges related to organizational and managed care pressures, prioritizing capacity management and caseload mix development, particularly for clinicians treating patients with trauma and loss issues, would not only demonstrate compassion but also likely prove cost- effective in the long run by preventing staff leaving, reduced work time, and protective disengagement. |
| 81 | Ahmad H Almadani | 2022 | Compassion Fatigue Among Practicing and Future Psychiatrists: A National Perspective | Cross sectional study | 13 | NA | The study aimed to assess the levels of compassion satisfaction, burnout, and secondary traumatic stress, as well as to identify the sociodemographic and work- related factors associated with these outcomes. The ultimate goal was to provide insights that could contribute to the development of future management strategies to support practicing and future psychiatrists at risk of compassion fatigue. | The researchers conducted a cross-sectional study targeting all psychiatrists and psychiatric trainees registered in Saudi Arabia. They utilized a three- section questionnaire and the Professional Quality of Life Scale (ProQOL 5) to assess compassion satisfaction, burnout, and secondary traumatic stress. The survey collects demographic data, individual medical history, encompassing chronic medical or psychiatric conditions and experience of trauma; professional attributes aimed at evaluating work-related demographics, including years of experience, current job role, participation in managerial or teaching activities, and satisfaction with salary. | The findings revealed that a significant percentage  of participants experienced an average level of CF, while a lower percentage had a high level. Factors such as marital status, personal history of psychological trauma, and diagnosis of psychiatric illness were found to be associated with different levels of compassion satisfaction, burnout, and secondary traumatic stress. Moreover, it was noted that satisfaction with financial income was linked to lower burnout scores. The research highlighted the importance of developing systematic and proactive tools to screen for trauma and support practicing and future psychiatrists, especially those at risk of compassion fatigue. . For example, those who were divorced or separated exhibited higher levels of compassion satisfaction, while those diagnosed with psychiatric illnesses showed lower levels. | In conclusion, the study provided insights into the prevalence and risk factors of compassion fatigue among psychiatrists and psychiatric trainees in Saudi Arabia. The findings emphasized the need for effective support and interventions to address compassion fatigue and its associated factors. The proposed solutions include the development of screening tools, resiliency training, and further research to explore the impact of specific factors on compassion satisfaction and fatigue. |
| 82 | Rafael Mota Vargas | 2016 | The transformation process for palliative care professionals: The metamorphosis, a qualitative research study | Qualitative research study | NA | 20 | The document presents a qualitative research study on the professional trajectory of palliative care workers and the factors influencing this trajectory. It also identified factors influencing this trajectory and described the self-care strategies developed by these professionals. The study refers to this process as 'metamorphosis', leading to the formation of professionals who can work effectively within a palliative care context. The study provides insights into the transformation process experienced by palliative care professionals and the factors influencing their trajectory, offering valuable information for the practice, theory, and policy related to palliative care. | The study involved 10 palliative care professionals and revealed a shared path taken in their working lives: pre-palliative care, honeymoon, frustration, and maturation. The methodology used in this study is a qualitative research design based on the Grounded Theory approach. The data were collected through semi-structured individual interviews, which were audio-visually recorded and transcribed verbatim for analysis. The analysis of the data was conducted using the procedure described by Miles and Huberman, supported by the ATLAS.ti 6 software.  The study utilized a purposive sampling process to select a total of 10 palliative care professionals, and the data collection and analysis were conducted simultaneously. The methodology aimed to achieve a comprehensive understanding of the professional trajectory of palliative care workers and factors affecting this path. | The study examined the professional trajectory of  palliative care workers, focusing on the experiences and self-care strategies of doctors. The results revealed that doctors who transitioned into palliative care had previously worked in areas such as oncology, internal medicine, hospital emergency departments, intensive care units, primary care centers, and non-governmental organizations related to cancer. Before entering palliative care, they encountered patients in terminal situations and felt under-prepared for such scenarios. The study identified a common trajectory among doctors, including a "honeymoon stage" at the beginning of their palliative care work, a "frustration stage" resulting from differences between expectations and reality, and a "maturation stage" where they developed coping mechanisms and self-care strategies. Doctors expressed a range of emotions, including frustration, sadness, and fear, as well as positive emotions such as satisfaction and personal enrichment. | The study also highlighted the importance of self- care strategies for doctors, such as taking breaks for reflection, recognizing feelings and practicing self-control, and developing communication skills. The findings emphasized the need for comprehensive and specific training to support doctors in their palliative care roles. The study also underscored the significance of teamwork and the impact of relationships with other healthcare professionals on doctors' experiences in palliative care. Over time, they go through some struggles regarding the cost of caring and satisfaction of caring. Recognizing this potential risk and adopting self-care strategies can safeguard professionals and empower them to carry out their work effectively. In the course of this journey, influenced by various factors, they undergo a personal transformation that alters their outlook on life. |
| 83 | JOHN- HENRY PFIFFERLI NNG | 2000 | Overcoming compassion fatigue | article | NA | NA | The paper analyses the causes and symptoms of CF and shares strategies that physicians canacan adopt to overcome CF. It also advises physicians with CF on what they can and cannot do. | A 9 item questionnaire developed by the author is included inside and used to screen for CF. Participants who answer 4 or more questions with a yes may be suffering from CF. | The article shares many techniques on how to  overcome CF. The article details that individuals suffering from CF should follow a 3-step plan. spending quality time by themselves, recharging their batteries every day, and encourages them to have meaningful deep conversations every day. It advises individuals to form other hobbies outside of medicine and to pursue them. Additionally, it encourages them to spend time with family and friends. it also encourages individuals to share their feelings with a professional counsellor or peer support group so that they can be better listened to. | individuals need to accept that they are suffering from CF and start seeking help actively. They should identify what is important to them and avoid spending long hours at work or complaining to others. They need to identify what gives their life joy and meaning and pursue these self-care practices regularly. |
| 84 | Jan Coles | 2013 | “Preventing the Pain” When Working with Family and Sexual Violence in Primary Care | review article | NA | NA | The aim of the study is to address the challenges faced by primary care professionals (PCPs) in identifying and responding to family and sexual violence. It also aims to highlight the importance of recognizing, preventing, and managing stress among PCPs working with trauma victims, and to provide strategies at individual, organizational, and educational levels to improve the quality of care for survivors of family violence. Additionally, the study aims to emphasize the need for workplace policies, support systems, and training to enable PCPs to work effectively with trauma victims and prevent burnout and vicarious trauma among PCPs. | The authors draw upon their experiences conducting international workshops aimed at preventing trauma among professionals working with sexual violence cases. The article synthesizes expert opinions and insights gained from these workshops to propose strategies for supporting PCPs in managing the emotional toll associated with such work. | It is important to be aware of and recognise the  effects of VT on PCPs at both an individual and organisational level.  Address PCP's educational needs and provide ongoing support to minimize the potential for stress.  The paper emphasizes the importance of a professional culture that values and supports PCPs in providing care for survivors of family violence, as well as the need for workplace policies, support systems, and training to enable PCPs to work effectively with trauma victims.  Strategies for managing stress among PCPs include ongoing development of skills, spreading the workload, and implementing self-care strategies such as physical fitness, psychological strategies, spirituality, and staying connected.  The paper highlights the need for undergraduate and postgraduate training of PCPs to develop trauma knowledge and skills to ask about and respond to family violence safely, as well as the importance of ensuring that adequate counseling and effective therapy services are available to support the work of PCPs. | The paper advocates for integration of training regarding burn out, VT and its implications into training curiculum of all PCPs who work with sexual violence. It recommends developing workplace policies that recognize secondary trauma as a potential workplace health risk, address related stigma, normalize workplace stress, and outline appropriate responses. The paper emphasizes the importance of setting up recognized "family violence teams" at work for all who work with family violence, providing peer-peer support, and the ability to discuss difficult or distressing cases. It also suggests providing adequate support and supervision, including discussion of secondary trauma and its implications, and ensuring managers are appropriately trained to recognize and respond to secondary trauma in a supportive, nonpunitive manner. The paper highlights the importance of  self-care strategies for PCPs, including physical  fitness, psychological strategies, spirituality/ advocacy, and staying connected. |
| 85 | Bowling, John M | 2022 | Effect of COVID-19 Delta (B.1.617.2) and Omicron (B.1.1.529) surges on emergency m medicine physician compassion | cross sectional study | 14 | NA | The aim of this study is to investigate compassion fatigue and compassion satisfaction in emergency medicine physicians during the COVID-19 surges. The study aims to bring awareness to the emotional challenges experienced by emergency medicine physicians during the pandemic. | The methodology in this study involved the use of a survey to investigate compassion fatigue (CF) and compassion satisfaction (CS) in emergency medicine physicians during the COVID-19 surges. The survey included the Professional Quality of Life Scale (ProQOL), using 30 questions to objectively measure CF and CS, along with 3 questions on COVID-19 and a textbox provided for any comments. Completed responses were obtained from 608 EM physicians, from all regions of the United States. The differences in scores were analyzed using statistical analysis and reported in terms of mean and standard deviation. | EM physicians experienced lower levels of  compassion satisfaction and higher levels of secondary traumatic stress during the COVID-19 surges compared to pre-COVID investigations. This indicates a negative impact on physician well- being.  ProQOL scores varied significantly across age, gender, and years in practice, suggesting that these factors can influence the experience of compassion fatigue  The majority of respondents felt that their answers to the survey would have been different before the COVID-19 pandemic, indicating the specific impact of the pandemic on physician well- being.  Additional stressors reported by physicians include fear of exposure, patient vaccination status, and the politicization of the pandemic. | The study strongly underscores the imperative to acknowledge and tackle compassion fatigue. It advocates for fostering support and awareness among healthcare providers as crucial measures in combating compassion fatigue. |
| 86 | Kiran, Wajiha | 2023 | Relationship between Secondary Trauma Self-Efficacy, Secondary Trauma and Job Burnout among Doctors Dealing with COVID-19 Patients | cross sectional study | 15 | NA | The research investigated the correlation among secondary trauma self-efficacy, job burnout, and secondary trauma in doctors who are involved in the care of COVID-19 patients. | The study enlisted 78 doctors serving as frontline workers in Lahore, Pakistan, amid the COVID-19 pandemic. Data collection was carried out through an online Google form, and statistical analyses were performed using SPSS software. | The findings demonstrated a significant adverse  association between secondary trauma self- efficacy and compassion fatigue. Additionally, a negative correlation was observed between secondary trauma self-efficacy and both secondary traumatic stress and job burnout among doctors involved in the care of COVID-19 patients.  Specifically, the study revealed that secondary trauma self-efficacy accounted for 19% of the variability in secondary traumatic stress and 17% of the variability in job burnout among physicians working with COVID-19 patients. Furthermore, the study found correlations between satisfaction with salary and working hours, subjective experiences of stress, anxiousness, and sadness, and the variables under investigation. The results indicated that elevated levels of compassion fatigue, job burnout, and secondary trauma were positively associated with higher levels of stress,  anxiousness, and sadness in doctors. | In conclusion, the study indicated that doctors  involved in the care of COVID-19 patients face a heightened risk of secondary trauma and burnout, potentially resulting in elevated stress, diminished mood, and increased anxiousness. However, the research underscored that secondary trauma self- efficacy can function as a protective factor against these challenges. The findings underscore the significance of addressing compassion fatigue in healthcare workers, especially those at the forefront of the COVID-19 pandemic.  Furthermore, the study proposed the necessity for additional research to explore potential gender differences in compassion fatigue and to delve into the causal factors associated with these variables. It also advocated for more comprehensive, manual investigations to thoroughly examine the factors contributing to compassion fatigue among doctors working with COVID-19 patients. |
| 87 | Alavi, Sepideh M | 2024 | Addressing burnout in healthcare: Understanding barriers and facilitators to health promotion and implementing a wellness programming for resident physicians | primary qualitative study | - | 23 | The main aim of this study is to understand the barriers and facilitators involved in implementing a wellness program to prevent and address burnout in physicians. It’s important to clarify what a supportive workplace environment looks like for trainees, residents, fellows, and early-career physicians, as they are among the most vulnerable healthcare providers. Using the Rapid Assessment Procedure Informed Clinical Ethnography (RAPICE), the study will investigate how the Thriving Organizations Thriving Staff (TOTS) wellness program is applied to resident physicians in a healthcare setting. Additionally, the research will explore the challenges healthcare organizations face in tackling burnout and the factors that support health promotion, from the viewpoints of key informants and resident physicians. Lastly, the study will look into the concept of health promotion and how healthcare organizations can become trauma-informed, fostering a culture of health for trainees such as residents and fellows. | The study utilizes a community-based participatory research (CBPR) approach to explore the barriers and facilitators impacting burnout among resident physicians. It employs a qualitative exploratory design that integrates ethnographic methods and semi-structured interviews for data collection. Participants in the study consist of two primary groups: key informants and resident physicians. Key informants include stakeholders from the healthcare sector who offer insights into the organizational challenges associated with burnout. Resident physicians are selected from healthcare systems where the wellness program is being implemented. Data collection involves conducting semi-structured interviews with key informants, which are designed to delve into their perspectives on burnout and wellness initiatives. Additionally, the study incorporates ethnographic observations during the implementation of the wellness program, allowing researchers to document the experiences and interactions of resident physicians in real-time. This combination of methods aims to provide a comprehensive understanding of the factors influencing burnout and the effectiveness of wellness programming in healthcare settings. | The study reveals several key findings regarding burnout among resident physicians and the effectiveness of wellness programs. Firstly, the impact of COVID-19 has significantly intensified existing challenges, leading to increased levels of burnout and secondary traumatic stress among healthcare providers. Participants expressed feelings of isolation and noted that the ongoing high-stress environment, coupled with evolving perceptions of healthcare workers, has adversely affected their mental well-being. Secondly, barriers to seeking help for mental health issues emerged as a prominent theme. Stigma surrounding mental health, time constraints, and the internalized pressure to maintain a facade of competence hindered many physicians from reaching out for assistance. As a result, a culture of silence regarding mental health struggles persisted among healthcare professionals. Additionally, the importance of supportive leadership was emphasized by participants. They highlighted that effective leadership plays a crucial role in fostering an environment conducive to wellness. Regular check-ins and open lines of communication from supervisors were identified as vital strategies to alleviate burnout and promote overall well-being. Finally, the study underscores the necessity for structured wellness programs tailored to the unique challenges faced by resident physicians. Participants stressed the importance of psychoeducation about burnout, peer support, and opportunities for debriefing and team-building as essential components of effective wellness initiatives. Overall, these findings point to the urgent need for healthcare organizations to prioritize mental health and implement comprehensive support systems for their staff. | The study proposes several solutions to address burnout among resident physicians and enhance their overall well-being. One key solution is the enhancement of leadership training, which would equip healthcare leaders with the skills to recognize signs of burnout and foster open communication about mental health issues. Additionally, the research advocates for the establishment of ongoing wellness initiatives that incorporate psychoeducation about burnout, facilitating peer support and creating opportunities for debriefing and team-building among healthcare professionals. Furthermore, there is a strong emphasis on the need for structured support systems that specifically address the unique challenges faced by resident physicians. This includes cultivating an environment where seeking help is normalized and encouraged. The study encourages a cultural shift within healthcare organizations, asserting that mental health should be prioritized as a fundamental concern rather than treated as secondary.  In conclusion, the research highlights the urgent necessity for healthcare organizations to prioritize the mental health and well-being of their staff. By implementing comprehensive wellness programs tailored to the specific challenges of resident physicians, organizations can effectively combat burnout. The study underscores that fostering supportive leadership, providing ongoing wellness initiatives, and creating a culture that prioritizes mental health are essential steps toward improving the overall work environment for healthcare professionals. |
| 88 | Renita Ann Mercado Heinzl | 2023 | Assessment of the impact of compassion satisfaction, resilience, support, and self-care practices in preventing or mitigating the development of secondary traumatic stress, vicarious traumatization, compassion fatigue, and burnout on nurses and physicians in an advanced network of care hospital in the Midwest | mixed methods study | 12 | 14 | This research explores how healthcare professionals, including nurses and doctors, are affected by repeated exposure to patient trauma, potentially leading to secondary traumatic stress, vicarious traumatization, compassion fatigue, and burnout. Additionally, it examines the role of specific protective factors in alleviating or curbing these emotional and mental challenges in caregiving. | This study took place in a faith-based community hospital in the Midwest, utilizing an ethnographic mixed-methods approach. A total of six nurses and six physicians participated, completing two quantitative assessment tools and taking part in a single interview that included both quantitative and qualitative questions. | The findings of the study revealed that participants experienced different levels of secondary traumatic stress, vicarious traumatization, compassion fatigue, and burnout. Additionally, protective factors such as compassion satisfaction, supportive resilience, self-care practices, and religious or spiritual beliefs played a role in preventing or reducing these effects. Lastly, the study examined the types of self-care behaviors participants engaged in to promote health and overall well-being. | The study offers both short-term and long-term recommendations, emphasizing the need for continued staff education on trauma, resilience, and self-care. It also highlights the importance of raising awareness about holistic support resources available to healthcare teams and underscores the role of management in actively addressing staff concerns and incorporating their feedback. Additionally, the findings reinforce the significant role that spiritual and religious beliefs play in the healing process. Chaplains are identified as valuable resources in helping team members recognize and navigate the emotional complexities of caregiving, including both its rewards and challenges. They can also support staff in managing their trauma through avenues such as compassion satisfaction, resilience, self-care, and spirituality. |
| 89 | Gabriela Fernandez-Miranda | 2023 | Compassion and decision fatigue among healthcare workers during COVID-19 pandemic in a Colombian sample | cross sectional study | 13.5 | - | In this study, we aimed to examine levels of compassion and decision fatigue during the pandemic and identify the factors influencing these forms of fatigue. | We gathered information through self-reported questionnaires measuring compassion fatigue, decision fatigue, and grit across five periods from April to November 2020, with a sample size of 856 participants. | Our findings revealed a inverse relationship between grit and both compassion and decision fatigue, suggesting that individuals with higher resilience tend to experience less exhaustion. Additionally, we observed that grit levels were generally higher among technicians, nurses, psychologists, social workers, and emergency room (ER) staff, while lower in general practitioners. Compassion fatigue was more pronounced in technicians, whereas decision fatigue was lower in specialists, general practitioners, and technicians but higher among those working in private hospitals. | In conclusion, our study provides a framework for understanding healthcare workers’ experiences during the early COVID-19 pandemic. We found an inverse relationship between grit and both compassion and decision fatigue, which is significant for two main reasons. First, our study is the first to show this link between grit and fatigue in a crisis setting. This highlights the need to explore these relationships further to develop interventions that address fatigue in healthcare workers during crises. Improving these factors is key to supporting healthcare workers’ well-being and, by extension, their patients. Second, the impact of the pandemic varied depending on role and work environment. This shows that the effects of rising cases were not the same for all healthcare workers, with some facing greater challenges than others in undetermined times. |
| 90 | Juan P Roman-Calderon | 2024 | Compassion and Self-Compassion: Counterfactors of Burnout in Medical Students and Physicians | cross sectional study | 13.5 | - | This studies aims to explore the connection between self-compassion, compassion for others, and burnout among medical students and doctors . | This study utilized a cross-sectional design, involving medical students and general practitioners from two cities in Colombia (n = 359). Participants completed the Compassion Scales and the Maslach Burnout Inventory. To validate new assessments and analyze the connections between underlying factors, an Exploratory Structural Equation Modeling approach was applied. | The majority of participants were medical students (85.9%) with an average age of 22 years (SD = 7). Women made up 55.2% of the sample, and 62.6% spent over 48 hours per week studying or practicing. Physicians had an average of 10.34 years of experience (SD = 8.67). While the self-compassion and compassion-for-others action subscales were validated, the engagement subscales were not. Actions of compassion toward others had an inverse relationship with depersonalization, while self-compassion actions were inversely linked to emotional exhaustion. Additionally, compassion-related factors showed a direct association with professional fulfillment. | Our results indicate that acts of compassion and self-compassion are inversely linked to various aspects of burnout and may help shield healthcare professionals from stress. Implementing training programs that foster these qualities could be a potential strategy to prevent burnout in medical students and physicians by lowering depersonalization and emotional fatigue while improving their sense of professional achievement. |
| 91 | Seema Jain | 2024 | Compassion Fatigue Amongst Resident Doctors of a Medical College | cross sectional study | 14 | - | This study aims to evaluate the occurence of compassion fatigue among resident doctors and explore its connection to various work-related reasons. | Doctors at a Tertiary Care Center in Meerut participated in this cross-sectional study. A total of 200 residents from various medical specialties and departments provided data through a questionnaire. This included the Professional Quality of Life Scale, along with details on their sociodemographic background and work-related factors. | Residents with more years of practice showed higher levels of compassion satisfaction. However, those working long hours, particularly with frequent overnight shifts, seemed to be at greater risk of experiencing compassion fatigue, which was linked to increased burnout and secondary traumatic stress. | Evaluating compassion fatigue in resident doctors underscores the influence of working conditions on their well-being. By addressing issues such as extended work hours, heavy patient loads, and inadequate administrative support, compassion fatigue among resident doctors can be reduced. |
| 92 | Zahra Moradi | 2024 | Compassion fatigue and moral injury in physicians of intensive care unit (ICU) of private hospitals in Shiraz, Iran | cross sectional study | 14 | - | This study focused on the relationship between compassion fatigue and moral injury among critical care physicians in intensive care units. Additionally, it evaluated the levels of compassion fatigue and moral injury in these physicians. | The research used a cross-sectional design and involved 310 physicians working in the intensive care units of private hospitals in Shiraz, Iran. Physicians with at least one year of experience in the medical field and no history of physical or mental illness were included in the study. Exclusion criteria consisted of refusal to participate and incomplete questionnaires. Data collection involved three demographic profile questionnaires, the Figley Compassion Fatigue Questionnaire, and Cap's Moral Injury Questionnaire. Participants who verbally agreed to take part in the study were given printed questionnaires, which they completed and returned in full to the researcher. The collected data were entered and stored using SPSS version 27. For detailed analysis, Pearson correlation, ANOVA, and multiple regression tests were conducted based on the predefined criteria. | On the compassion satisfaction subscale, physicians scored an average of 36.54±5.66, while they scored 29.11±6.07 on the compassion fatigue subscale and 30.01±6.51 on the secondary traumatic stress subscale. Analyzing the data, we found a significant inverse relationship between compassion satisfaction and moral injury (p<0.001), meaning that higher compassion satisfaction was linked to lower levels of moral injury. Additionally, compassion fatigue showed a significant direct relationship with moral injury (p<0.001), indicating that as compassion fatigue increased, so did moral injury. A similar direct and significant relationship was observed between secondary traumatic stress and moral injury (p<0.001), where an increase in secondary traumatic stress corresponded to a rise in moral injury. | The observed inverse relationship between compassion satisfaction and moral injury indicates that higher job satisfaction can reduce moral challenges. In contrast, the positive associations found between compassion fatigue, secondary traumatic stress, and moral injury highlight the urgent need for interventions aimed at addressing the psychological strain on healthcare workers. Such measures are essential to improve resilience in these high-demand environments. These findings reinforce the importance of creating a healthy work environment that fosters peer support, care, and well-being among physicians, ensuring they can effectively meet the demands of patients in critical and intensive care units. |
| 93 | Joshua Campbell | 2022 | Compassion fatigue and satisfaction among turkish emergency medicine residents using the professional quality of life scale | cross sectional study | 13.5 | - | Burnout syndrome is prevalent among emergency medicine (EM) residents. The Professional Quality of Life scale (ProQOL), which includes measures for compassion satisfaction (CS), burnout, and secondary traumatic stress (STS), is a validated tool to assess these factors. This study aimed to evaluate the levels of CS and fatigue among Turkish EM residents using the ProQOL survey. | This cross-sectional study targeted Turkish EM residents who are members of the EM Residency Association of Turkey. The ProQOL survey version 5 was emailed in Turkish to all 150 EM residents. The participants, who were currently working as EM residents, also provided demographic data and indicated their satisfaction with their quality of life. | The survey was completed by 80 residents. Almost 50% of the respondents expressed dissatisfaction with their overall quality of life. The Turkish EM residents exhibited moderate levels of CS (33.9 ± 7.9), as well as moderate levels of burnout (27.0 ± 5.9) and STS (24.7 ± 5.3). | The results suggest that Turksih EM residents experience moderate levelz of both compassion satisfaction and burnout, along with secondarry traumatic stress |
| 94 | Tomoe Nishihara | 2022 | Compassion fatigue in a health care worker treating COVID-19 patients: A case report | Case report | - | - | This case report aims to highlight the psychological burden faced by a physician treating severe COVID-19 cases, specifically focusing on compassion fatigue and emotional distress resulting from prolonged exposure to critically ill patients and their family. | The study presents the case of a 29-year-old female doctor who developed anxiety and insomnia while caring for critically ill COVID-19 patients. The physician was responsible for treating a 47-year-old man who succumbed to the virus and his 76-year-old mother, who experienced acute stress disorder following her son's death. The mother's initial refusal of treatment was later managed through psychiatric intervention, leading to her recovery and discharge. During the course of these events, the attending doctor exhibited symptoms of psychological distress, including anticipatory anxiety and sleep disturbances. Hospital administrators provided structured workplace support, and she underwent psychotherapy for compassion fatigue, which facilitated her recovery. | The physician experienced severe emotional exhaustion and psychological distress due to the emotional weight of caring for terminally ill patients and their grieving families. Her symptoms of compassion fatigue included difficulty sleeping and heightened anxiety. With appropriate mental health intervention and workplace support, she was able to recover and return to work. | This case emphasizes the mental health challenges faced by healthcare workers managing critically ill COVID-19 patients. Excessive emotional engagement in patient care, especially in cases with poor prognoses, increases the risk of compassion fatigue. Recognizing the psychological toll on healthcare providers is crucial to implementing effective support systems that prioritize their mental well-being. |
| 95 | Donald Jeanmonod | 2024 | Compassion Fatigue in Emergency Medicine: Current Perspectives | Systematic review | - | - | Compassion fatigue (CF) refers to the diminished ability to empathize with or care for patients, a growing issue among emergency physicians (EPs). Due to the demands of their profession, EPs work in unpredictable environments, often during irregular hours, and are frequently exposed to traumatic situations. These physicians face significant psychological, physical, and cognitive stress, with limited time for recovery or self-reflection. When prolonged workplace strain leads to feelings of exhaustion, helplessness, lack of support, and an inability to manage responsibilities, CF develops.  Both direct exposure to traumatic events, such as workplace violence (primary traumatic stress), and emotional distress from witnessing patient suffering (secondary traumatic stress or STS) increase the likelihood of experiencing CF. If not properly managed, CF can lead to serious consequences, including lower patient care quality, decreased patient satisfaction, higher rates of physician depression, anxiety, and substance abuse, as well as greater physician turnover within emergency medicine. | - | To combat CF, both individual physicians and healthcare organizations must implement preventive measures. EPs can strengthen their resilience by practicing cognitive-behavioral techniques and mindfulness, maintaining their physical health, finding fulfillment in their work, engaging in hobbies outside their medical career, and establishing clear boundaries between professional and personal life. | On an institutional level, hospitals must address major contributors to burnout, such as excessive workloads, insufficient resources, lack of autonomy, workplace culture, and work-life imbalance. Organizations must also ensure safer working conditions to minimize primary traumatic stress and offer adequate psychological support to EPs struggling with STS, while actively reducing the stigma associated with seeking help for post-traumatic symptoms. |
| 96 | Anna  Garnett | 2023 | Compassion fatigue in healthcare providers: a scoping review | Systematic review | - | - | The COVID-19 pandemic has had lasting negative effects on the mental health and well-being of healthcare workers, significantly influencing their professional responsibilities and increasing the likelihood of compassion fatigue. This review aimed to compile and analyze existing research on compassion fatigue among healthcare providers and its consequences for patient care. | A comprehensive search was conducted across six databases—MEDLINE (Ovid), PsycINFO (Ovid), Embase (Ovid), CINAHL, Scopus, and Web of Science—to identify studies published in English between 2020 and 2023, covering the peak and aftermath of the pandemic. To ensure a broader scope, reference lists from selected studies were manually reviewed for additional relevant literature. The review primarily examined research on nurses, physicians, and allied health professionals. | Following the Preferred Reporting Items for Systematic Reviews and Meta-Analyses (PRISMA) extension for scoping reviews, this study was registered on the Open Science Framework (OSF). Out of 11,715 search results, 24 studies met the eligibility criteria. The findings were categorized into four key themes: the prevalence of compassion fatigue, its contributing factors, its impact on healthcare providers, and strategies to mitigate it. The identified causes of compassion fatigue were classified into individual, organizational, and systemic factors. The review also highlighted that the risk of developing compassion fatigue varies between countries. Effective interventions, such as increasing staffing levels, were found to reduce the occurrence of compassion fatigue. | This scoping review provides valuable insights into the common triggers, risks, and management strategies for compassion fatigue in healthcare professionals. It also underscores the need for targeted support systems to safeguard their psychological well-being and ensure high-quality patient care. |
| 97 | Ibrahim HM Alharbi | 2023 | Compassion fatigue, burnout and compassion satisfaction among physicians in Makkah region, Saudi Arabia: a cross sectional study | Cross-sectional study | 14 | - | This research investigates the prevalence of Compassion Fatigue (CF), Burnout (BO), and Compassion Satisfaction (CS) among physicians practicing in the Makkah region of Saudi Arabia. | To assess these factors, an online survey was distributed to physicians across various specialties, including Residents, Specialists, and Consultants. The questionnaire aimed to measure the levels of CF, BO, and CS among participants. | Findings revealed that workplace physical conditions and challenging clinical scenarios were the primary sources of stress among physicians. The study identified CF in 18.5% of respondents, while 15.6% experienced BO. Additionally, 17.7% of participants reported CS. | The study highlights a rising prevalence of CF and BO alongside declining CS levels among physicians. To address this issue, greater attention should be given to identifying the contributing factors and implementing strategies to mitigate their effects. |
| 98 | Dinesh Bhugra | 2025 | Compassion fatigue: result or cause of burnout? And do doctors get it? | Grey Lit | - | - | - | - | - | Recognizing compassion fatigue is the essential first step in addressing it, and colleagues can play a crucial role in this process. Since prevention is preferable to treatment, every physician should remain vigilant for signs of compassion fatigue and take proactive steps to manage their well-being.   This includes prioritizing self-care by allocating personal time, engaging with friends and family, maintaining a nutritious diet, and ensuring sufficient quality sleep. Participating in physical and mental relaxation activities such as exercise, yoga, meditation, mindfulness, or listening to music can also be beneficial.   Compassion fatigue is preventable. Developing compassion resilience begins with understanding the condition. Seeking peer support, professional guidance, taking breaks, and spending time with loved ones are effective strategies for coping with and reducing compassion fatigue. |
| 99 | Qing Ye | 2024 | Empathy fatigue among physicians and its influencing factors: a cross-sectional survey from Southwest China | Cross-sectional study | 13.5 | - | Empathy fatigue occurs when medical professionals experience excessive emotional strain while caring for patients, leading to trauma and emotional exhaustion. In severe cases, this condition can contribute to medical errors, disputes, and worsening doctor-patient relationships. While most research on empathy fatigue has focused on nurses, studies on its impact on physicians remain limited. The goal of this study was to explore empathy fatigue in clinicians from southwest China and provide empirical evidence to aid in its prevention and treatment. | A cross-sectional survey was conducted using convenience sampling to assess empathy fatigue among physicians working in public tertiary hospitals in southwest China. | A total of 562 physicians participated in the study. The average scores for empathy satisfaction disorder, job burnout, and secondary traumatic stress were 32.1±6.85, 28.2±5.30, and 26.2±6.04, respectively. The findings revealed that 291 physicians (51.8%) suffered from severe empathy fatigue. Physicians working two to three night shifts per week were more likely to experience severe empathy fatigue. Additionally, 424 participants (75.4%) reported having thoughts of resigning, with factors such as limited rest time, empathy satisfaction disorder, job burnout, and secondary traumatic stress contributing to these thoughts. | Empathy fatigue is widespread among hospital physicians, with many considering leaving their positions. This study highlights the urgent need for interventions to address empathy fatigue and improve physician retention. |
| 100 | Gabriela Vieira Lucio | 2024 | Exploring the Correlation Between Palliative Care Training in Brazil and Compassion Fatigue: A Cross-Sectional Study | Cross-sectional study | 15 | - | This study aimed to assess the prevalence of compassion fatigue among general practitioners with and without palliative care training. It also explored the experiences of these physicians. | The research employed a quantitative and qualitative cross-sectional survey, with data analyzed using Mann-Whitney and Pearson's chi-squared tests, considering P < 0.05 as significant. | The findings showed that a significant proportion of respondents (77.8%) lacked formal training in palliative care. However, 58.3% reported caring for at least one palliative care patient every two shifts, which represented a moderate frequency in the study. Trauma levels among the physicians were classified as medium (24 ± 6), burnout was low (19 ± 5), and compassion was medium (41 ± 5). Physicians without palliative care training exhibited higher levels of burnout (P = 0.002), though no significant associations were found between training and trauma or compassion levels. | In conclusion, general practitioners in Brazil who received palliative care training showed lower burnout rates in secondary hospitals. This suggests the potential benefits of introducing palliative care educational programs within hospitals to enhance physician performance and patient care. No differences were observed between trauma and compassion scores. |
| 101 | Carlie Nikel | 2024 | Finding compassion when compassion fatigued | Qualitative study | - | 12 | The unrelenting turmoil of the COVID-19 pandemic has been especially hard on those in the healthcare field. This chronic stress has resulted in depleted compassion towards patients served and colleagues. Researchers have suggested that empathy is a finite resource that must be replenished. For physicians in residency training and those in practice, where finding meaning and purpose in their work is already a challenge, the spark for medicine and helping others may easily become extinguished. This article explores interventions implemented in two family medicine residency programs to address concerns about burnout and compassion fatigue exacerbated by the COVID-19 pandemic. | A qualitative research approach was used, focusing on case studies from the two residency programs. The study involved 36 family medicine residents from "Residency #1" and another 36 from "Residency #2." | The research highlights various strategies employed within these programs to mitigate compassion fatigue, including meditation, gratitude exercises, guided group support sessions, and an emphasis on physical well-being. Feedback from both residents and program leaders indicated that these approaches played a valuable role in helping physicians adjust to the evolving demands of practicing medicine during the pandemic, ultimately reducing burnout and the impact of compassion fatigue. Additionally, a strategic framework is provided to assist other training programs in implementing similar initiatives. | It is imperative that training programs continue to implement inter- ventions that emphasize resident and post-residency wellness to reduce compassion fatigue in order to navigate the new stressors that came with the COVID-19 pandemic and those that will come with pandemics in the future. |
| 102 | Justin Wong | 2025 | Responding to vicarious trauma experienced by junior doctors during hospital-based rotations: A narrative review considering effective approaches that have been used in healthcare settings | Narrative review | - | - | Psychological debriefing continues to be implemented and advocated as a reactive approach to limit the effects of vicarious trauma. However, the literature strongly suggests this approach does not reduce the risk of developing post-traumatic stress disorder and may worsen symptoms. To identify alternatives to supporting junior doctors, a narrative literature search was conducted across multiple databases to identify relevant information. |  | Alternatives to psychological debriefing included resilience training, mindfulness-based intervention, Schwartz rounds and somatic experiencing. While these novel interventions are promising, caution must be exercised, the evidence base is limited. | Exposure to distressing events may be unavoidable for junior doctors. As hospitals look for better ways to address vicarious trauma, caution must be exercised to follow the evidence rather than implement a solution-focused ideology that may be harmful. Our findings suggest the need to focus on proactive rather than reactive approaches. Further research is needed. |
| 103 | Melissa Bales | 2022 | Quantifying Compassion Fatigue in Ancillary and Clinical Staff in an Adult Emergency Department | Cross Sectional | 9 | - | Emergency department (ED) staff face a high risk of compassion fatigue (CF) due to the demanding environment, which includes high patient acuity, violence, and various workplace stressors. This complex condition can lead to significant mental health issues, such as depression, anxiety, and substance use disorders, if not addressed. However, most studies on CF focus only on clinicians, leaving a gap in understanding its impact on other roles that support patient care. This study aimed to determine the prevalence of CF across both clinical and non-clinical roles in the adult ED setting. | This single-institution, cross-sectional study included all full- and part-time ED staff who worked at least 50% of their shifts in the ED or within the adult trauma service line. The Professional Quality of Life Scale was used to assess compassion fatigue (CF), which includes compassion satisfaction (CS), burnout (BO), and secondary traumatic stress (STS). To analyze role differences, a non-parametric one-way ANOVA was conducted. | A total of 152 participants completed the survey, including attending physicians, resident/fellow physicians, staff nurses, emergency technicians, supportive clinical staff, and supportive ancillary staff. Most respondents reported average levels of burnout (BO) and secondary traumatic stress (STS), along with high levels of compassion satisfaction (CS). There was a significant difference in CS by role, with nurses reporting lower CS than attending physicians. STS also varied by role, with attending physicians experiencing lower STS than emergency technicians and nurses. No significant differences were found in BO across roles. | Compassion fatigue subcomponents were found to be similar across all ED team members, including non-clinical staff. It is recommended that programs to identify and address compassion fatigue be introduced and extended to all roles within the ED. |
| 104 | Tariq Noman M. Alanazi | 2021 | Reported effects of the COVID-19 pandemic on the psychological status of emergency healthcare workers: A scoping review | Scoping Review | - | - | While there is an abundance of literature on the psychological consequences faced by frontline healthcare workers (HCWs), understanding the psychological burden on this group is particularly important due to their heightened risk from exposure to COVID-19. This study aimed to explore the psychological effects of the COVID-19 pandemic on emergency HCWs. | The scoping review following the guidelines outlined in the Preferred Reporting Items for Systematic Reviews and Meta-Analyses extension for Scoping Reviews. | The review included 21 studies and found that emergency healthcare workers (HCWs) experienced anxiety, burnout, depression, PTSD symptoms, stress, and secondary trauma. Physicians and nurses reported higher levels of anxiety, burnout, depression, and stress compared to other roles. PTSD symptoms were more common among reserve medics, while Red Cross volunteers had similar psychological stress to other HCWs. Additionally, male HCWs reported more PTSD symptoms, while female HCWs experienced higher stress levels. | Emergency healthcare workers (HCWs) providing care during the COVID-19 pandemic are vulnerable to various psychological impacts, including anxiety, burnout, depression, inadequate sleep, PTSD symptoms, psychological distress, secondary trauma, and stress. |
| 105 | Lynne McCormack | 2023 | Risk of burnout, psychological growth, longevity of career and making sense of Covid-19 in senior Australian radiation oncologists | Qualitative study | - | 21 | Senior radiation oncologists working in hospital or organizational settings are frequently exposed to the traumatic experiences of others, which heightens their risk of burnout. However, the extra organizational challenges posed by the COVID-19 pandemic and their effects on these professionals' mental health and long-term career sustainability remain poorly understood. | Using Interpretative Phenomenological Analysis, semi-structured interviews were conducted to gather both positive and negative subjective data from five senior Australian radiation oncologists during the COVID-19 lockdowns. | The study revealed one central theme, "Vicarious risk, hierarchical invalidation, redefining altruistic authenticity," which was supported by four subordinate themes: (1) Vicarious contamination of caring, (2) The hierarchical squeeze, (3) The heavy burden of me, and (4) Growth of authenticity. Participants faced dual challenges to their career longevity and mental well-being: balancing their role as empathic caregivers to vulnerable patients with the increasing demands of the organization. This led to feelings of invalidation, exhaustion, and disengagement. However, as they gained experience and seniority, they began to prioritize self-care, nurturing it through personal honesty, altruism, and meaningful connections with both patients and junior colleagues. By focusing on mutual well-being, they eventually found it acceptable to envision a life beyond their roles in radiation oncology. | For these participants, self-care evolved into a relational connection with their patients, distinct from the lack of systemic support. This absence of support contributed to an early end to their careers, as they prioritized psychological well-being and authenticity. |
| 106 | Hvovi Bhagwagar | 2022 | Secondary trauma, burnout and resilience among mental health professionals from India: A review of research | Systematic Review | - | - | To review and synthesize existing research on burnout, secondary traumatic stress, and resilience among Indian mental health professionals (MHPs). | A systematic review of 14 peer-reviewed studies (2005–2022) covering burnout, vicarious trauma, compassion fatigue, and resilience in psychiatrists, psychologists, psychiatric nurses, social workers, and counselors. | Findings highlight sociodemographic influences, psychological impacts, and risk/protective factors affecting burnout and resilience. Resilience is linked to well-being, coping, and stress management. | The review identifies research gaps and provides recommendations for improving theoretical understanding and practical interventions for MHPs in India. |
| 107 | Chuqian Chen | 2024 | Suicide among psychiatrists: From healers to seekers of mental health care | Cross-sectional | 12 | - | Patient deaths significantly affect caregivers both professionally and personally. This study investigates the relationship between subjective and objective experiences of patient deaths and different aspects of professional quality of life (ProQOL) in physicians and nurses. | This study analyzed cross-sectional data from 306 Chinese physicians and nurses who had experienced a patient death at least one month prior. Objective experiences were assessed by the number of past patient deaths, while subjective experiences were measured using the Accumulated Global Changes (AGC) subscale of the Professional Bereavement Scale. Professional quality of life (ProQOL) was evaluated using the Professional Quality of Life Scale. Regression analyses were conducted following bivariate analyses. | The number of past patient deaths showed no significant association with any of the three ProQOL scores in both bivariate and regression analyses. However, higher AGC scores correlated with increased burnout, secondary traumatic stress, and compassion satisfaction, even after controlling for factors such as age, occupation, department, work experience, job commitment, and sense of mission. | Subjective, rather than objective, patient death experiences are significantly associated with all three aspects of ProQOL in physicians and nurses. Caregivers who perceive greater personal change from past patient deaths report higher levels of burnout and secondary traumatic stress but also greater job satisfaction and fulfillment in their helping role. |
| 108 | Jacob Fox, Barry Meisenberg | 2022 | The 3-Fold Harms of Compassion Fatigue During COVID-19 Surges | Grey Lit | - | - | - | - | Healthcare workers are facing increasing compassion fatigue and burnout due to prolonged stress, overwhelming patient loads, and limited recovery time. The challenge is further compounded by frustration in treating unvaccinated patients, which creates moral distress and threatens the principle of providing care without judgment. The erosion of empathy has serious consequences, including higher medical errors, increased healthcare costs, and declining patient trust. Additionally, burnout takes a toll on mental health, career satisfaction, and the overall integrity of the profession. | To address this crisis, it is crucial to replenish empathy and support healthcare workers. Strategies such as wellness programs, peer support, structured discussions (e.g., Balint groups), and fostering professional solidarity can help mitigate burnout. Ultimately, upholding ethical principles and providing equal care to all patients is essential to maintaining both public trust and the integrity of the medical profession. |
| 109 | Wen Qing Wendy Ye | 2023 | The impact of patient death experiences early in training on resident physicians: a qualitative study | Qualitative study | - | 27 | Patient death is an unavoidable aspect of medical training, often leading to psychological distress, reduced empathy, and poorer learning outcomes. This study examines how early-career residents experience patient death, including its immediate and long-term effects, their level of preparedness, and the coping strategies they use. The goal is to identify gaps and opportunities for better supporting trainees during these challenging experiences. | This qualitative study used a phenomenological approach to explore residents' personal experiences with patient death. Internal medicine residents at McMaster University who had completed a rotation were invited to participate between December 2020 and April 2021. Semi-structured interviews were conducted to examine the circumstances, emotional impact, support systems, coping strategies, and preparedness related to patient death. The interviews were transcribed, coded, and analyzed thematically to identify key patterns and insights. | Interviews with resident physicians revealed common challenges and emotional responses to patient death. Many had encountered their first patient death during medical school, yet they often felt unprepared for the experience. Difficult situations included unexpected deaths, pronouncing death, performing CPR, and communicating with families. These events led to feelings of guilt, helplessness, and grief, sometimes resulting in sleep disturbances, intrusive thoughts, and emotional detachment. Despite these struggles, participants described such experiences as a normalized part of training, highlighting the need for better support and preparation. | Experiencing patient death during medical training can be traumatic, leading to emotional distress, loss of empathy, and changes in clinical practice. Educational programs that focus on preparing trainees and teaching adaptive coping strategies may help reduce psychological impact. Further research is needed to explore effective approaches for supporting trainees through these experiences. |
| 110 | Nur Nihal Türkel | 2025 | The imposter phenomenon in psychiatrists: relationships among compassion fatigue, burnout, and maladaptive perfectionism | Cross-sectional study | 9 | - | This study aimed to test a model examining the relationships between burnout, compassion fatigue, maladaptive perfectionism, and the imposter phenomenon in mental health professionals. | This descriptive, cross-sectional study surveyed 160 psychiatrists online between June and September 2023. Path analysis was used to examine relationships among the study variables and evaluate the overall model fit. | The study found a strong correlation between the imposter phenomenon, burnout, and compassion fatigue, even after controlling for age and work experience across both genders. Maladaptive perfectionism influenced the imposter phenomenon both directly and indirectly through its impact on burnout and compassion fatigue. | The study concluded that burnout and maladaptive perfectionism significantly contribute to the imposter phenomenon in psychiatrists. Addressing this issue requires reevaluating societal norms that reinforce burnout and perfectionism in mental health professionals. |
| 111 | Yun Ting Ong | 2024 | Towards a Clinically Relevant Appreciation of the Cost of Caring: A Study of Palliative Care Physicians in Malaysia | Qualitative study | - | 20 | This study redefines the cost of caring, expanding beyond compassion fatigue to include burnout, secondary traumatic stress, vicarious trauma, and reduced compassion satisfaction. Increasing patient demands and emotional burdens expose physicians to stressors that blur professional and personal boundaries, leading to declining job satisfaction and care quality. | This study explores a clinically relevant understanding of the cost of caring among palliative care physicians in Malaysia. Using the Systematic Evidence-Based Approach (SEBA), 11 physicians participated in semi-structured interviews to share their experiences. | The analysis identified three key domains: understanding the cost of caring, risk factors, and support mechanisms. | This study suggests that the cost of caring among Malaysian palliative care physicians extends beyond moral distress, compassion fatigue, vicarious trauma, secondary traumatic stress, and burnout. Instead, it is a personalized experience, shaped by individual and contextual factors that evolve over time. To address this, the study proposes a longitudinal, personalized, and holistic mentoring program. |
| 112 | Annushkha Sinnathamby | 2025 | Towards a Theory of Compassion Fatigue in Palliative Care and Oncology: A Systematic Scoping Review | Scoping review | - | - | Palliative care physicians and oncologists face compassion fatigue (CF) due to complex care needs, evolving practice demands, and sociocultural factors. However, research on CF remains limited. This study proposes a systematic scoping review (SSR) to explore existing theories of CF in physicians. | Using the PRISMA-based Systematic Evidence-Based Approach (SEBA), this systematic scoping review (SSR) analyzed articles published from 2000 to 2023 across MEDLINE, EMBASE, PsycINFO, Wiley, CINAHL, and Google Scholar. The study applied thematic and content analyses to synthesize findings. | Out of a total of 10,505 titles reviewed, 80 articles were selected for inclusion. The analysis of 15 current compassion fatigue (CF) theories identified two primary categories: those focused on CF itself and those examining the impacts of caregiving. These CF theories have developed from Figley’s initial model, gradually integrating concepts such as moral distress, vicarious trauma, and burnout. More recent theories also take into account factors like individual characteristics, decision-making processes, and self-awareness. | Compassion fatigue (CF) is recognized as a broader aspect of the cost of caregiving, connecting clinical experiences with personal concepts of identity and personhood. The Ring Theory of Personhood offers valuable insight into how physicians react to these experiences, playing a crucial role in guiding physician support and fostering supportive, nurturing work environments. |
| 113 | Antonio Kobayashi-Gutiérrez | 2024 | Validation of the scale compassion fatigue inventory in health professional Spanish-speaking: a cross-sectional study | Cross-sectional | 9.5 | - | The Compassion Fatigue Inventory was validated for Spanish-speaking healthcare workers by assessing its psychometric properties and examining the factors that influence it. | In this cross-sectional validation study, data was collected from 733 nurses, physicians, and medical residents using several instruments, including the Compassion Fatigue Inventory (CFI), Secondary Traumatic Stress Questionnaire (CETS), Physician Burnout Syndrome Scale (PhBS), Quality-of-Life Index (SQL-sp), and the Reduced Moral Stress Scale (MMDHPr). The CFI's psychometric properties were assessed through exploratory factor analysis (EFA), confirmatory factor analysis (CFA), and internal consistency evaluation. Additionally, the final version of the translated CFI was correlated with other scales. | An exploratory factor analysis (EFA) was conducted to assess the translated CFI, which identified 15 items across three dimensions: reduced compassion, social life, and workplace, as described in the literature. The confirmatory factor analysis (CFA) revealed good fit indices and strong psychometric values. The CFI demonstrated a negative correlation with the SQL-sp and a positive correlation with the other subscales. Additionally, work experience was found to be a predictor of a slight decrease in CFI scores. | The adaptation of the CFI for a sample of Latino healthcare professionals demonstrated satisfactory psychometric indices in assessing compassion fatigue. It can be considered a useful tool for distinguishing compassion fatigue from other occupational syndromes. |
| 114 | Noemi Sanso | 2019 | Differential Effects of Two Contemplative Practice-based Programs for Health Care Professionals | Quasi-experimental design | 15 | - | The study aimed to evaluate the effectiveness of two different interventions, Mindfulness-Based Stress Reduction Training (MBSRT) and Compassion Cultivation Training (CCT), in improving the quality of life of primary care professionals in Majorca. | A total of 50 participants took part in the research, with 81% being women. The study assessed mindfulness, empathy, self-compassion, and professional quality of life before and after the interventions. | Results showed that both programs led to significant improvements in mindfulness, self-compassion, and professional quality of life. However, the interventions had varying effects on specific aspects of mindfulness and professional well-being. The MBSRT group demonstrated greater improvements in acting with awareness and non-reactivity to inner experiences, as well as a more substantial reduction in burnout levels compared to the CCT group. | The study concludes that both interventions are effective in enhancing psychological and professional well-being, with MBSRT proving to be more effective in these areas for this group of healthcare professionals. |
| 115 | Ranjita Howard | 2019 | Personal resilience in psychiatrists:  systematic review | Systematic review | - | - | This systematic review aims to explore factors affecting personal resilience among  psychiatrists, in particular, those that may impair well-being and those that facilitate  resilience practice. | A literature search was performed of the Ovid®, Embase®,  CINAHL and PsycINFO databases, using keywords to identify empirical studies  involving psychiatrists that examined resilience, stress and burnout from the past  15 years. | Thirty-three international English language studies were included, showing  that a combination of workplace, personal and non-workplace factors negatively and  positively influenced well-being and resilience. | Given that workplace factors were the most commonly cited, it would appear that any resilience package that predominantly targets interventions at the workplace level would be particularly fruitful. Future research, however, needs to address the absence of a universal measurement of well-being and its moderators so that any potential interventions are better evaluated. |
| 116 | Alexander Hodkinson | 2022 | Associations of physician burnout with career engagement and  quality of patient care: systematic review and meta-analysis | Systematic review | - | - | This study examined the association of physician burnout with  career engagement and the quality of patient care. | Medline, PsycINFO, Embase, and CINAHL were  searched from database inception until May 2021. Data  were double extracted by independent reviewers and  checked through contacting all authors, 84 (49%) of  170 of whom confirmed their data. Random-effect  models were used to calculate the pooled odds  ratio, prediction intervals expressed the amount of heterogeneity, and meta-regressions assessed for  potential moderators with significance set using a  conservative level of P<0.10. | 4732 articles were identified, of which 170  observational studies of 239246 physicians were  included in the meta-analysis. Overall burnout in  physicians was associated with an almost four  times decrease in job satisfaction compared with  increased job satisfaction. In the presence of  increased burnout, career choice regret increased by  more than threefold compared with being satisfied  with career choice. Turnover intention also increased by more than threefold compared with retention. | This meta-analysis provides compelling evidence that  physician burnout is associated with poor function and sustainability of healthcare organisations  primarily by contributing to the career disengagement  and turnover of physicians and secondarily by  reducing the quality of patient care. |
| 117 | Terri Babineau | 2019 | Physician Burnout  and Compassion Fatigue:  Individual and Institutional  Response to an Emerging Crisis | Review | - | - | This paper reviews the recent literature in burnout and compassion  fatigue focusing on institutional factors influencing burnout where providers work as well as the individual factors affecting providers. In this review, there is an evaluation of the causes and interventions of burnout and compassion fatigue and areas for intervention. | - | The percentage of those suffering from burnout is decreasing for the first time in several years, and there are interventions to help decrease the impact of burnout and compassion fatigue. For an individual provider, mindfulness, cognitive behavioral therapy, and improved communication appear to be productive approaches to help with burnout. Institutional approaches that mitigate the impact of burnout in healthcare include assessing provider needs and returning some autonomy and control over workload to the provider. | Burnout and compassion fatigue remain a significant problem in healthcare and  impact patient care as well as the well-being of providers. There are approaches that are  working to decrease this impact in both the individual provider as well as the institutions in which providers are working. The research in this area from an individual perspective and a business perspective is only beginning. |
| 118 | Natalie Pei Xin Chan | 2022 | Extending the Ring Theory of Personhood to the Care  of Dying Patients in Intensive Care Units | Review | - | - | This study adopted Radha Krishna’s Ring Theory of Personhood (RToP) to scrutinise the experiences of physicians working in intensive care units (ICU) using a fictional scenario that was inspired by real events. The impact of death and dying, its catalysts, internal constituents, external factors, dyssynchrony, and buffers, specific to ICU physicians, were identified and explored. Such a framework allows for ramifications to be considered holistically and facilitates the curation of strategies for conflict resolution. | - | - | - |
| 119 | Michelle C. Nguyen | 2019 | Training on mind-body skills: Feasibility and  effects on physician mindfulness, compassion, and  associated effects on stress, burnout, and clinical  outcomes | Quantitative study | 11.5 | - | This study examines the feasibility and effectiveness of a Mind-Body Skills training (MBST) curriculum in promoting physician compassion and mindfulness, and reducing stress and burnout. | Participants were offered up to 7 hours of training: four free online modules on MBST and three interactive discussion sessions. Primary outcomes included feasibility and improvements in mindfulness and compassion. | Of the 66 participants, 50 (76%) completed pre- and post-intervention questionnaires. Most (62%) completed at least one of seven hours of training. Compared with participants who used zero hours of training, those who completed at least one hour of training had significant mean differences in compassion (p = 0.05), burnout (p = 0.05), and emotional exhaustion (p < 0.01). | These findings suggest that even brief Mind-  Body Skills training curriculum for physicians is feasible, and is observed to be associated with improvements in compassion and reductions in burnout. |
| 120 | Isabelle Bragarda | 2015 | Quality of work life, burnout, and stress in emergency  department physicians: a qualitative review | Qualitative review | - | - | A 2006 literature review reported that emergency department (ED) physicians showed elevated burnout levels and highlighted several environment and personal issues contributing toward burnout. Research on burnout in EDs is limited. This study proposed an updated qualitative review on  the relationships between work stress, burnout, and quality of work life in ED physicians. | MEDLINE, PsycInfo, and Science Direct were searched for studies published since 2005. Of 491 papers, 10 papers were retained, using  validated measures and having a minimum of 75  participants. Data extraction was performed manually by the first author and was reviewed by the second author. | ED physicians showed moderate to high levels of burnout with difficult work conditions including significant psychological demands, lack of resources, and poor support. Nonetheless, physicians reported high job satisfaction. | The ED physicians in the studies reviewed here showed  a moderate to high level of burnout. Researchers and  administrators in this area should be aware that symptoms of burnout constitute potential precursors of more severe impairment, including alcohol abuse, drug abuse, and suicidal ideation.  Further studies should focus on the implementation of measures designed to prevent burnout. |
| 121 | Leigh V Evans | 2021 | Improving patient and clinician safety during COVID-19 through rapidly adaptive simulation and a randomised  controlled trial: a study protocol | Mixed methods study Protocol | 13.5 | 22 | At the front lines, emergency physicians also face continued personal safety concerns and increased work burden, which heighten stress and anxiety, especially  given the prolonged course of the pandemic. Burnout, already a serious concern for emergency physicians due to  the cumulative stresses of their daily practice, may present as a longer-term outcome of these acute stressors. | Semi-structured qualitative interviews and human factor observations were conducted to determine the challenges and facilitators of COVID-19 preparedness and mitigation of physician stress. Next, a randomised controlled trial was conducted to test the effectiveness of a simulation preparedness intervention on physician physiological stress as measured by decreased heart rate variability on shift and anxiety as measured by the State-Trait Anxiety Inventory. | Medical simulation provides the  opportunity for standardised practice for high-stakes  events and the identification of latent safety threats.  We will develop a packaged set of immersive simulations based on qualitative data from staff participants and guidance from the departmental COVID-19 ED Task Force. These scenarios will then be delivered as just-in-time simulations to prepare physicians working within the subsequent week. | Outcomes of the current work will both address the  needs of clinicians in the current COVID-19 pandemic as well as provide a blueprint for achieving system readiness. If proven successful, the CRI:SIS rapidly adaptive simulation programme will be readily scalable and applicable at other institutions to improve and evaluate the responsiveness of healthcare delivery systems and healthcare professionals to the COVID-19 pandemic. Likewise, as a simulation-based continuous process improvement programme, CRI:SIS will provide a model for simulation-based preparedness to maintain readiness and incorporate lessons learnt from the COVID-19 pandemic into future system responses. |
| 122 | Matthew Siedsma | 2015 | Physician burnout: can we make a difference together? | Randomized clinical trial | 14 | - | Despite the documented prevalence and clinical ramifications of physician distress, few rigorous studies have tested interventions to address the problem. This study aimed to test the hypothesis that an intervention  involving a facilitated physician small-group curriculum would result in improvement in well-being. | A randomized clinical trial of practicing physicians. Additional data were collected on non-trial participants responding to annual surveys timed to coincide with the trial surveys. The study involved 74 practicing physicians in the Department of Medicine and 350 nontrial partici-  pants responding to annual surveys. The intervention involved 19 biweekly facilitated physician discussion groups incorporating elements of mindfulness, reflection, shared experience, and small-group learning for 9 months. Protected time (1 hour of paid time every other week) for participants was provided by the institution. | Rates of depersonalization, emotional exhaustion, and overall burnout decreased substantially in the trial intervention arm, decreased slightly in the trial control arm, and increased in the non-trial cohort (P = .03, P = .007, and P = .002 for each outcome,  respectively). | An intervention for physicians based on a facilitated  small-group curriculum improved meaning and engagement in work and reduced depersonalization, with sustained results 12 months after the study. |
